# Genetic dissection of yield-related traits and mid-parent heterosis for those traits in maize (*Zea mays* L.)

**DOI:** 10.1186/s12870-019-2009-2

**Published:** 2019-09-09

**Authors:** Qiang Yi, Yinghong Liu, Xianbin Hou, Xiangge Zhang, Hui Li, Junjie Zhang, Hanmei Liu, Yufeng Hu, Guowu Yu, Yangping Li, Yongbin Wang, Yubi Huang

**Affiliations:** 1 State Key Laboratory of Crop Gene Exploration and Utilization in Southwest China, Sichuan Agricultural University, Chengdu, 611130 China; 2 College of Agronomy, Sichuan Agricultural University, Chengdu, 611130 China; 3 Maize Research Institute, Sichuan Agricultural University, Chengdu, 611130 China; 4 College of Agriculture and Food Engineering, Baise University, Baise, 533000 Guangxi China; 5 College of Life Science, Sichuan Agricultural University, Ya’an, 625014 China

**Keywords:** Heterosis, Grain yield, Immortalized F_2_ population, Recombinant inbred lines, Quantitative trait locus

## Abstract

**Background:**

Utilization of heterosis in maize could be critical in maize breeding for boosting grain yield. However, the genetic architecture of heterosis is not fully understood. To dissect the genetic basis of yield-related traits and heterosis in maize, 301 recombinant inbred lines derived from 08 to 641 × YE478 and 298 hybrids from the immortalized F_2_ (IF_2_) population were used to map quantitative trait loci (QTLs) for nine yield-related traits and mid-parent heterosis.

**Results:**

We observed 156 QTLs, 28 pairs of loci with epistatic interaction, and 10 significant QTL × environment interactions in the inbred and hybrid mapping populations. The high heterosis in F_1_ and IF_2_ populations for kernel weight per ear (KWPE), ear weight per ear (EWPE), and kernel number per row (KNPR) matched the high percentages of QTLs (over 50%) for those traits exhibiting overdominance, whereas a notable predominance of loci with dominance effects (more than 70%) was observed for traits that show low heterosis such as cob weight per ear (CWPE), rate of kernel production (RKP), ear length (EL), ear diameter (ED), cob diameter, and row number (RN). The environmentally stable QTL *qRKP3–2* was identified across two mapping populations, while *qKWPE9*, affecting the trait mean and the mid-parent heterosis (MPH) level, explained over 18% of phenotypic variations. Nine QTLs, *qEWPE9–1*, *qEWPE10–1*, *qCWPE6*, *qEL8*, *qED2–2*, *qRN10–1*, *qKWPE9*, *qKWPE10–1*, and *qRKP4–3*, accounted for over 10% of phenotypic variation. In addition, QTL mapping identified 95 QTLs that were gathered together and integrated into 33 QTL clusters on 10 chromosomes.

**Conclusions:**

The results revealed that (1) the inheritance of yield-related traits and MPH in the heterotic pattern improved Reid (PA) × Tem-tropic I (PB) is trait-dependent; (2) a large proportion of loci showed dominance effects, whereas overdominance also contributed to MPH for KNPR, EWPE, and KWPE; (3) marker-assisted selection for markers at genomic regions 1.09–1.11, 2.04, 3.08–3.09, and 10.04–10.05 contributed to hybrid performance per se and heterosis and were repeatedly reported in previous studies using different heterotic patterns is recommended.

**Electronic supplementary material:**

The online version of this article (10.1186/s12870-019-2009-2) contains supplementary material, which is available to authorized users.

## Background

Utilization of heterosis in maize is of great importance for boosting grain yield [1–4]. Hybrids in maize accounted for 65% of total maize cultivation by the late twentieth century and had contributed to a quadrupling of annual maize production [1]. Numerous breeders have been interested in heterosis for many years; however, comprehension of the genetics implicated in heterosis for grain yield still remains elusive. Therefore, investigating and assessing genetic mechanisms of maize heterosis for grain yield would provide a paved route for understanding those phenomena and help to optimize breeding for grain yield in different heterotic groups.

Through earlier inbred selection and experimental breeding [5, 6], maize breeders realized the importance of germplasm resources and heterosis. Previous studies [2, 7–13] used molecular markers, phenotypic identification, and pedigree information to divide different germplasm into different heterotic groups. The heterotic pattern Stiff Stalk Synthetic (SS) × Non-Stiff Stalk (NSS) has been widely used for maize breeding [12, 14–16]. Li and Wang [17], based on former studies, proposed five heterotic groups, namely Tangsipingtou (TSPT), Lancaster, Lancaster-like, the improved Reid (PA), and Tem-tropic I (PB). Extensive research has been focused on the TSPT × Reid heterotic pattern because it is one of the predominant heterotic patterns in northern China and has been used for the production of elite hybrids such as Zhengdan958 [18]. However, this heterotic pattern is rarely used in southwestern China, where alternative, scarcely investigated, heterotic patterns such as PB × PA are more suitable [17]. Therefore, the current study investigated the genetics of heterosis for yield-related traits in a PB × PA hybrid and the genetics of the yield-related traits per se.

Heterosis is defined as the superiority of F_1_ hybrids in their performance over their parents [19, 20] and has been attributed to three different genetic hypotheses: dominance [21, 22], overdominance [19, 23], and/or epistasis [24, 25]. Specific loci for heterosis reported in previous studies corroborated each one of these hypotheses [26–38]. Those loci were identified using different designs such as the North Carolina design III with F_3_ families or recombinant inbred lines (RILs), immortalized F_2_ (IF_2)_ populations, or chromosome segment substitution lines (CSSLs). Those studies suggested different degrees of dominance for grain yield and inconsistency of heterotic loci across different heterotic patterns. For example, Frascaroli et al. [29] used the RILs from a cross between two elite inbred lines, B73 and H99, and three testcross populations obtained by crossing 142 RILs to each parent and F_1_ found a significant correlation between heterozygosity level and phenotypic performance. Schön et al. [32] demonstrated high congruency of QTLs for heterosis of grain yield in three populations derived from the crosses of the heterotic pattern Iowa Stiff Stalk × Lancaster Sure Crop and proposed that diverse alleles were fixed in each heterotic group. Tang et al. [31] identified 13 heterotic loci for yield-related traits, and Guo et al. [34] reported that the genetic basis of heterosis for grain yield of the elite maize hybrid Yuyu22 relies on the cumulative effects of dominance, overdominance, and epistasis. Li et al. [37] detected 38 heterotic loci for ear-related traits and suggested a genotype-dependent combination of heterotic loci in different hybrids. Notably, Wang et al. [35, 38] identified 169 heterotic loci associated with grain yield and yield components in two CSSL test populations. Therefore, the results pertaining to genetics of grain yield and heterosis using crosses from well-studied heterotic patterns, such as Reid × Lancaster and TSPT × Reid, cannot be extrapolated to other heterotic patterns such PA × PB.

In this study, in order to dissect genetic architecture of grain yield-related traits in maize and the contribution of different genetic effects to heterosis for grain yield-related traits, we evaluated 301 RILs from 08 to 641 × YE478 in four environments and an IF_2_ population produced from those RILs under three environments. The elite line YE478, belonging to the PA heterotic group [17], is one of the hybrid foundation parents in China. The other parent, 08–641 (SAU08–641), which is derived from PB (or Tem-tropic I heterotic group) germplasm, is a typical elite inbred line widely used in southwest China since 1998. PA × PB is one of the predominant heterotic patterns in southwestern China. The main objectives of this research were (1) to detect and estimate QTLs with additive and additive-additive effects on yield-related traits using an RIL population (2) to identify QTLs with additive, dominant, and/or epistatic effect on yield-related traits using a hybrid population derived from the RIL population (IF_2_ population) (3) to identify QTLs for mid-parent heterosis (MPH) using both mapping populations and (4) to identify the most suitable genomic regions associated with grain yield and MPH to be managed by marker-assisted selection.

## Results

### The performance of grain yield-related traits and heterosis across environments

**Fig. 1:**
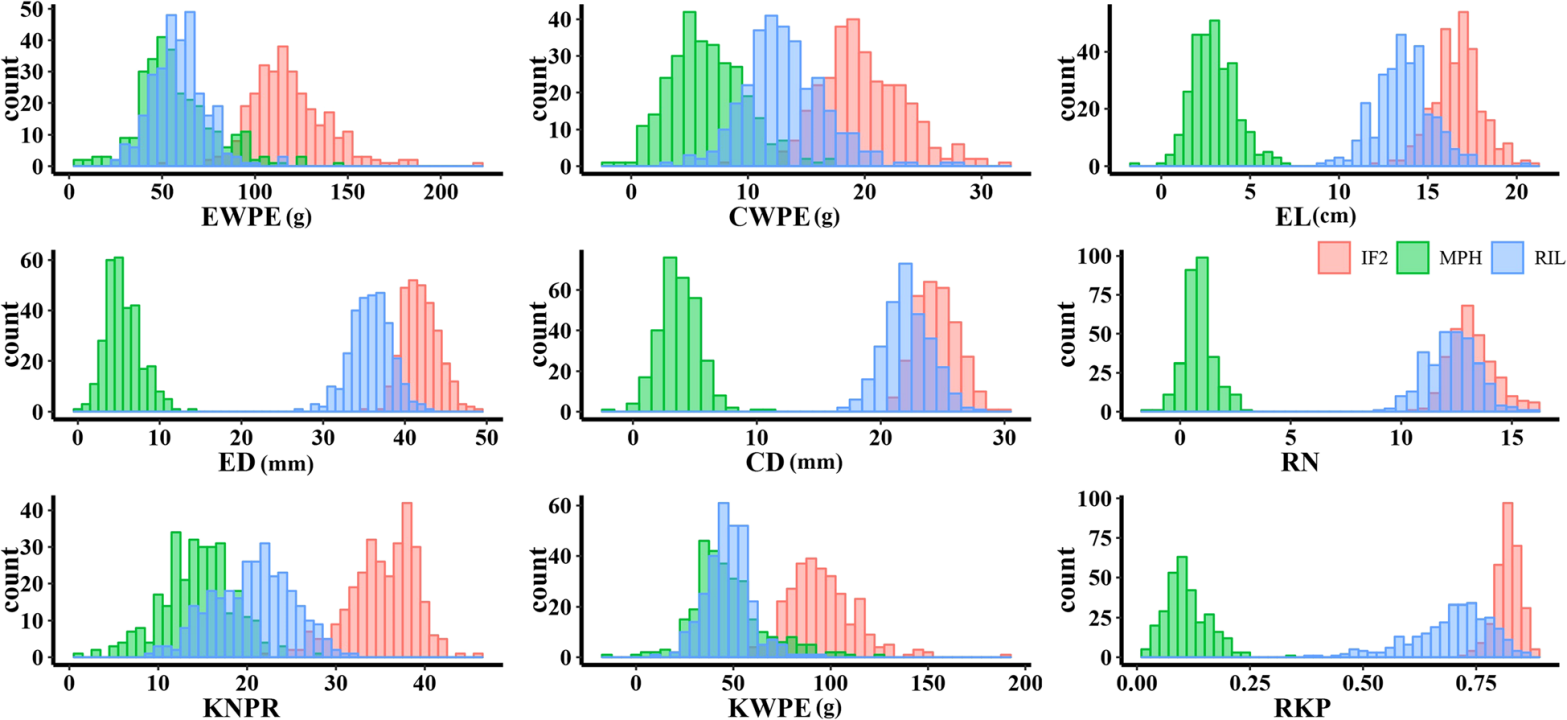
The histogram for yield-related traits and mid-parent heterosis (MPH) for those traits in the RILs and the IF_2_ population. EWPE, ear weight per ear; CWPE, cob weight per ear; EL, ear length; ED, ear diameter; CD, cob diameter; RN, row number; KNPR, kernel number per row; KWPE, kernel weight per ear; RKP, rate of kernel production

The parental line Ye478 had significantly higher mean RKP and kernel number per row (KNPR) compared with those of 08–641 (Additional file 1: Figure S1). The RIL and IF_2_ populations showed considerable variations for yield-related traits (Fig. 1). The means of the F_1_ were significantly higher than the means of any of the parental lines for ear weight per ear (EWPE), cob weight (CWPE), ear length (EL), ear diameter (ED), kernel weight per ear (KWPE), KNPR, and RKP. MPH of the hybrid Ye478 × 08–641 was < 16% for ED and RKP, 43.5% for EL, and > 70% for EWPE, KWPE, CWPE, and KNPR. Average MPH values across hybrids of the IF_2_ population were similar to MPH values for F_1_ (Additional file 4: Table S2). These results suggested a strong heterosis level for EWPE, KWPE, KNPR, and CWPE. The RIL and IF_2_ populations thus seem suitable for dissection of genetic effects involved in yield-related traits and heterosis for these traits.

**Fig. 2:**
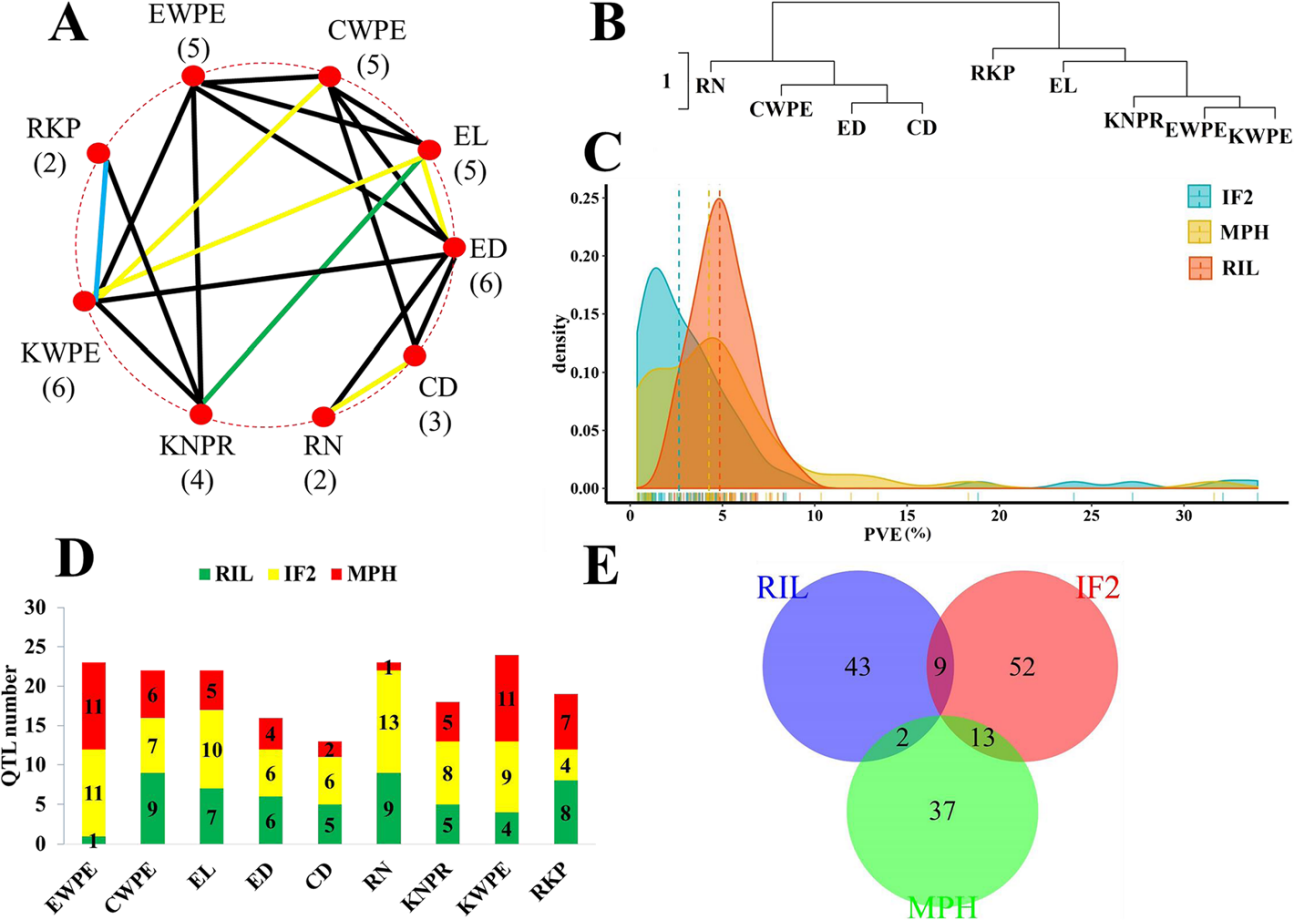
The correlation relationship among yield-related traits, and mid-parent heterosis (MPH) for those traits, and QTL distribution in the different datasets. **a** Pearson’s correlation coefficients among yield-related traits and MPH (|*r*^*2*^| > = 0.5). The yellow lines indicate significant correlation found in the MPH dataset; Blue line indicates significant correlation in the RILs; green line indicates significant correlation in both the IF_2_ and the MPH dataset; black lines indicate significant correlations found across the RILs, the IF_2_ and the MPH dataset. The number below each trait indicates the number of the correlated traits. **b** Hierarchical cluster analysis (“hclust”) of phenotypic traits based on the standardized data in the RILs. The short-scale indicates Euclidean distance. **c** Frequency distribution of QTLs with PVE (phenotypic variance explained by each QTL) detected in the RILs, IF_2_ population_,_ and the MPH dataset. The vertical and dashed lines indicate the median values. **d** The QTL numbers for nine yield-related traits identified in the RILs, IF_2_ population_,_ and the MPH dataset. **e** Venn diagram for the 156 QTLs detected using averaged means for yield-related traits (EWPE, ear weight per ear; CWPE, cob weight per ear; EL, ear length; ED, ear diameter; CD, cob diameter; RN, row number; KNPR, kernel number per row; KWPE, kernel weight per ear; RKP, rate of kernel production) across environments in the RILs and IF_2_ populations_,_ and the MPH dataset

In the combined analyses of variance of the RIL and IF_2_ populations across all environments, the sources of variation for genotype, environment, and genotype × environment interaction were highly significant (*p* < 0.01) for most yield-related traits (Table 1). The broad-sense heritability (*h*^2^) for these traits ranged from 60.8% for KWPE to 91.1% for RN. Heritability for EWPE and KWPE was greater in the IF_2_ population than in the RIL population. The correlations among most yield-related traits and among MPH values for those traits were significant (Fig. 2a; Additional file 5: Table S3). EWPE in the RIL and IF_2_ population was moderately correlated with CWPE, KWPE, EL, ED, and KNPR (Pearson’s correlation coefficients > 0.5). MPH for EWPE was moderately and positively correlated with MPH for CWPE, KWPE, EL, ED, and KNPR. The hierarchical cluster analysis based on the standardized data from the RILs classified the yield-related traits into two unrooted groups, with traits within each group being, in general, moderately to highly correlated based on Pearson’s correlation coefficients (Fig. 2b).

**Table 1:** Mean squares from the combined analyses of variance in the RILs under four environments and the IF_2_ population across three environments

Trait^a^	Population	Gen^b^	Env^b^	Rep (Env)^b^	Gen × Env^b^	*h*^2^ (%)^c^	C. I (%)^d^
EWPE	RIL	1059.24^**^	472,851.69^**^	0.04	397.37^**^	62.5	55.6–68.2
	IF_2_	2055.73^**^	1,080,285.18^**^	24.60^**^	445.28^**^	78.3	74.2–81.7
CWPE	RIL	65.39^**^	6542.52^**^	57.93^**^	17.89**	72.6	67.6–76.8
	IF_2_	62.46^**^	8119.14^**^	25.87^**^	14.63^**^	76.6	72.2–80.2
EL	RIL	18.86^**^	293.57^**^	0.17	2.65^**^	85.9	83.5–87.9
	IF_2_	9.66^**^	959.04^**^	6.97^**^	1.33^**^	86.2	83.6–88.3
ED	RIL	41.48^**^	5547.70^**^	0.08^**^	11.97^**^	71.1	66.2–75.2
	IF_2_	23.54^**^	5304.38^**^	8.90^**^	6.01	74.5	69.7–78.4
CD	RIL	26.37^**^	4715.00^**^	7.81	7.55^**^	71.3	66.4–75.4
	IF_2_	14.27^**^	461.95^**^	1.6	4.7	67.1	60.9–72.1
RN	RIL	9.73^**^	63.01^**^	0.15	0.87^**^	91.1	89.5–92.3
	IF_2_	5.40^**^	74.82^**^	0.1	0.5^**^	90.8	89.1–92.2
KNPR	RIL	103.85^**^	16,419.65^**^	8.330	28.42^**^	72.6	67.6–76.8
	IF_2_	73.69^**^	12,860.29^**^	1.89	19.65^**^	73.3	68.3–77.4
KWPE	RIL	740.61^**^	195,608.04^**^	152.290	290.12^**^	60.8	53.9–66.5
	IF_2_	1429.09^**^	833,331.84^**^	28.24^**^	315.94^**^	77.9	73.8–81.3
RKP	RIL	0.05^**^	2.62^**^	0.00	0.01^**^	77.7	73.8–80.9
	IF_2_	0.00^**^	0.54^**^	0.68	0.00^**^	71.3	66.0–75.7

^a^*EWPE* Ear weight per ear, *CWPE* Cob weight per ear, *EL* Ear length, *ED* Ear diameter, *CD* Cob diameter, *RN* Row number, *KNPR* Kernel number per ear, *KWPE* Kernel weight per ear, *RKP* the ratio of kernel production

^b^*Gen* Genotype, *Env* Environment, *Rep (Env)* Replication nested within the environment, *Gen × Env* Genotype × environment interaction

^c^*h*^2^ Broad-sense heritability

^d^*C. I* the confidence interval of broad-sense heritability

^*^Significant at *p* < 0.05 and ^**^ significant at *p* < 0.01

### Identification of QTLs

**Fig. 3:**
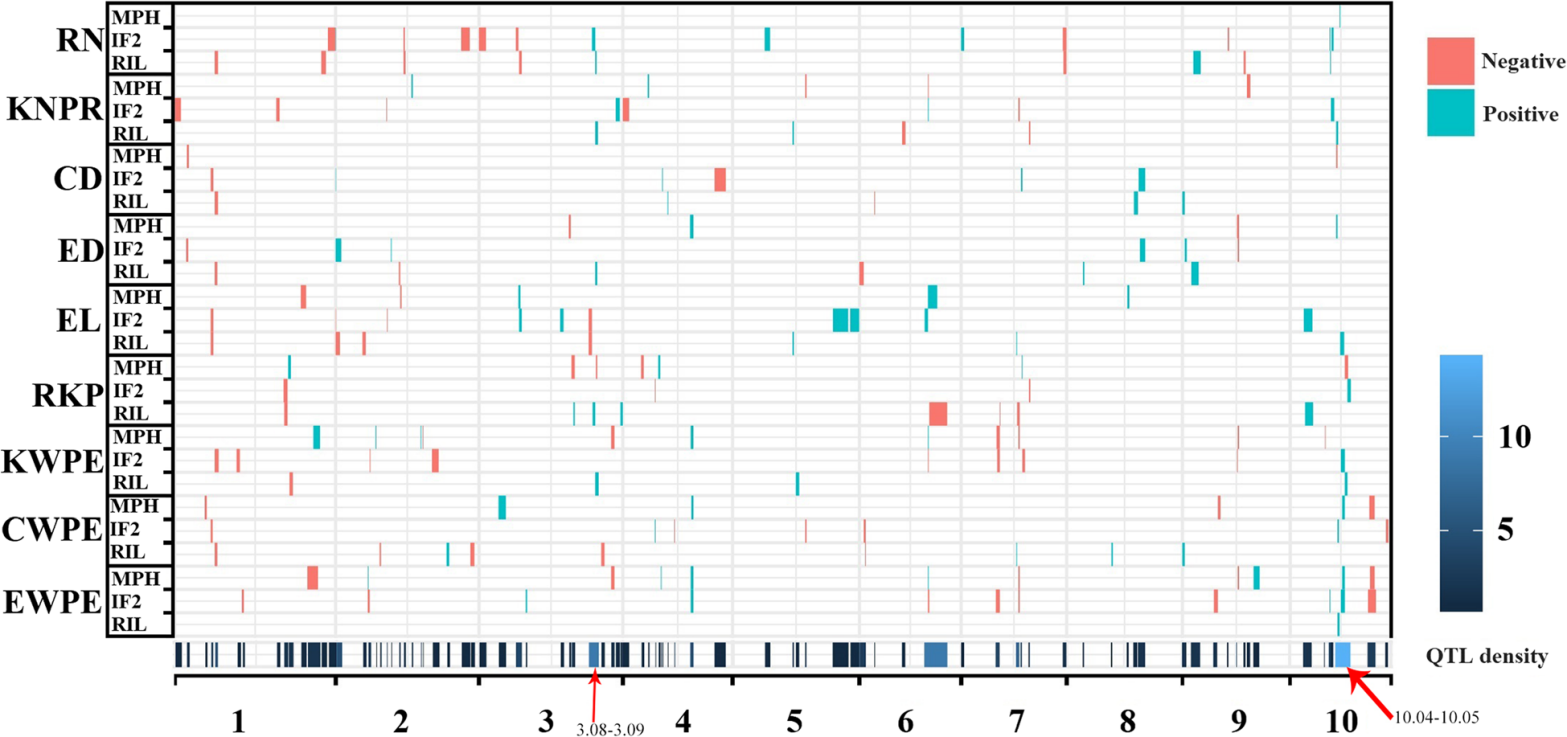
Distribution of QTLs for yield-related traits detected in the RIL and IF_2_ populations and for heterosis. The red rectangles represent QTLs with negative effects (additive in the RIL population, dominant and additive in the IF_2_ population and dominance effects for mid-parent heterosis (MPH)), while the blue rectangles represent positive effects. The width of the rectangles indicates the confidence interval of QTLs. The heatmap of QTL density across ten chromosomes was indicated at the bottom of the figure and below the X-axis. EWPE, ear weight per ear; CWPE, cob weight per ear; EL, ear length; ED, ear diameter; CD, cob diameter; RN, row number; KNPR, kernel number per row; KWPE, kernel weight per ear; RKP, rate of kernel production

In the current study, 156 QTLs were found for averaged yield-related traits and MPH for those traits across all environments (Table 2; Fig. 3). Ninety-six QTLs detected across environments were also identified in at least one environment via single environment analysis (Additional file 6: Table S4). These QTLs were distributed on all chromosomes, although two QTL–concentrated regions were identified at bin 3.08–3.09 and 10.04–10.05. Each QTL explained 0.38 to 33.99% of phenotypic variance (PVE), and over half of QTLs accounted each for less than 5% of PVE (Fig. 2c). The QTL number and the total PVE greatly differed among traits and populations (Table 2; Fig. 2d, e). Nine QTLs were detected simultaneously in the RIL and IF_2_ populations, while 13 QTLs detected in the IF_2_ population and in the MPH dataset co-localized. Only two QTLs found in the RIL population were also positioned in the same regions when QTL analyses were performed for MPH. These results suggested that genetics involved in hybrid performance per se could be, in part, related to that implicated in MPH and it is in agreement with the moderate to high correlation coefficients between hybrid performance per se in the IF_2_ population and the corresponding MPH levels reported for all traits, except for RKP (Additional file 7: Table S5).

**Table 2:** Main features of QTLs for yield-related traits detected in the RILs, the IF_2_ population, and for the mid-parental heterosis (MPH)

QTL	Chr^a^	Physical position^b^	Flanking marker	RIL^c^		IF_2_^c^			MPH^c^		Detected
				A^d^	PVE (%)^e^	A^d^	D^f^	PVE (%)	D^f^	PVE (%)	Environments^g^
*qEWPE1–1*	1	82,577,340/87562343	*PZE-101090729/PZE-101094004*			−0.9	4.5	1.2			C
*qEWPE1–2*	1	263,740,719/285391084	*PZE−101,213,558/SYN22772*						5.3	1.1	C
*qEWPE2*	2	33,839,259/37868398	*PZE-102056295/SYN28948*			-1	7	2.5	5.8	1.1	E3, C
*qEWPE3–1*	3	39,216,459/43866921	*PZE-103041998/SYN18260*			2	3.9	1.3			E4, C
*qEWPE3–2*	3	222,552,568/224622258	*SYN6986/ZM012337–0431*						5.4	0.9	E3, C
*qEWPE4–1*	4	67,494,861/67493572	*PZE-104045414/PZE-104045413*						−14.8	7.4	C
*qEWPE4–2*	4	166,876,428/169511859	*PZE-104090796/PZE-104093153*			2.3	3.4	1.2	3.7	0.9	E3, C
*qEWPE6*	6	140,871,676/137826260	*PZE-106083588/PZE-106080884*			−0.2	5.7	1.7	6.1	1.3	E3, C
*qEWPE7–1*	7	17,478,189/46213540	*PZE-107019133/PZE-107033682*			−2.1	5	1.4			E4, C
*qEWPE7–2*	7	140,421,815/141648137	*PZE-107084740/PZE-107086184*			−1.2	5.6	1.6	5.6	1	C
*qEWPE9–1*	9	20,233,111/24141698	*PZE-109019784/PZE-109023988*			−4.3	2.2	2.2			E3, C
*qEWPE9–2*	9	55,513,159/93380517	*PZE-109037929/PZA03595.2*						1.7	31.6	E3, C
*qEWPE9–3*	9	122,305,846/130925219	*PZE-109075481/PZE-109082403*						5.5	1.1	C
*qEWPE10–1*	10	71,111,717/74412528	*SYN18227/PZE-110038658*			17.7	0.6	32.1			E4, C
*qEWPE10–2*	10	92,787,084/96757754	*PZE-110049371/PZE-110051403*	2.3	6.3						E2, E4, C
*qEWPE10–3*	10	124,526,091/131889929	*PZE-110068110/SYN17753*			0.5	5.9	1.9	5.2	1	E4, C
*qEWPE10–4*	10	142,189,873/146671358	*PZE-110095199/PZE-110104601*			−3.6	1	1.3	0.1	1.1	E3, C
Total PVE (%)					6.3			48.4		48.5	
*qCWPE1–1*	1	29,742,488/29784435	*PZE-101043670/PZE-101043682*						0.004	4.2	C
*qCWPE1–2*	1	38,445,432/39742242	*SYN25114/PZE-101055771*			−0.9	−0.4	2.8			E2, E3, C
*qCWPE1–3*	1	43,974,129/47135598	*SYN13385/SYN37775*	−0.6	5						E3, C
*qCWPE2–1*	2	54,088,944/54766726	*SYN24086/PZE-102074262*	−0.8	8.3						C
*qCWPE2–2*	2	216,546,877/217458575	*PZE-102173306/PZE-102175026*	0.6	4.7						E3, C
*qCWPE2–3*	2	233,109,376/236351458	*PZE-102189664/PZE-102193611*	−0.4	2.9						E3, E4, C
*qCWPE3–1*	3	5,439,550/10520328	*SYN7905/PZE-103018221*						0.02	4.3	E4, C
*qCWPE3–2*	3	215,375,486/216647518	*SYN33394/SYN8639*	−0.4	2.7						C
*qCWPE4–1*	4	41,851,034/56860597	*PZE-104033489/PZE-104041535*			1	−0.3	3.1			E3, C
*qCWPE4–2*	4	142,206,530/143376752	*PZE-104071269/PZE-104072142*			−0.1	0.9	1.1			E2, C
*qCWPE4–3*	4	166,876,428/169511859	*PZE-104090796/PZE-104093153*						0.8	4.3	C
*qCWPE5*	5	167,276,024/168260189	*PZE-105110168/PZE-105111323*			−0.9	0.4	2.1			E4, C
*qCWPE6*	6	6,268,078/24282498	*PUT-163a-94,473,612–4863/PZE-106008406*	− 0.5	3.5	−3	0.4	27.2			E3, C
*qCWPE7*	7	136,331,965/136273480	*PZE-107081442/PZE-107081254*	0.4	2.4						E2, C
*qCWPE8*	8	19,859,565/20691990	*PZE-108020972/PZE-108021854*	0.6	5.4						E3, C
*qCWPE9–1*	9	1,002,880/2145556	*PZE-109000394/PZE-109001604*	0.5	4.2						C
*qCWPE9–2*	9	27,177,667/26944063	*PZE-109027216/PZE-109026940*						0.3	4.8	E4, C
*qCWPE10–1*	10	96,757,754/103084814	*PZE-110051403/PZE-110054264*			0.02	1	1.5			C
*qCWPE10–2*	10	124,526,091/131889929	*PZE-110068110/SYN17753*						1.1	5.5	E4, C
*qCWPE10–3*	10	142,189,873/146124494	*PZE-110095199/PZE-110103156*						−0.04	5.2	C
*qCWPE10–4*	10	148,503,131/149436870	*PZE−110,109,359/PZE-110111130*			-1	−0.2	2.6			C
Total PVE (%)					38.9			40.5		28.3	
*qEL1–1*	1	38,445,432/42018755	*SYN25114/PZE-101058322*	−0.3	6.1	−0.4	− 0.2	6.5			E1, E2, C
*qEL1–2*	1	260,149,117/263740719	*SYN275/PZE-101213558*						0.1	4.2	C
*qEL2–1*	2	7,623,080/7665252	*PZE-102017304/PZE-102017443*	−0.3	4.5	−0.4	0.04	5.5			E1, E2, E3, C
*qEL2–2*	2	27,562,776/31293233	*PZE-102049280/SYN314*	−0.3	5.5						E1, E3, C
*qEL2–3*	2	67,912,086/71361486	*PZE-102082146/PZE-102083803*			−0.2	0.5	6			C
*qEL2–4*	2	148,660,513/151604997	*SYN34894/SYN13599*						0.4	2.2	C
*qEL3–1*	3	24,739,373/29653189	*PZE-103032109/PZE-103036266*			0.3	−0.1	3.5	0.4	2.5	E3, C
*qEL3–2*	3	175,554,472/176784418	*PZE-103115618/PZE-103118170*			0.1	−0.4	3.5			E3, C
*qEL3–3*	3	208,785,867/210358171	*SYN28063/SYN20833*	−0.3	3.9	−0.3	0.1	4.4			E1, C
*qEL5–1*	5	135,189,300/135840044	*PZE-105093385/SYN32229*	0.3	5.1						E2, C
*qEL5–2*	5	205,552,836/208935009	*PZE-105156713/PZE-105165053*			0.3	− 0.1	3.1			E2, C
*qEL5–3*	5	211,173,150/214059839	*SYN9389/SYN14680*			0.3	0.2	3.6			C
*qEL6–1*	6	140,848,748/140871676	*PZE-106083557/PZE-106083588*			0.1	0.3	2.7			C
*qEL6–2*	6	141,080,410/161454721	*PZE-106083873/PZE-106115356*						0.4	2.7	E4, C
*qEL7*	7	136,331,965/136273480	*PZE-107081442/PZE-107081254*	0.3	6.9						E1, E2, C
*qEL8*	8	90,188,031/101409730	*SYN9237/PZE-108056460*						0.9	12	E3, C
*qEL10–1*	10	5,761,296/6537076	*PZE-110007326/PZE-110008811*			0.3	0.2	4.7			E4, C
*qEL10–2*	10	102,323,080/124526091	*PZE-110053918/PZE-110068110*	0.3	5.7						E2, C
Total PVE (%)					37.6			43.6		23.6	
*qED1–1*	1	15,808,888/16371704	*PZE-101026314/PZE-101027182*			−0.4	0.3	0.9			E3, C
*qED1–2*	1	43,974,129/47135598	*SYN13385/SYN37775*	−0.4	3.9						C
*qED2–1*	2	7,623,080/7665252	*PZE-102017304/PZE-102017443*			0.5	−0.1	1.2			E3, E4, C
*qED2–2*	2	107,767,553/113810085	*PZE-102094429/PZE-102097841*			2.2	0.03	18.8			C
*qED2–3*	2	143,268,372/145993939	*SYN11831/PZE-102112161*	−0.4	4.3						E2, E3, C
*qED3–1*	3	184,601,452/184674522	*SYN23237/SYN23245*						0.1	4.5	C
*qED3–2*	3	211,968,759/213654405	*PZE-103161091/PZE-103163529*	0.4	3.4						E2, C
*qED4*	4	166,876,428/169511859	*PZE-104090796/PZE-104093153*						0.4	4.3	C
*qED6*	6	6,268,078/24282498	*PUT-163a-94,473,612–4863/PZE-106008406*	−0.5	6.8						E1, E2, E4, C
*qED8–1*	8	5,605,177/6023577	*PZE-108005561/PZA00058.6*	0.3	2.7						C
*qED8–2*	8	149,193,811/152752292	*PZE-108092173/PZE-108096683*			0.4	0.4	0.8			E4, C
*qED9–1*	9	4,010,398/4838919	*PZE-109003441/SYN22281*			0.5	0.002	1			C
*qED9–2*	9	9,501,951/16179673	*PZE-109008839/PZE-109015923*	0.5	7.6						E1, C
*qED9–3*	9	55,513,159/93381704	*PZE-109037929/PZA03596.1*			−0.4	−0.03	0.8	0.1	5.4	C
*qED10*	10	88,397,317/92787084	*PZE-110047164/PZE-110049371*						0.7	4.5	C
Total PVE (%)					28.6			23.6		18.7	
*qCD1–1*	1	16,371,704/16602399	*PZE-101027182/PZE-101027807*						0.2	6.1	C
*qCD1–2*	1	38,445,432/39742242	*SYN25114/PZE-101055771*			−0.4	−0.04	8.3			E2, E3, C
*qCD1–3*	1	43,974,129/47135598	*SYN13385/SYN37775*	−0.3	4.3						E1, E3, C
*qCD2*	2	7,623,080/7665252	*PZE-102017304/PZE-102017443*			0.3	−0.05	3.7			C
*qCD4–1*	4	75,230,995/75633216	*PZE-104048874/PZE-104049163*			0.4	0.1	6			E3, C
*qCD4–2*	4	99,402,352/84875360	*PZE-104111457/PZE-104053258*	0.4	6.8						E2, C
*qCD4–3*	4	210,243,373/236879938	*PZE-104129635/PZE-104150421*			−0.1	0.5	6			C
*qCD6*	6	60,149,277/70802055	*PZE-106025164/PZE-106029942*	−0.3	6						E3, C
*qCD7*	7	143,113,852/143294688	*SYN35897/PZE-107088218*			0.2	0.2	3.8			C
*qCD8–1*	8	114,826,665/133440733	*SYN21795/PZE-108077809*	0.3	5.2						E4, C
*qCD8–2*	8	130,213,045/149193811	*PZE-108074750/PZE-108092173*			0.3	0.3	4.1			C
*qCD9*	9	1,002,880/2145556	*PZE-109000394/PZE-109001604*	0.3	6.5						E3, C
*qCD10*	10	88,397,317/92787084	*PZE-110047164/PZE-110049371*						0.4	4.4	E4, C
Total PVE (%)					28.8			31.9		10.5	
*qRN1–1*	1	43,974,129/47135598	*SYN13385/SYN37775*	−0.2	4.9						E1, E2, E4 C
*qRN1–2*	1	290,306,827/295888639	*SYN11155/PZE-101251367*	−0.2	3.1	−0.2	− 0.1	1			E1, C
*qRN2–1*	2	7,623,080/7665252	*PZE-102017304/PZE-102017443*			0.2	−0.1	1.4			C
*qRN2–2*	2	153,320,302/163564013	*SYN16390/SYN8399*	−0.2	2.1	−0.3	0.1	1.7			C
*qRN2–3*	2	226,205,828/229917830	*SYN8348/PZE-102186160*			−0.3	0.1	1.8			E4, C
*qRN3–1*	3	1,978,736/3461364	*PZE-103001968/SYN25628*			−0.2	−0.1	1.1			C
*qRN3–2*	3	21,755,425/24739373	*PZE-103029035/PZE-103032109*			−0.3	0.1	1.7			E2, E3, E4, C
*qRN3–3*	3	26,447,512/29653189	*PZE-103033919/PZE-103036266*	−0.3	5.6						E2, E3, C
*qRN3–4*	3	210,358,171/211230808	*SYN20833/PZE-103160158*			0.2	0.1	1.2			E4, C
*qRN3–5*	3	211,968,759/213654405	*PZE-103161091/PZE-103163529*	0.3	6.7						E1, E2, E4, C
*qRN5*	5	7,996,427/17899738	*PZA02029.19/PZE-105032498*			0.3	−0.04	2.2			C
*qRN7–1*	7	775,637/1285778	*PZA01426.1/SYN10723*			0.1	0.2	1.2			E4, C
*qRN7–2*	7	171,743,914/172778289	*PZE-107130514/PZE-107132828*	−0.2	5.1	−0.2	0.003	0.9			E1, E2, E3, E4, C
*qRN9–1*	9	9,501,951/16179673	*PZE-109008839/PZE-109015923*	0.3	6.2						E1, E2, E4, C
*qRN9–2*	9	34,632,675/45578052	*PZB00235.1/PZE-109035290*			−0.4	−0.1	3.3			E2, E4, C
*qRN9–3*	9	97,255,443/98356545	*PZE-109056596/PZE-109057210*	−0.3	9.2						E1, E2, E3, E4, C
*qRN10–1*	10	74,412,528/71111717	*PZE-110038658/SYN18227*			1	0.1	24			E3, C
*qRN10–2*	10	37,810,598/82232426	*PZE-110027837/PZE-110043216*	0.2	2.4	0.4	0.1	3.4			E2, E3, C
*qRN10–3*	10	103,422,421/102323080	*PZE-110054411/PZE-110053918*						−0.1	6.6	E3, C
Total PVE (%)					45.2			44.9		6.6	
*qKNPR1–1*	1	9,434,924/11488064	*SYN14143/PZE-101019726*			−0.8	0.1	4.3			C
*qKNPR1–2*	1	217,114,855/220711263	*PZE-101173330/SYN2411*			−0.7	0.6	5.1			E3, C
*qKNPR2–1*	2	65,134,549/63665753	*PZE-102080979/PZE-102080069*			−0.3	1.3	6.3			E4, C
*qKNPR2–2*	2	177,239,688/178977489	*PZE-102127480/PZE-102129070*						1.1	4.4	E2, C
*qKNPR3–1*	3	211,968,759/213654405	*PZE-103161091/PZE-103163529*	0.9	8						E2, E4, C
*qKNPR3–2*	3	224,622,258/227274031	*ZM012337–0431/PZE-103182712*			0.5	1	5.1			C
*qKNPR4–1*	4	7,155,405/10656171	*PZE-104010113/PZE-104012412*			−0.6	0.6	3.3			C
*qKNPR4–2*	4	35,272,230/35542248	*PZE-104029222/PZE-104029384*						1	5.2	E4, C
*qKNPR5–1*	5	135,840,044/135189300	*SYN32229/PZE-105093385*	0.7	4.7						E4, C
*qKNPR5–2*	5	167,276,024/168260189	*PZE-105110168/PZE-105111323*						−0.1	7.5	C
*qKNPR6–1*	6	105,500,541/106069077	*PZE-106055082/PZE-106055781*	−0.6	3.2						C
*qKNPR6–2*	6	140,871,676/137826260	*PZE-106083588/PZE-106080884*			0.1	1.1	3.9	1.1	4.2	E3, E4, C
*qKNPR7–1*	7	140,421,815/141648137	*PZE-107084740/PZE-107086184*			−0.1	1	3.1			E3, C
*qKNPR7–2*	7	149,763,210/149725744	*PZE-107094398/PZE-107094385*	−0.7	4.6						C
*qKNPR9*	9	101,477,167/105064711	*PZB01899.2/SYN37647*						0.8	3.6	C
*qKNPR10–1*	10	58,452,435/82232426	*PZE-110025994/PZE-110043216*			0.9	0.4	6.1			E4, C
*qKNPR10–2*	10	92,787,084/96757754	*PZE-110049371/PZE-110051403*	0.8	5.5						C
Total PVE (%)					26			37.1		25	
*qKWPE1–1*	1	43,974,129/47135598	*SYN13385/SYN37775*			−1.3	3.6	0.4			C
*qKWPE1–2*	1	73,736,703/77488489	*PZE-101084850/PZE-101087156*			−1.3	3.9	0.4			C
*qKWPE1–3*	1	232,527,769/245035242	*PZE-101187496/PZE-101196838*	−1.9	6.6						E1, C
*qKWPE1–4*	1	263,740,719/285391084	*PZE-101213558/SYN22772*						5.3	0.8	C
*qKWPE2–1*	2	37,868,398/38140456	*SYN28948/PZE-102059924*			−0.4	5.7	0.8			E3, C
*qKWPE2–2*	2	43,372,951/47158891	*PZE-102065424/PZA02450.1*						−16.6	8	E3, C
*qKWPE2–3*	2	188,055,798/190159942	*SYN19995/SYN5428*						1.7	3.2	C
*qKWPE2–4*	2	192,581,253/192700757	*PZE-102145606/PZE-102145703*						1.2	5	C
*qKWPE2–5*	2	201,971,890/209300839	*PZE-102154251/SYN7501*			−3.3	1.7	0.5			C
*qKWPE3–1*	3	211,968,759/213654405	*PZE-103161091/PZE-103163529*	1.7	5.5						E1, E2, C
*qKWPE3–2*	3	222,552,568/224622258	*SYN6986/ZM012337–0431*						3.9	0.5	C
*qKWPE4*	4	166,876,428/169511859	*PZE-104090796/PZE-104093153*						2.5	0.4	C
*qKWPE6–1*	6	140,871,676/137826260	*PZE-106083588/PZE-106080884*			−0.4	4.9	0.6	4.7	0.7	E1, E3, E4, C
*qKWPE6–2*	6	142,423,964/152749874	*PZE-105096701/PZE-105101729*	1.4	3.7						C
*qKWPE7–1*	7	17,478,189/46213540	*PZE-107019133/PZE-107033682*			−1.8	5.8	0.8	5	0.8	E2, E4, C
*qKWPE7–2*	7	140,421,815/141648137	*PZE-107084740/PZE-107086184*						3.8	0.5	C
*qKWPE7–3*	7	144,000,432/144753122	*PZE-107088998/PZE-107089819*			−1.9	3.9	0.4			E3, C
*qKWPE9*	9	55,513,159/93380517	*PZE-109037929/PZA03595.2*			−26.2	1.8	34	1.5	18.3	E2, E4, C
*qKWPE10–1*	10	23,214,551/25558345	*PZE-110019199/PZE-110020162*						−21.3	13.4	C
*qKWPE10–2*	10	124,526,091/131889929	*PZE-110068110/SYN17753*			2.4	3.9	0.7			E4, C
*qKWPE10–3*	10	131,104,195/134219127	*PZE-110074914/PZE-110079903*	1.6	4.4						C
Total PVE (%)					20.1			38.8		51.6	
*qRKP1–1*	1	223,825,525/229541701	*SYN31271/PZE-101184757*	−0.01	4.7	−0.004	0.004	3.6			E1, E3, C
*qRKP1–2*	1	232,527,769/245035242	*PZE-101187496/PZE-101196838*						− 0.003	7.7	C
*qRKP3–1*	3	184,674,522/191932135	*SYN23245/PZE-103136534*	0.01	4.8				−0.003	5.7	E1, E2, E4, C
*qRKP3–2*	3	211,230,808/213654405	*PZE-103160158/PZE-103163529*	0.01	4.3				0.01	6.7	E2, E3, E4, C
*qRKP3–3*	3	227,274,031/227827542	*PZE-103182712/PZE-103183391*	0.01	3.6						C
*qRKP4–1*	4	25,639,678/26730656	*PZE-104022764/PZE-104023674*						0.014	4.1	C
*qRKP4–2*	4	41,851,034/56860597	*PZE-104033489/PZE-104041535*			−0.01	0.001	8.4			E2, E3, E4, C
*qRKP4–3*	4	78,343,158/82013799	*PZE-104050391/PZE-104051877*						0.001	10.4	E2, E4, C
*qRKP6*	6	141,080,410/161454721	*PZE-106083873/PZE-106115356*	−0.01	4.8						E4, C
*qRKP7–1*	7	39,125,424/46213540	*PZE-107030398/PZE-107033682*	−0.01	4.1						C
*qRKP7–2*	7	140,421,815/141648137	*PZE-107084740/PZE-107086184*	−0.02	6.8						E4, C
*qRKP7–3*	7	143,113,852/143294688	*SYN35897/PZE-107088218*						0.003	3.5	C
*qRKP7–4*	7	149,763,210/149725744	*PZE-107094398/PZE-107094385*			−0.01	0.002	4.6			E4, C
*qRKP10–1*	10	5,761,296/6537076	*PZE-110007326/PZE-110008811*	0.01	5.4						C
*qRKP10–2*	10	131,104,195/134219127	*PZE-110074914/PZE-110079903*			0.01	0.003	4.6	−0.002	4.7	C
Total PVE (%)					38.4			21.2		42.7	C

^a^*Chr* Chromosome

^b^The version 2 of the Maize B73 RefGen

^c^*RIL* the RILs, *IF*_*2*_ the IF_2_ population, *MPH* the MPH data set derived from the RILs and IF_2_ population

^d^*A* additive effect, Negative additive values indicate that the allele for increasing trait value is contributed by the parent 08–641, Positive additive values indicate that the allele for increasing trait value is contributed by another parent YE478

^e^*PVE* phenotypic variance explained by QTL

^f^*D* dominance effect

^g^*E1* 2014Jinghong, *E2* 2015Jinghong, *E3* 2016Chongzhou, *E4* 2016Jinghong, *C* combined analysis across all environments

### Dominance and overdominance effects play crucial roles in heterosis for yield-related traits

**Fig. 4:**
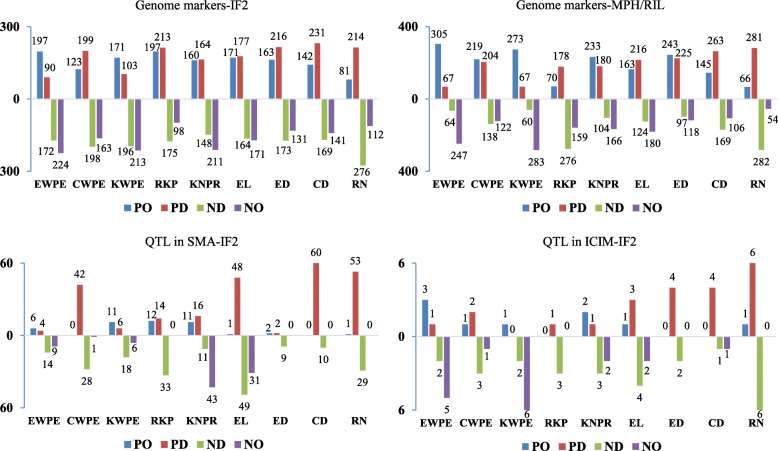
Distribution of markers in the whole genome and significant loci for yield-related traits with dominance and over-dominance effect. The ratio *D/A* was calculated in two ways: both additive and dominance genetic effects (*A* and *D*) were estimated from the IF_2_ population (IF_2_) or additive effects (*A*) were estimated from RILs and dominance effects (*D*) from the MPH dataset (MPH/RIL). SMA, single-marker analysis; ICIM, inclusive composite interval mapping. PO, PD, ND, and NO, indicate positive overdominance, positive dominance, negative dominance, and negative overdominance effects, respectively. EWPE, ear weight per ear; CWPE, cob weight per ear; EL, ear length; ED, ear diameter; CD, cob diameter; RN, row number; KNPR, kernel number per row; KWPE, kernel weight per ear; RKP, rate of kernel production

The contribution of dominance and overdominance to the heterosis phenomena was assessed by the (|D/A|) ratio for each locus [34, 39] using single-marker analysis (SMA) and inclusive composite interval mapping (ICIM) (Fig. 4). Overdominance was detected for over 60% of genomic markers for EWPE and KWPE in both the IF_2_ and MPH/RIL datasets, more than 50% for KNPR and EL, over 40% for CWPE and ED, over 30% for CD and RKP, and over 15% for RN. Based on SMA, the number of significant markers in the IF_2_ population showing overdominance accounted for 66.6% for KNPR, more than 40% for EWPE and KWPE, more than 15% for EL, RKP, and ED, and less than 2% for CWPE, CD, and RN, respectively. Additionally, approximately 78% of QTLs for KWPE exhibited overdominance, as did 72.7% of QTLs for EWPE, 50% of QTLs for KNPR, and less than 30% of QTLs for the remaining traits. Among traits, we also found a strong association between the proportion of QTLs with overdominance and the ratio of MPH/mid-parent for F_1_ (*r*^2^ = 0.951^***^) or the MPH level across hybrids from the IF_2_ population (*r*^2^ = 0.966^***^). We found, in general, significant but low correlations between performance per se or MPH of individuals in the IF_2_ population and their percentages of heterozygous marker loci (Additional file 8: Table S6).

### Analysis of EPI and QEI

We estimated the total PVE by the epistatic loci in the whole-genome search using ICIM-EPI (Additional file 2: Figure S2). In the IF_2_ population_,_ epistatic loci had higher PVE than single-locus QTLs for CWPE (50% vs 41%), ED (24% vs 20%), CD (56% vs 32%), and KNPR (53% vs 37%). In the MPH dataset, the total PVE by epistatic loci was greater than by single-locus QTLs for all traits except for EWPE (20% vs 49%), CWPE (23% vs 28%), KWPE (43% vs 52%), and RKP (16% vs 43%), indicating that EPI had a greater impact on these traits than single-locus QTLs, regardless of the statistical significance. The current findings also suggest that the cumulative effects resulting from combining the epistatic loci and single-locus QTLs explained a large proportion of PVE. Meanwhile, based on mixed-model-based composite interval mapping (MCIM) with the full QTL model, 28 pairs of marker intervals showed significant epistatic interaction effects; 13 additive by additive (AA) interactions were detected in the RIL population, 10 epistatic interactions in the IF_2_ population, and five digenic interactions in the MPH dataset (Additional file 9: Table S7). We observed 23 pairs of significant AA interactions, three for additive by dominance (AD) interactions, seven for dominance by additive (DA) interactions, and six for dominance by dominance (DD) interactions. Each marker interval interacted with one to five loci. In addition, a total of nine loci related to four traits exhibited significant additive × environment interactions in the RIL and IF_2_ populations, seven in the RILs, and two in the IF_2_ population; only one locus, *qKWPE2–2*, for the MPH level for KWPE showed a significant dominance × environment interaction in the IF_2_ population (Additional file 10: Table S8). Many of these marker intervals involved in epistasis and QEI also contained QTLs with main effects on yield-related traits or MPH. For instance, the interval *PZE-101184757/SYN25826* was involved in three significant AA interactions and contains *qRKP1–1*.

### QTL co-localization across traits

Overlapping QTLs for different traits were assigned to the same QTL cluster. In total, 95 QTLs were grouped into 33 QTL clusters distributed across all chromosomes (Table 3). Each QTL cluster contained QTLs for two to six traits. The cluster QC3–3 harboring six QTLs affected the means of EL, RN, ED, KNPR, RKP, and KWPE, as well as the MPH for RKP. QC10–3, within a 5 cM region on chromosome 10, had a pleiotropic effect on EWPE, KNPR, CWPE, and the MPH level of ED and CD. As expected from the moderate to high correlation between hybrid performance per se and MPH, some loci associated with yield-related traits and MPH gathered together. Nearly 60% of QTL clusters were associated with two traits. Some of these clusters were specific for a determined population or for MPH. For example, clusters QC1–5 (bin 1.09–1.11) affected mid-parent heterosis for EWPE and KWPE. Contrarily, many other clusters comprised QTLs for yield-related traits and heterosis for those traits. Cluster QC1–4 (bin 1.08) affected KWPE and MPH for RKP. Genomic region 3.09 affected KNPR, RKP, and the MPH level of KWPE and EWPE. Interestingly, some of these clusters co-located in the interval of loci showing epistasis and QTL × environment interactions. For instance, QC7–1 (bin 7.02) had a pleiotropic effect on RKP, EWPE, KNPR, KWPE, and the MPH level for KWPE. This locus interacted with other loci exhibiting epistatic effects for RN in the RILs and for KWPE in the IF_2_ population. This hotspot also interacted with E1 (2014JH) for RKP in the RILs. These results further confirmed the complex genetic architecture of yield-related traits.

**Table 3:** QTL clusters for yield-related traits and for heterosis for those traits detected in the RILand IF_2_ populations

QTL Cluster^a^	Bin^b^	Interval (cM)	Flanking marker	No. of QTL	Traits influenced^c^	References^d^
QC1–1	1.02	17.5–21.5	*PZE-101026314/PZE-101027807*	2	ED (I), CD (M)	
QC1–2	1.03	54.5–58.5	*SYN25114/PZE-101058322*	3	EL (I, R), CD (I), CWPE (I)	67, 35
QC1–3	1.03	60.5–66.5	*SYN13385/SYN37775*	5	ED (R), CD (R), CWPE R), RN (R), KWPE (I)	67, 71
QC1–4	1.08	171.5–179.5	*PZE-101187496/PZE-101196838*	2	RKP (M), KWPE (R)	67, 35
QC1–5	1.09–1.11	200.5–219.5	*PZE-101213558/SYN22772*	2	EWPE (M), KWPE (M)	31, 34, 35
QC2–1	2.02	0–8.5	*PZE-102017304/SYN7604*	4	EL (I, R), CD (I), ED (I), RN (I)	35
QC2–2	2.04	48.5–52.5	*PZE-102056295/PZE-102059924*	2	EWPE (M, I), KWPE (M)	29, 35, 71
QC3–1	3.04	50.5–64.5	*SYN28119/PZE-103036266*	2	RN (R, I), EL (I, M)	67
QC3–2	3.06–3.07	135.5–144.5	*SYN23237/PZE-103136534*	2	ED (M), RKP (R, M)	
QC3–3	3.08	165.5–180.5	*SYN28063/PZE-103163529*	6	EL (I, R), RN (I, R), RKP (R, M), ED (R), KNPR (R), KWPE (R)	35
QC3–4	3.09	189.5–217	*PZE-103171163/PZE-103183391*	4	KNPR (I), KWPE (M), EWPE (M), RKP (R)	34, 37, 71
QC4–1	4.05	48.5–49.5	*PZE-104033489/PZE-104041535*	2	CWPE (I), RKP (I)	
QC4–2	4.06	101.5–106.5	*PZE-104090796/PZE-104093153*	4	EWPE (I, M), CWPE (M), ED (M), KWPE (M)	
QC5–1	5.04	90.5–92.5	*PZE-105093385/SYN32229*	2	EL (R), KNPR (R)	67
QC5–2	5.04	109.5–111.5	*PZE-105110168/PZE-105111323*	2	CWPE (I), KNPR (M)	71
QC6–1	6.00–6.01	0–9.5	*PUT-163a-94,473,612–4863/PZE-106008406*	2	ED (R), CWPE (R, I)	71
QC6–2	6.05	98.5–105.5	*PZE-106083557/PZE-106080884*	4	EL (I), EWPE (I, M), KNPR (I, M), KWPE (I, M)	
QC6–3	6.05–6.07	103.5–132.5	*PZE-106083873/PZE-106115356*	3	EL (M), RKP (R), KWPE (R)	71
QC7–1	7.02	52.5–59.5	*PZE-107019133/PZE-107033682*	4	RKP (R), EWPE (I), KWPE (I, M), KNPR (R)	35, 71
QC7–2	7.03	83.5–84.5	*PZE-107081442/PZE-107081254*	2	EL (R), CWPE (R)	34
QC7–3	7.03	84.5–88.5	*PZE-107084740/PZE-107086184*	4	KNPR (I), EWPE (M, I), RKP (R), KWPE (M)	29
QC7–4	7.03	90.5–92.5	*SYN35897/PZE-107088218*	2	CD (I), RKP (M)	
QC7–5	7.03	102.5–104.5	*PZE-107094398/PZE-107094385*	2	KNPR (R), RKP (I)	35, 71
QC8	8.05–8.06	108.5–118.5	*PZE-108074750/PZE-108096683*	2	CD (I), ED (I)	71
QC9–1	9.00–9.01	0–3.5	*PZE-109000394/PZE-109001604*	2	CWPE (R), CD (R)	
QC9–2	9.01–9.02	13.5–27.5	*PZE-109008839/PZE-109015923*	2	ED (R), RN (R)	35, 60
QC9–3	9.03	82.5–85.5	*PZE-109037929//PZA03596.1*	3	KWPE (I, M), EWPE (M), ED (I, M)	71
QC10–1	10.02	20.5–34.5	*PZE-110007326/PZE-110008811*	2	EL (I), RKP (R)	29
QC10–2	10.03	59.5–66.5	*SYN18227/PZE-110043216*	3	EWPE (I), KNPR (I), RN (R, I)	60
QC10–3	10.04	69.5–74.5	*PZE-110047164/PZE-110054264*	5	ED (M), CD (M), EWPE (R), KNPR (R), CWPE (I)	29
QC10–4	10.04–10.05	74.5–82.5	*PZE-110054411/SYN17753*	5	RN (M), EL (R), EWPE (I, M), KWPE (I), CWPE (M)	29, 71
QC10–5	10.05	82.5–91.5	*PZE-110074914/PZE-110079903*	2	KWPE (R), RKP (I, M)	35, 71
QC10–6	10.06–10.07	117.5–129.5	*PZE-110095199/PZE-110104601*	2	EWPE (I, M), CWPE (M)	71

^a^The nomination of QTL cluster is made as follows: a “QC” standing for the abbreviation of QTL cluster, a number for chromosome and another for the physical order

^b^The genetic region included the position of QTL. (http://www.maizegdb.org/)

^c^R, the RILs; I, the IF_2_ population; M, mid-parent heterosis

^d^29, Frascaroli et al. 2007; 31, Tang et al. 2010; 67, Guo et al. 2011; 34, Guo et al. 2014; 35, Wang et al. 2016; 60, Liu et al. 2016; 37, Li et al. 2017; 71, Chen et al. 2017

## Discussion

### Heterotic loci with dominance and epistasis and the complex genetic network of “crosstalk” contribute to grain yield

Previous studies [29, 31, 34, 40, 41] proposed that better-parent heterosis was yield-related loci displaying dominance, overdominance, and/or epistasis. In the present study, over half of QTLs exhibited overdominance for KWPE, EWPE, and KNPR, which is similar to previous results reported by Frascaroli et al. [29] for overdominant loci for grain yield and kernel number per plant and by Guo et al. [34] for single markers and QTLs for grain yield. Such a high average degree of overdominance for grain yield and kernel number per plant could be, in part, explained by linkage in the repulsion of loci of the dominance type in mapping populations [29]. In accordance with this, greater number of genome markers with overdominance for EL and ED was previously observed in the F_2:3_ population, from which the current RIL population has been derived [42], than in the present mapping populations. The degree of dominance for the traits was well in accordance with the level of F_1_ superiority over mid-parent performance and the type of inheritance of different traits varied among those traits, which was consistent with other studies [29, 33, 34, 37, 43]. High correlation coefficients were observed between the proportion of QTLs displaying overdominance and the MPH levels across different traits. For example, the F_1_ did not differ from the inbred parent lines 08–641 and Ye478 for RN and CD, and accordingly QTLs, such as qRN1–2 and qRN7–2 displaying similar additive effects, detected for those traits showed low or no significant dominance effects and were mainly detected in RIL and IF_2_ populations [34]. The QTLs for EWPE, KNPR, and KWPE in IF_2_ population and the MPH dataset displayed high dominance effects. In general, similar dominance effects were found in the IF_2_ population and in the MPH dataset, but epistatic effects could interfere in the estimation of dominance effects and cause discrepancies between results obtained in different populations [44]. Discrepancies between additive effects estimated in the RIL and IF_2_ populations were even more conspicuous. For example, additive effects estimations for *qCWPE6* were different when obtained from RIL or IF_2_ evaluations. These findings demonstrated that the loci displaying dominance contributed to yield-related traits and heterosis for those traits, although overdominance had an outstanding contribution to heterosis for EWPE, KWPE, and KNPR, which presented the highest values for MPH.

The level of whole marker loci with heterozygosity and phenotypic variations (hybrid performance per se or MPH) were weakly correlated, whereas Frascaroli et al. [29] and Larièpe et al. [33] demonstrated moderate-to-high correlations between them. Such low correlations indicated that the overall heterozygosity contributed little to hybrid performance per se or heterosis [45, 46]. Huang et al. [46] demonstrated that phenotypic variations for yield-related traits are highly correlated with the number of accumulated superior gene alleles. In contrast, numerous epistatic effects and QTL × environment interactions for yield-related traits have been addressed in the literature [29, 31, 34, 37, 47–50]. The total PVE values by single-locus QTLs and epistasis together approach the high heritability for these yield-related traits [34], indicating the cumulative effects of different genetic effects [34, 41]. The QTL model, which was used to estimate the loci showing additive, dominance, and EPI effects, identified 28 pairs of significant loci with epistatic effects and 10 loci with QTL × environment interaction in the two mapping populations. Of those, only five pairs of epistatic loci were observed for MPH. In addition, many of these loci showing interactions did not co-map with putative QTLs. Similar features were also reported by Yang et al. [51]. EPI and QEI are also involved in the complex genetic web for grain yield via “cross-talk” among loci [45, 47, 48, 52]. Collectively, the cumulative effects of various genetic effects discussed above seem to explain the genetic mechanism of grain yield and better-parent performance [34, 41].

### QTL hotspots regulate the architecture of yield-related traits and heterosis

Over 60% of QTLs gathered together and were integrated into 33 clusters distributed on 10 chromosomes. We found a good consistency for QTL clusters between our study and former studies (Table 3). Thirteen pleiotropic regions in the present study were consistently mapped to the clusters in all chromosomes, except in chromosome 4, that were defined by Chen et al. [53] in their meta-analysis of yield-related QTLs. Notably, many of these clusters related to MPH co-located with QTLs found in previous studies [29, 31, 34, 35] for better parent performance (Table 3). Cluster QC1–2 associated with EL, CD, and CWPE overlapped with the hotspot region for ear weight, grain weight, and KNPR between 38 and 42 Mb on chromosome 1, which was highlighted by Guo et al. [49], and with a genomic region containing QTLs for heterosis for grain yield, ear weight, kernel weight, and ear length reported by Wang et al. [35]. The heterosis locus QC1–5 (1.09–1.11) for EWPE and KWPE co-located with QTLs for heterosis for EWPE and KRP presented by Wang et al. [35]; with a heterosis locus for grain yield at approximately 271 Mbp found by Tang et al. [31] using the heterotic pattern Mixed × Flint and by Larièpe et al. [33] using the pattern European flint × Dent; and with QTLs for 100-kernel weight and grain yield in an IF_2_ population reported by Guo et al. [34]. The genomic region 2.04 (QC2–2) controlling EWPE and the MPH level for EWPE and KWPE co-located with a QTL involved in heterosis for EL, RN, KWPE, KRP, EWPE, and grain yield by Wang et al. [35], and it was nearby the region implicated in heterosis for grain yield in the Reid × Lancaster pattern reported by Frascaroli et al. [29] and the meta-QTL for grain yield, ear-related traits, and kernel-related traits highlighted by Chen et al. [53]. Cluster QC3–3, being particularly interesting in the current study because of its association with heterosis for several traits, overlapped with a QTL hotspot for heterosis for EL, EWPE, RN, KRP, KWPE, and grain yield located between 209 and 213 Mbp on chromosome 3 reported by Wang et al. [35]. Genomic region 3.09 (QC3–4), related to KNPR and RKP and the MPH level for KWPE and EWPE overlapped with the environmentally stable region for heterosis for KNPR, ear weight, and kernel weight between 218 and 228 Mbp on chromosome 3 reported by Li et al. [37] in the heterotic pattern Reid × TSPT. Another hotspot region, QC10–3, related to five traits co-mapped with a heterotic locus that affects grain yield and kernel number presented by Frascaroli et al. [29]. In addition, genomic regions 2.04, 7.02, and 9.03, associated with heterosis for KWPE, contain QTLs for grain yield heterosis in the Reid × Lancaster heterotic pattern published by Schön et al. [32]. The cluster QC6–3 for RKP, KWPE, and the MPH level for EL overlapped with the heterotic loci for grain yield found by Samayoa et al. [36] in the population derived from an American dent × European flint cross. These results suggested the consistency of hotspot regions across different heterotic patterns, which may be used for genetic improvement of grain yield in the future.

In contrast, hybrid performance per se for yield-related traits and the MPH level for those traits were moderately to highly correlated and 13 QTLs found in the IF_2_ population co-localized with QTLs for MPH. These findings indicated the likely common genetic basis between grain yield and its MPH level. The results also partly supported that heterosis loci for grain yield are not independent from loci for grain yield, thus agreeing with Guo et al. [34] and refuting the conclusions by Tang et al. [31] who proposed independence between genomics involved in performance per se and heterosis.

### Genetic basis for grain yield and its components in different populations from the same parents

Our study revealed that QTLs found in different populations derived from the same cross are not always consistent with previous studies [34, 54–56]. Among the 156 detected QTLs for yield-related traits and heterosis, only one QTL, *qRN10–2*, was previously found across F_2:3_ families derived from the cross 08–641 × YE478 [60]. In addition, only nine QTLs with |D/A| < 1 were simultaneously detected in the RILs and the IF_2_ population. Lack of congruency for QTLs found in populations derived from the same cross but with different homozygous levels could be due to the different genetic effects acting in each population, but also to the different environments used for evaluations and biases caused by population sampling [29].

## Conclusions

Heterosis for yield-related traits in maize is one of the main issues for maize breeders, and investigation and assessment of heterotic loci involved in specific heterotic patterns are of particular interest. The inheritance of yield-related traits and MPH varied among different traits. A large proportion of the loci with dominance effect had a greater effect on most traits compared with loci with other genetic effects, whereas overdominance also contributed greatly to MPH for KNPR, EWPE, and KWPE in the current heterotic pattern. As QTL hotspots at 1.09–1.11, 2.04, 3.08–3.09, and 10.04–10.05 encompass genomic regions where other authors have found QTLs for heterosis across different heterotic patterns, markers in those regions could be used in marker-assisted selection programs for increased yield.

## Methods

### Plant material and field experiments

The mapping populations used in this study consisted of 301 RILs derived from a cross between the maize inbred lines 08–641 and Ye478. The elite inbred 08–641 comes from the Maize Research Institute of Sichuan Agricultural University, and Ye478 was provided by the Project of National Major Basic Dairy Research “973” Plan, which is developed by the Laizhou Academy of Agricultural Sciences [17]. The parental line 08–641 was crossed with Ye478 in the winter of 2010 to produce a set of RILs (F_8_) by using the single seed descent method. The 301 RILs and the two parental lines were evaluated in four environments. Three environments were located at the Xishuangbanna maize breeding base of the Maize Research Institute of Sichuan Agricultural University, Jinghong (JH 21°95 N, 100°76E; average daytime temperature 18.6–21.9 °C; average rainfall per year 1200–1700 mm; tropical humid monsoon climate), in Yunnan province, in March 2014 (JH2014), March 2015 (JH2015), and March 2016 (JH2016), and one environment was located at the Modern Agriculture Research and Development Center of Sichuan Agricultural University, Chongzhou (CZ 30°33′N, 103°38′E; average daytime temperature 15.9 °C; average rainfall per year 1012.4 mm; sub-tropical humid monsoon climate), in Sichuan province, April 2016 (CZ2016). An IF_2_ population was developed with 320 RILs following the procedure of Hua et al. [57] and Tang et al. [31] The 320 RILs were randomly divided into two groups of 160 RILs. Then, the lines of both groups each RIL was only involved in a single cross, yielding 160 F_1_ crosses. We repeated the procedure mentioned above twice and constructed 320 crosses that constituted the IF_2_ population. After genotyping the RILs, those with heterozygosity above 20% and their respective crosses were removed. After filtering the RILs, the IF_2_ population consisted of 298 crosses that were evaluated under three environments (2015JH, 2016JH, and 2016CZ). In each environment, each trial was performed following a randomized complete block design with two repetitions. Plant density was 57,000 plants per ha. Each plot consisted of a single row. Each row with 14 plants was 3 m long, with 0.80 m space between rows. The trials with two mapping populations were adjacent and occupied a uniform and square parcel. Field management was in accordance with local practices.

### Phenotypic measurements and analysis

In each plot, 10 well-pollinated ears were randomly chosen from all ears harvested. The nine yield-related traits measured (Additional file 3: Table S1) were: EL (cm), ED (mm), CD (mm), RN (count), KNPR, EWPE (g), CWPE (g), KWPE (g), and RKP. Means for each replication were used for further analyses. The distribution of the phenotypic traits and the Pearson’s correlation, using PROC CORR from the statistical software package SPSS 17.0 (SPSS, Inc., Chicago, IL, USA), were conducted with the means of the phenotypic traits in the 301 RILs and 298 hybrids of the IF_2_ population across all environments. A hierarchical cluster analysis (“hclust”) was carried out for phenotypic traits, based on the standardized data from the RILs [58, 59]. Combined analyses of variance of the RILs and the IF_2_ population were computed for each trait using the GLM procedure in SPSS 17.0 (SPSS, Inc., Chicago, IL, USA) with genotype as a fixed effect and replication and environment as random effects. Broad-sense heritability (*h*^2^) for the RILs and IF_2_ population was computed on an entry mean basis as described by Hallauer and Miranda [60]:

1 $$ {h}^2={\sigma}_g^2/\left({\sigma}_g^2+\frac{\sigma_{ge}^2}{n}+\frac{\sigma^2}{nb}\right), $$

where ${\sigma}_g^2$ represents the genetic variance, ${\sigma}_{ge}^2$ represents the genotype × environment interaction variance, *σ*^2^ represents the error variance, *b* represents the number of replications, and *n* is the number of environments. The 90% confidence interval of heritability (*h*^2^) was determined according to Knapp et al. [61]. Each phenotypic trait of the 301 RILs and each trait of the IF_2_ population were analyzed following two mixed models fitted by restricted maximum likelihood:

2 $$ {Y}_{mk}=\mu +{G}_m+{R}_k+{\varepsilon}_{mk}, $$

3 $$ {\mathrm{Y}}_{\mathrm{mik}}=\upmu +{G}_m+{GE}_{mi}+{E}_i+{R}_k+{\varepsilon}_{mi k}, $$

where *Y*_*mk*_ is the phenotypic value of genotype m in replication k; *Y*_*mik*_ indicates the phenotypic value of genotype *m* in environment *i* and replication *k*; *μ* is the overall mean of the RIL population or the IF_2_ population; *G*_*m*_ is the random effect of genotype *m*; *GE*_*mi*_ is the random effect of the interaction between genotype *m* and environment *i*; *E*_*i*_ is the random effect of environment *i*; *R*_*k*_ is the random effect of replication *k*; and *ε*_*mk*_ and *ε*_*mik*_ denote the random experimental error. The best linear unbiased predictor values (BLUPs) with Eqs. (2) and (3) were used for single and combined environment analysis, respectively. The adjusted mean BLUP values of each RIL across four environments and each hybrid across three environments were used for QTL mapping. The analyses were conducted with the R program for statistical computing [59, 62]. Besides, MPH was estimated as [40]:

4 $$ {\mathrm{MPH}}_{12}={\mathrm{F}}_{12}-\left({\mathrm{P}}_1+{\mathrm{P}}_2\right)/2 $$

where F_12_ is the genotypic value of the F_12_ individual from the IF_2_ population, and (P_1_ + P_2_)/2 is the average of BLUP values of the corresponding parents (P_1_ and P_2_ from the RIL population) estimated from the RIL evaluation. The genotypic dataset for MPH depends on dominance and epistatic effects, but epistatic effects were not included and an additive-dominance model was assumed for the IF_2_ population [31, 34].

### Molecular linkage map construction

Following the modified CTAB protocol [63], DNA was isolated from 7-day-old seedling leaves of the 301 RILs and the parents grown in shade (10 plants per RIL as a bulk). The oligonucleotide pool assay consisted of 3072 well-distributed, high-quality single nucleotide polymorphisms (SNPs) from all 10 maize chromosomes that were selected from 56,110 SNPs in 513 maize inbred lines developed by the National Maize Improvement Center of China using Illumina GoldenGate technology. The protocol for genotyping SNPs using an Illumina BeadStation 500 G (Illumina, San Diego, CA, USA) was conducted as described by Fan et al. [64]. Marker data from the RILs were filtered for heterozygous data points (< 20%), missing data points (< 20%), and segregation distortion (in accordance with the expected Mendelian segregation ratio of 1:1). A total of 683 SNPs selected for their uniform distribution throughout all 10 maize chromosomes were used to construct the linkage map with a total genetic length of 1786.1 cM and an average interval distance of 2.61 cM. The genotypes of each cross from the IF_2_ population were deduced from the marker genotypes of their RIL parents, with QTL mapping for the IF_2_ population and the MPH dataset performed using the molecular linkage map of the RIL population [31, 57]. The genetic map for the RILs and IF_2_ population was developed using MapDisto 1.7.5 [65]. The Kosambi mapping function was used for converting recombination frequencies to genetic distances [66]. The average proportion of homozygous loci for YE478 variants, homozygous loci for 08–641 variants, and heterozygous loci in each individual of the IF_2_ population was 25.2, 24.7, and 50.1%, respectively, according to the expected ratio in an F_2_ population.

### QTL mapping

QTL mapping for each trait in the RIL and IF_2_ populations and for MPH using both populations was performed using QTL ICIMapping software [67] with ICIM [68, 69]. For QTL detection on ICIM-ADD, the largest *p*-value for entering variables in stepwise regression of residuals on marker variables, PIN was set to 0.001. The largest *p*-value for removing variables is assumed to be two times the PIN. The threshold logarithm of the odds (LOD) score to declare significant QTL was calculated using the 1000 permutation test at Type I error of 0.05 with the step size of 1 cM was 2.5 for the RILs, IF_2_ population, and the MPH dataset since very minor differences were observed in LOD score among them. Under the additive-dominance model assumed, only QTLs with dominant effect were detected for MPH [34]. We also performed single marker analysis (SMA) to detect QTLs with the same empirical threshold LOD mentioned previously, and the degree of dominance was calculated as a ratio of dominance to additive effects (|D/A|) for each QTL [34]. Loci with |D/A| values greater than 1.26 displayed overdominance; otherwise, the QTL was treated as dominance type [39]. The QTLs were nominated as follows: a lowercase letter “q” standing for QTL, the trait abbreviation, one number standing for the chromosome and another for the physical order or the QTL within the chromosome. The QTLs for different traits detected in overlapping or adjacent intervals were regarded as a QTL cluster. The graphical presentation of linkage maps and QTLs were generated with the R program [59]. To scan for significant marker interval interactions, epistasis interaction (EPI, digenic interactions; AA, AD, DA, and DD) was identified with the step in scanning of 5 cM and PIN = 0.0001 on ICIM-EPI. An empirical threshold LOD for digenic epistasis interaction was set at 5. Meanwhile, the QTL Network program 2.1 based on MCIM [70–72] was also used to identify QTL with AA effects in the RILs; AA, AD, DA, and DD effects in the IF_2_ population using combined data across all environments and the MPH dataset using the average data across environments. QTL × environment interaction effects (QEI) in the RIL and IF_2_ population were estimated for yield-related traits via combined analysis across all environments. The testing window and filtration window size was set at 10 cM with a walk speed set to 2 cM for one-dimensional genome scans. The F-statistic and the critical F-value were estimated with the help of 1000-permutations test [73] with *p* < 0.05 as experiment-wise significance level for candidate interval selection. The final full QTL model for each trait incorporated significant additive, dominance, and epistatic effects, as well as their interactions with environments. Bonferroni correction was used to compute the comparison-wise significance threshold assuming an experiment-wise error < 0.05 [74]. We also calculated the percentage of heterozygous loci for each RIL analyzed the correlation relationships between phenotypic performance and heterozygosity, and conducted regression analyses of phenotypic performance on heterozygosity [59].

## Additional files

Additional file 1: **Figure S1.** The performance of parental inbreds of the RIL population (YE478 and 08–641) and the hybrid F1 across four environments. The Duncan multiple range test was used for the comparison of means. Genotypes with lower-case letters were significantly different at the 0.05 probability level. Means with same letters are not significantly different. The data are shown as means ± SD (standard deviation). EWPE, ear weight per ear; CWPE, cob weight per ear; EL, ear length; ED, ear diameter; CD, cob diameter; RN, row number; KNPR, kernel number per ear; KWPE, kernel weight per ear; RKP, rate of kernel production. (JPG 513 kb)

Additional file 2: **Figure S2.** Phenotypic variance explained (PVE) by QTL and epistasis interaction (EPI, digenic interaction) effects for nine yield-related traits in the RILs, IF_2_ population and the MPH dataset. The total PVE for QTL and epistasis was estimated on ICIM-ADD and ICIM-EPI, respectively. ICIM, inclusive composite interval mapping. RIL, recombinant inbred line. IF_2_, immortalized F_2_. MPH, mid-parent heterosis. EWPE, ear weight per ear; CWPE, cob weight per ear; EL, ear length; ED, ear diameter; CD, cob diameter; RN, row number; KNPR, kernel number per ear; KWPE, kernel weight per ear; RKP, rate of kernel production. (PPTX 37 kb)

Additional file 3: **Table S1.** List of abbreviations and definitions for the yield-related traits recorded at harvest in a sample of ten ears per plot. (DOCX 16 kb)

Additional file 4: **Table S2.** The performance of yield-related traits in the RILs, the IF_2_ population_,_ and the MPH dataset across all environments. EWPE, ear weight per ear; CWPE, cob weight per ear; ED, ear diameter; CD, cob diameter; EL, ear length; RN, row number; KNPR, kernel number per row; KWPE, kernel weight per row; RKP, rate of kernel production. ^a^ F1, The cross of 08–641 × YE478. RIL, the recombinant inbred lines. ^b^ IF_2_ and MPH indicate the immortalized F_2_ population and mid-parent heterosis, respectively. ^c^ Mean ± SE represents mean across all environments ± standard error. ^d^ MPH (%) = (F_1_-(P_1_ + P_2_)/2)/((P_1_ + P_2_)/2)*100%. (DOCX 18 kb)

Additional file 5: **Table S3.** Pearson’s correlation coefficients among yield-related traits in the RILs^a^ across four environments, the IF_2_^c^ populations across three environments, and the mid-parent heterosis (MPH) dataset. * Significant at the 0.05 probability level. ** Significant at the 0.01 probability level. The absolute values of Pearson’s correlation coefficients (*r*^2^) with bold are equal to or larger than 0.5. EWPE, ear weight per ear; CWPE, cob weight per ear; ED, ear diameter; CD, cob diameter; EL, ear length; RN, row number; KNPR, kernel number per row; KWPE, kernel weight per row; RKP, rate of kernel production. ^a^ Pearson’s correlation coefficients (*r*^2^) in the first row for each trait are for the RILs. ^b^ Pearson’s correlation coefficients (*r*^2^) in the second row for each trait are for the IF_2_ population. ^c^ Pearson’s correlation coefficients (*r*^2^) in the third row for each trait are for mid-parent heterosis. (DOCX 1433 kb)

Additional file 6: **Table S4.** Main feature of QTLs for yield-related traits and for mid-parental heterosis for those traits from the RILs and IF_2_ population via single environment analysis. ^a^ The nomination of QTL is made as follows: a “q” standing for the abbreviation of QTL, the abbreviation of the trait, an “S” standing for single environment analyses, a number standing for chromosome, and another for physical order. EWPE, ear weight per ear; CWPE, cob weight per ear; ED, ear diameter; CD, cob diameter; EL, ear length; RN, row number; KNPR, kernel number per row; KWPE, kernel weight per row; RKP, rate of kernel production. ^b^ RIL, recombinant inbred lines; IF_2_, the immortalized F_2_; MPH, mid-parental heterosis derived from the RIL and IF_2_ populations. ^c^ Env, environment; E1, 2014Jinghong; E2, 2015Jinghong; E3, 2016Chongzhou; E4, 2016Jinghong. ^d^ Chr, chromosome. ^e^ A, additive effect; Negative additive values indicate that the allele for increasing trait value is contributed by the parent 08–641; Positive additive values indicate that the allele for increasing trait value is contributed by another parent Ye478. ^f^ D, dominance effect. ^g^ PVE, phenotypic variance explained by QTL. (DOCX 70 kb)

Additional file 7: **Table S5.** Pearson’s correlation coefficients between phenotypic performance per se in IF_2_ population and mid-parent heterosis for those traits. ^***^ Significant at *p* < 0.0001. (DOCX 15 kb)

Additional file 8: **Table S6.** Pearson’s correlation coefficients between phenotypic variation and genome marker heterozygosity. ^a^ Correlation between performance per se in IF_2_ population and percentage of heterozygous loci. ^b^ Correlation between mid-parent heterosis and the percentage of heterozygous loci. ^***^, significant at *p* < 0.0001 level; ^**^, significant at *p* < 0.01 level; ^*^, significant at *p* < 0.05 level; NS, not significant. EWPE, ear weight per ear; CWPE, cob weight per ear; ED, ear diameter; CD, cob diameter; EL, ear length; RN, row number; KNPR, kernel number per row; KWPE, kernel weight per row; RKP, rate of kernel production. (DOCX 16 kb)

Additional file 9: **Table S7.** Epistatic interactions for yield-related traits and mid-parent heterosis detected in the RILs and IF_2_ population across all environments. ^a^ EWPE, ear weight per ear; CWPE, cob weight per ear; CD, cob diameter; EL, ear length; RN, row number; KNPR, kernel number per row; KWPE, kernel weight per row; RKP, rate of kernel production. ^b^ Interval of the first QTL i. ^c^ Chromosome ID at the first scanning position. ^d^ A_i Additive effect of the QTL i. ^e^ D_i Dominance effect of the QTL i. ^f^ Interval of the second QTL j. ^g^ Chromosome ID at the second scanning position. ^h^ A_j Additive effect of the QTL j. ^i^ D_j Dominance effect of the QTL j. ^j^ AA: Estimated additive by the additive effect of QTLs at the two scanning positions. ^k^ AD: Estimated additive by dominance effect of QTLs at the two scanning positions. ^m^ DA: Estimated dominance by the additive effect of QTLs at the two scanning positions. ^n^ DD: Estimated dominance by dominance effect of QTLs at the two scanning positions. ^l^ *h*^2^(%): Heritability for epistatic QTL effects. The interval in bold was co-located in QTLs associated with the same trait via combined analysis across all environments. ^*^, ^**^, ^***^ indicate significance at *p* < 0.05, *p* < 0.01, and *p* < 0.0001, respectively. (DOCX 26 kb)

Additional file 10: **Table S8.** QTL × environment interactions for yield-related traits detected in the RILs under four environments and the IF_2_ population under three environments. ^a^ RIL, recombinant inbred lines; IF_2_, the immortalized F_2_. ^b^ AE indicates the additive by designed environment interaction effect. E1, E2, E3, and E4, represent 2014JH, 2015JH, 2016CZ, and 2016JH, respectively. ^c^ *h*^2^(AE, %) is the contribution rate of additive by environment interaction effect. ^d^ DE indicates the dominance by designed environment interaction effect. E1, E2, E3, and E4, represent 2014JH, 2015JH, 2016CZ, and 2016JH, respectively. ^e^ *h*^2^(DE, %) is the contribution rate of dominance by environment interaction effect. ^*^, ^**^, ^***^ indicate significance at *p* < 0.05, *p* < 0.01, and *p* < 0.0001, respectively. The interval in bold was co-located in QTLs associated with the same trait via combined analysis across all environments. (DOCX 21 kb)

Additional file 11: Genotypic and phenotypic data of the RIL and IF populations for the QTL analyses. (XLSX 1583 kb)

## Data Availability

Supporting data are available in Additional file 11, and materials are available from the authors upon request.

## Abbreviations

AA: Additive × additive interaction
AD: Additive × dominance interaction
BLUP: Best linear unbiased predictor
CD: Cob diameter
CWPE: Cob weight per ear
DA: Dominance × additive interaction
DD: Dominance × dominance interaction
ED: Ear diameter
EL: Ear length
EPI: Epistatic interaction
EWPE: Ear weight per ear
ICIM: Inclusive composite interval mapping
IF_2_: The immortalized F_2_
KNPR: Kernel number per row
KWPE: Kernel weight per ear
MCIM: Mixed-model-based composite interval mapping
MPH: Mid-parent heterosis
QTL: Quantitative trait loci
RIL: Recombinant inbred lines
RKP: Rate of kernel production
RN: Row number
SMA: Single marker analysis
SNP: Single nucleotide polymorphism
