# Supplementary figures and images for: Genetic dissection of yield-related traits and mid-parent heterosis for those traits in maize (Zea mays L.)
Source: BMC Plant Biol. 2019 Sep 9;19:392. doi: 10.1186/s12870-019-2009-2 (PMC6734583; doi:10.1186/s12870-019-2009-2)

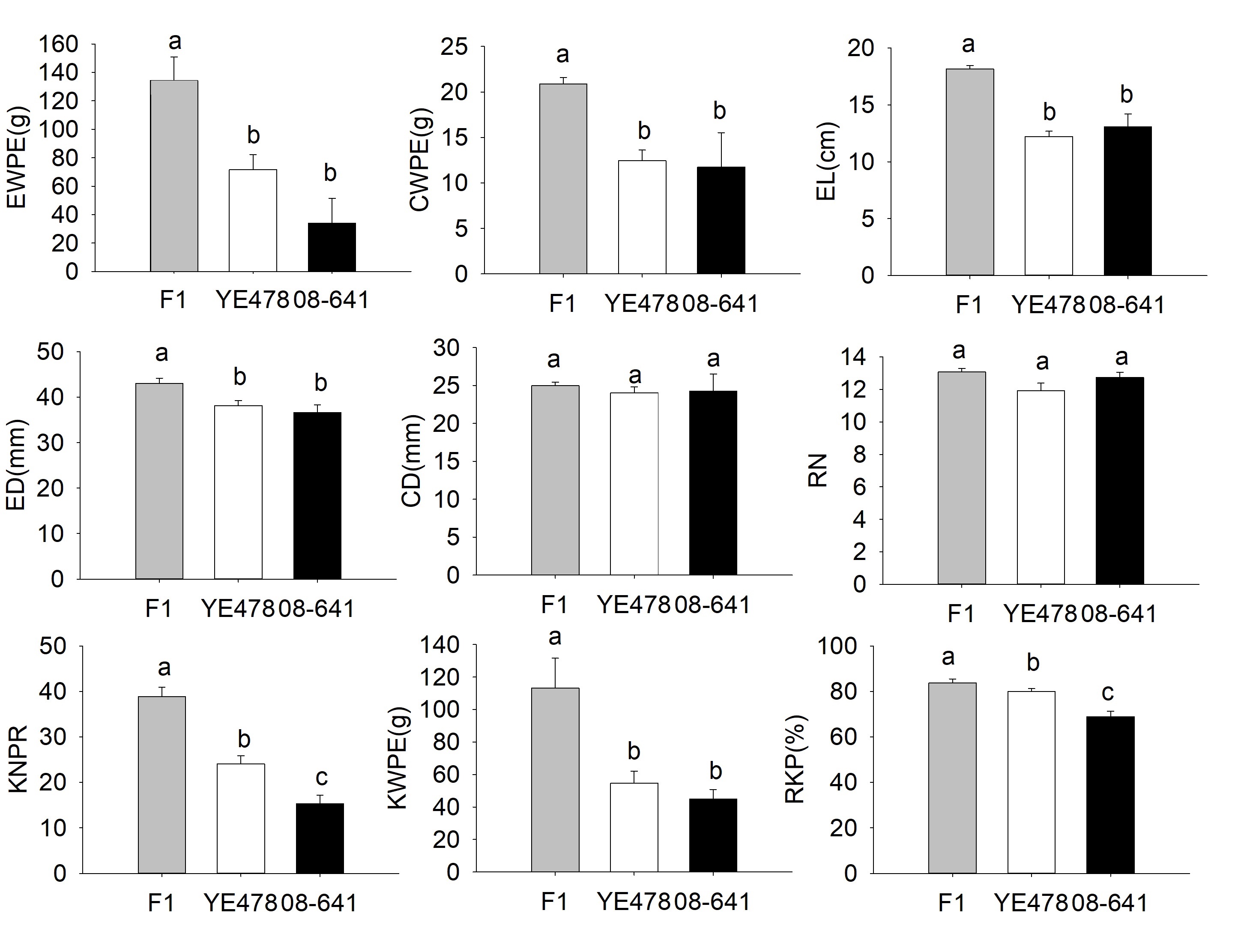

Supplement: Supplementary file 1 — Figure S1. The performance of parental inbreds of the RIL population (YE478 and 08–641) and the hybrid F1 across four environments. The Duncan multiple range test was used for the comparison of means. Genotypes with lower-case letters were significantly different at the 0.05 probability level. Means with same letters are not significantly different. The data are shown as means ± SD (standard deviation). EWPE, ear weight per ear; CWPE, cob weight per ear; EL, ear length; ED, ear diameter; CD, cob diameter; RN, row number; KNPR, kernel number per ear; KWPE, kernel weight per ear; RKP, rate of kernel production. (JPG 513 kb) [file 12870_2019_2009_MOESM1_ESM.jpg]
