# Supplementary material for: Genetic dissection of yield-related traits and mid-parent heterosis for those traits in maize (Zea mays L.)
Source: BMC Plant Biol. 2019 Sep 9;19:392. doi: 10.1186/s12870-019-2009-2 (PMC6734583; doi:10.1186/s12870-019-2009-2)
Supplement: Supplementary file 2 — Figure S2. Phenotypic variance explained (PVE) by QTL and epistasis interaction (EPI, digenic interaction) effects for nine yield-related traits in the RILs, IF2 population and the MPH dataset. The total PVE for QTL and epistasis was estimated on ICIM-ADD and ICIM-EPI, respectively. ICIM, inclusive composite interval mapping. RIL, recombinant inbred line. IF2, immortalized F2. MPH, mid-parent heterosis. EWPE, ear weight per ear; CWPE, cob weight per ear; EL, ear length; ED, ear diameter; CD, cob diameter; RN, row number; KNPR, kernel number per ear; KWPE, kernel weight per ear; RKP, rate of kernel production. (PPTX 37 kb) [file 12870_2019_2009_MOESM2_ESM.pptx]

## Slide 1
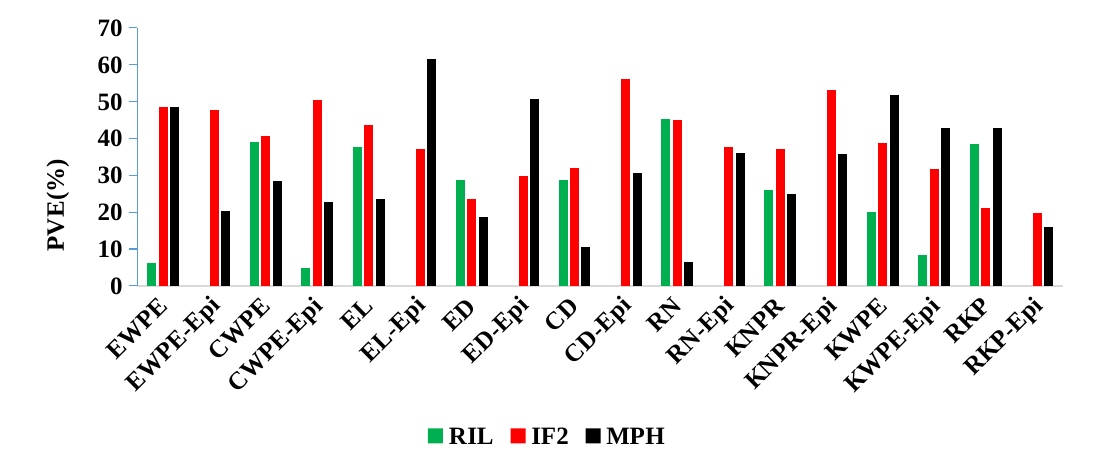

### Chart
| Category | RIL | IF2 | MPH |
|---|---|---|---|
| EWPE | 6.3 | 48.4 | 48.5 |
| EWPE-Epi | None | 47.592800000000004 | 20.2173 |
| CWPE | 38.9 | 40.5 | 28.3 |
| CWPE-Epi | 4.8573 | 50.3128 | 22.629800000000003 |
| EL | 37.6 | 43.6 | 23.6 |
| EL-Epi | None | 37.231 | 61.4032 |
| ED | 28.6 | 23.6 | 18.7 |
| ED-Epi | None | 29.679599999999994 | 50.5702 |
| CD | 28.8 | 31.9 | 10.5 |
| CD-Epi | None | 55.94649999999999 | 30.551000000000002 |
| RN | 45.2 | 44.9 | 6.6 |
| RN-Epi | None | 37.738200000000006 | 35.93 |
| KNPR | 26.0 | 37.1 | 25.0 |
| KNPR-Epi | None | 52.97650000000001 | 35.827 |
| KWPE | 20.1 | 38.8 | 51.6 |
| KWPE-Epi | 8.4825 | 31.7651 | 42.7526 |
| RKP | 38.4 | 21.2 | 42.7 |
| RKP-Epi | None | 19.8968 | 16.0272 |
