# Supplementary material for: Genetic dissection of yield-related traits and mid-parent heterosis for those traits in maize (Zea mays L.)
Source: BMC Plant Biol. 2019 Sep 9;19:392. doi: 10.1186/s12870-019-2009-2 (PMC6734583; doi:10.1186/s12870-019-2009-2)
Supplement: Supplementary file 3 — Table S1. List of abbreviations and definitions for the yield-related traits recorded at harvest in a sample of ten ears per plot. (DOCX 16 kb) [file 12870_2019_2009_MOESM3_ESM.docx]

Table S1 List of abbreviations and definitions for the yield-related traits recorded at harvest in a sample of ten ears per plot

| Trait | Name and definition |
| --- | --- |
| EWPE (g) | Ear weight per ear: average ear weight per ear |
| CWPE (g) | Cob weight per ear: average cob weight |
| EL (cm) | Ear length: average ear length measured from the bottom to the top of the ear |
| ED (mm) | Ear diameter: average diameter of the middle ear taken on the narrowest direction using a digital caliper |
| CD (mm) | Cob diameter: average diameter of the middle cob taken on the narrowest direction using a digital caliper |
| RN | Row number: average number of rows per ear |
| KNPR | Kernel number per row: average number of kernels per ear row |
| KWPE (g) | Kernel weight per ear: average kernel weight per ear at 14 % kernel humidity estimated using the formula “(EWPE-CWPE)*(1-humility%)/(1-14%)” |
| RKP (%) | Rate of kernel production: average percentage of kernel weight on ear weight calculated with the formula (EWPE-CWPE)/EWPE*100% |
|  |  |
