# Supplementary material for: Genetic dissection of yield-related traits and mid-parent heterosis for those traits in maize (Zea mays L.)
Source: BMC Plant Biol. 2019 Sep 9;19:392. doi: 10.1186/s12870-019-2009-2 (PMC6734583; doi:10.1186/s12870-019-2009-2)
Supplement: Supplementary file 4 — Table S2. The performance of yield-related traits in the RILs, the IF2 population, and the MPH dataset across all environments. EWPE, ear weight per ear; CWPE, cob weight per ear; ED, ear diameter; CD, cob diameter; EL, ear length; RN, row number; KNPR, kernel number per row; KWPE, kernel weight per row; RKP, rate of kernel production. a F1, The cross of 08–641 × YE478. RIL, the recombinant inbred lines. b IF2 and MPH indicate the immortalized F2 population and mid-parent heterosis, respectively. c Mean ± SE represents mean across all environments ± standard error. d MPH (%) = (F1-(P1 + P2)/2)/((P1 + P2)/2)*100%. (DOCX 18 kb) [file 12870_2019_2009_MOESM4_ESM.docx]

Table S2 The performance of yield-related traits in the RILs, the IF_2_ population_,_ and the MPH dataset across all environments

| Trait | F1^a^ | | RIL^a^ | | IF_2_^b^ | | MPH^b^ | |
| --- | --- | --- | --- | --- | --- | --- | --- | --- |
|  | Mean±SE^c^ | MPH±SE^c^ /MPH(%)^d^ | Mean±SE^c^ | Range | Mean±SE^c^ | Range | Mean±SE^c^/MPH(%) | Range |
| EWPE (g) | 134.18±33.42 | 88.07±29.02/153.7% | 59.40±0.83 | 25.85-115.34 | 118.56±1.21 | 48.13-220.51 | 57.93±1.23/102.9% | 2.83-146.18 |
| CWPE (g) | 20.87±1.49 | 9.33±0.78/73.4% | 13.16±0.20 | 3.01-27.95 | 19.89±0.21 | 8.50-32.50 | 6.48±0.19/51.7% | -1.51-17.16 |
| ED (mm) | 43.01±2.33 | 7.65±2.08/15.1% | 35.85±0.14 | 27.49-43.43 | 41.93±0.12 | 34.95-49.42 | 5.57±0.13/15.8% | 0.00-13.94 |
| CD (mm) | 24.96±0.94 | 2.94±1.36/3.5% | 22.14±0.11 | 17.42-27.54 | 24.50±0.10 | 20.69-30.12 | 3.76±0.10/17.2% | -1.98-11.49 |
| EL (cm) | 18.15±0.61 | 6.15±0.07/43.5% | 13.65±0.09 | 9.09-20.61 | 16.68±0.079 | 12.12-21.24 | 2.98±0.07/22.2% | -1.31-7.00 |
| RN | 13.07±0.42 | 1.29±1.14/6% | 12.28±0.07 | 9.16-16.13 | 13.20±0.06 | 10.72-16.21 | 0.85±0.04/7% | -1.32-2.79 |
| KNPR | 38.87±4.12 | 19.72±2.72/97.5% | 20.66±0.26 | 9.43-32.43 | 35.31±0.22 | 21.92-46.13 | 14.43±0.23/73.1% | 0.55-27.57 |
| KWPE (g) | 113.25±36.63 | 102.77/127.4% | 47.43±0.67 | 12.16-94.14 | 95.24±1.01 | 58.05-188.73 | 47.36±1.10/105.7% | -16.63-126.27 |
| RKP | 0.84±0.03 | 0.15/12.6% | 0.70±0.01 | 0.39-0.86 | 0.82±0.00 | 0.72-0.88 | 0.11±0.00/16.4% | 0.02-0.33 |

EWPE, ear weight per ear; CWPE, cob weight per ear; ED, ear diameter; CD, cob diameter; EL, ear length; RN, row number; KNPR, kernel number per row; KWPE, kernel weight per row; RKP, rate of kernel production.

^a^ F1, The cross of 08-641 × YE478. RIL, recombinant inbred lines.

^b^ IF_2_ and MPH indicate the immortalized F_2_ population and mid-parent heterosis, respectively.

^c^ Mean±SE represents mean across all environments ± standard error.

^d^ MPH(%)=(F_1_-(P_1_+P_2_)/2)/((P_1_+P_2_)/2)*100%.
