# Supplementary material for: Genetic dissection of yield-related traits and mid-parent heterosis for those traits in maize (Zea mays L.)
Source: BMC Plant Biol. 2019 Sep 9;19:392. doi: 10.1186/s12870-019-2009-2 (PMC6734583; doi:10.1186/s12870-019-2009-2)
Supplement: Supplementary file 5 — Table S3. Pearson’s correlation coefficients among yield-related traits in the RILsa across four environments, the IF2c populations across three environments, and the mid-parent heterosis (MPH) dataset. * Significant at the 0.05 probability level. ** Significant at the 0.01 probability level. The absolute values of Pearson’s correlation coefficients (r2) with bold are equal to or larger than 0.5. EWPE, ear weight per ear; CWPE, cob weight per ear; ED, ear diameter; CD, cob diameter; EL, ear length; RN, row number; KNPR, kernel number per row; KWPE, kernel weight per row; RKP, rate of kernel production. a Pearson’s correlation coefficients (r2) in the first row for each trait are for the RILs. b Pearson’s correlation coefficients (r2) in the second row for each trait are for the IF2 population. c Pearson’s correlation coefficients (r2) in the third row for each trait are for mid-parent heterosis. (DOCX 1433 kb) [file 12870_2019_2009_MOESM5_ESM.docx]

Table S3 Pearson’s correlation coefficients among yield-related traits in the RILs^a^ across four environments, the IF_2_^c^ populations across three environments, and mid-parent heterosis (MPH).

| Trait | Data | EWPE | CWPE | EL | ED | CD | RN | KNPR | KWPE | RKP |
| --- | --- | --- | --- | --- | --- | --- | --- | --- | --- | --- |
| EWPE | RIL^a^ | 1 |  |  |  |  |  |  |  |  |
|  | IF_2_^b^ | 1 |  |  |  |  |  |  |  |  |
|  | MPH^c^ | 1 |  |  |  |  |  |  |  |  |
| CWPE | RIL^a^ | **0.58^**^** | 1 |  |  |  |  |  |  |  |
|  | IF_2_^b^ | **0.70^**^** | 1 |  |  |  |  |  |  |  |
|  | MPH^c^ | **0.75^**^** | 1 |  |  |  |  |  |  |  |
| EL | RIL^a^ | **0.50^**^** | **0.60^**^** | 1 |  |  |  |  |  |  |
|  | IF_2_^b^ | **0.68^**^** | **0.50^**^** | 1 |  |  |  |  |  |  |
|  | MPH^c^ | **0.76^**^** | **0.67^**^** | 1 |  |  |  |  |  |  |
| ED | RIL^a^ | **0.60^**^** | **0.55^**^** | 0.22^**^ | 1 |  |  |  |  |  |
|  | IF_2_^b^ | **0.72^**^** | **0.59^**^** | 0.29^**^ | 1 |  |  |  |  |  |
|  | MPH^c^ | **0.73^**^** | **0.62^**^** | **0.53^**^** | 1 |  |  |  |  |  |
| CD | RIL^a^ | 0.39^**^ | **0.68^**^** | 0.23^**^ | **0.76^**^** | 1 |  |  |  |  |
|  | IF_2_^b^ | 0.44^**^ | **0.59^**^** | 0.19^**^ | **0.78^**^** | 1 |  |  |  |  |
|  | MPH^c^ | 0.39^**^ | **0.54^**^** | 0.31^**^ | **0.73^**^** | 1 |  |  |  |  |
| RN | RIL^a^ | 0.29^**^ | 0.17^**^ | -0.04 | **0.53^**^** | 0.46^**^ | 1 |  |  |  |
|  | IF_2_^b^ | 0.40^**^ | 0.18^**^ | 0.06 | **0.50^**^** | 0.36^**^ | 1 |  |  |  |
|  | MPH^c^ | 0.46^**^ | 0.26^**^ | 0.26^**^ | **0.49^**^** | 0.27^**^ | 1 |  |  |  |
| KNPR | RIL^a^ | **0.77^**^** | 0.21^**^ | 0.41^**^ | 0.34^**^ | 0.12^*^ | 0.19^**^ | 1 |  |  |
|  | IF_2_^b^ | **0.62^**^** | 0.19^**^ | **0.63^**^** | 0.32^**^ | 0.1 | 0.24^**^ | 1 |  |  |
|  | MPH^c^ | **0.69^**^** | 0.37^**^ | **0.64^**^** | 0.48^**^ | 0.15^**^ | 0.29^**^ | 1 |  |  |
| KWPE | RIL^a^ | **0.82^**^** | 0.31^**^ | 0.38^**^ | **0.54^**^** | 0.24^**^ | 0.28^**^ | **0.70^**^** | 1 |  |
|  | IF_2_^b^ | **0.97^**^** | **0.57^**^** | **0.64^**^** | **0.67^**^** | 0.38^**^ | 0.43^**^ | **0.60^**^** | 1 |  |
|  | MPH^c^ | **0.94^**^** | **0.66^**^** | **0.71^**^** | **0.67^**^** | 0.35^**^ | 0.43^**^ | **0.63^**^** | 1 |  |
| RKP | RIL^a^ | 0.39^**^ | -0.26^**^ | -0.01 | 0.22^**^ | -0.11 | 0.18^**^ | **0.66^**^** | **0.50^**^** | 1 |
|  | IF_2_^b^ | 0.39^**^ | -0.34^**^ | 0.22^**^ | 0.18^**^ | -0.18^**^ | 0.27^**^ | **0.61^**^** | 0.47^**^ | 1 |
|  | MPH^c^ | 0.35^**^ | 0.03 | 0.12^*^ | 0.22^**^ | 0.06 | 0.17^**^ | **0.50^**^** | 0.35^**^ | 1 |

* Significant at the 0.05 probability level. ** Significant at the 0.01 probability level. The absolute values of Pearson’s correlation coefficients (r2) with bold are equal to or larger than 0.5. EWPE, ear weight per ear; CWPE, cob weight per ear; ED, ear diameter; CD, cob diameter; EL, ear length; RN, row number; KNPR, kernel number per row; KWPE, kernel weight per row; RKP, rate of kernel production.

^a^ Pearson’s correlation coefficients (r2) in the first row for each trait are for the RILs.

^b^ Pearson’s correlation coefficients (r2) in the second row for each trait are for the IF_2_ population.

^c^ Pearson’s correlation coefficients (r2) in the third row for each trait are for mid-parent heterosis.


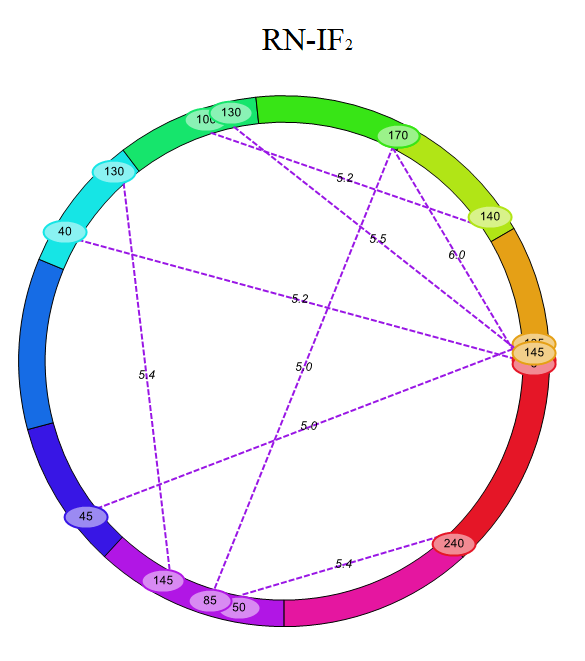

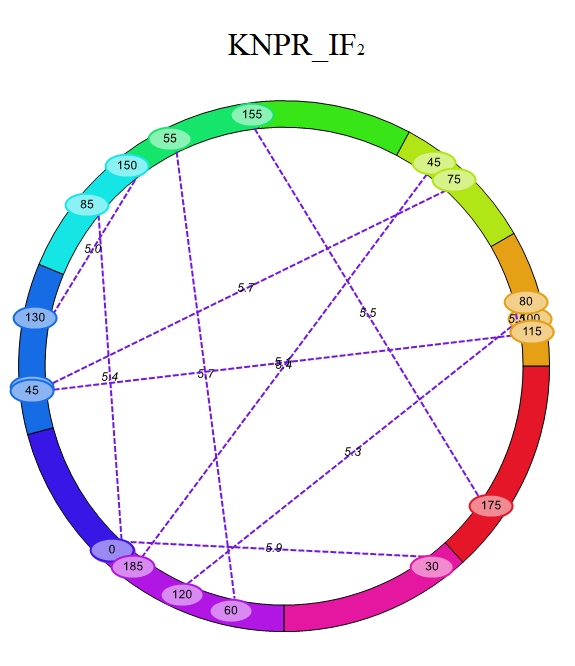

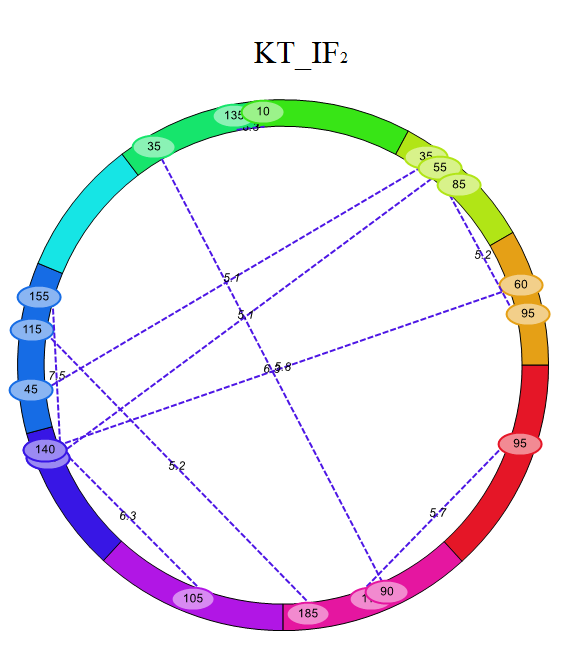

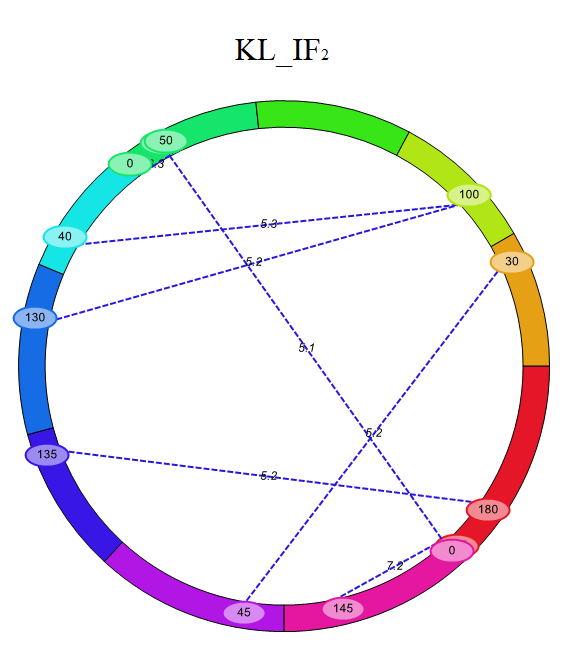

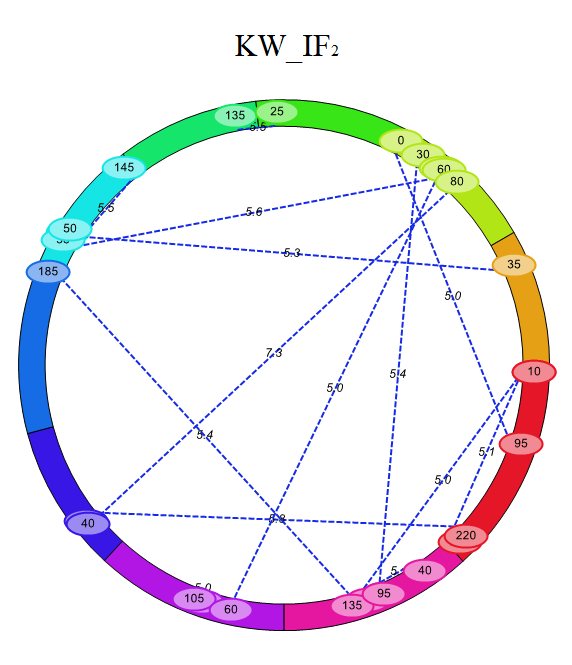

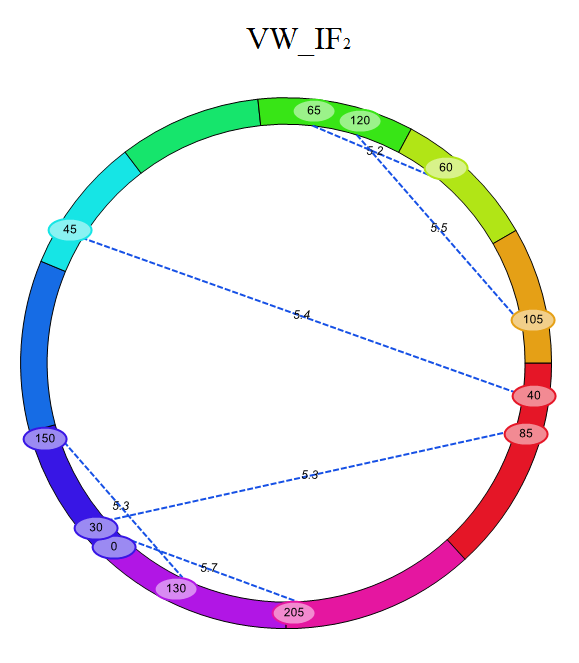

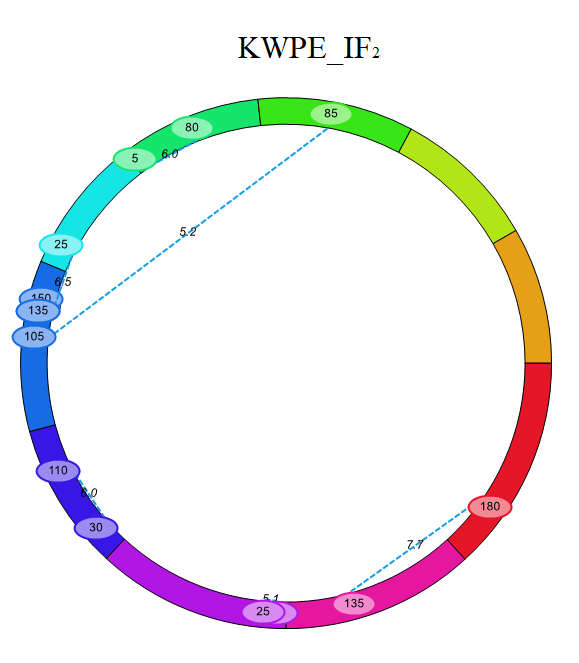

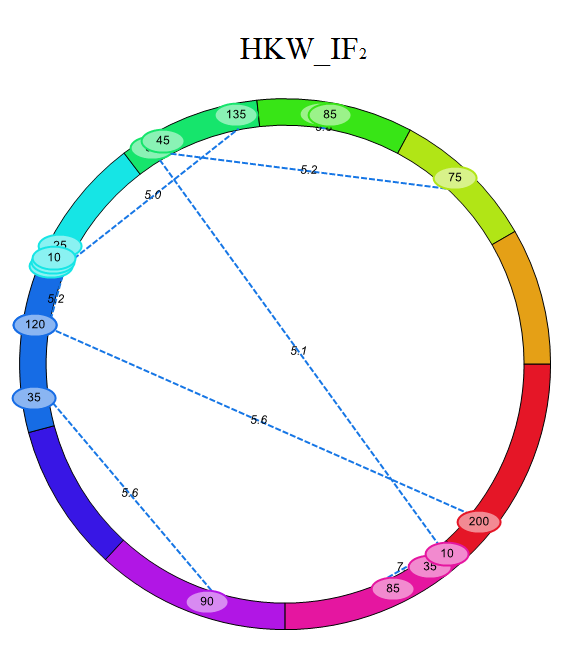

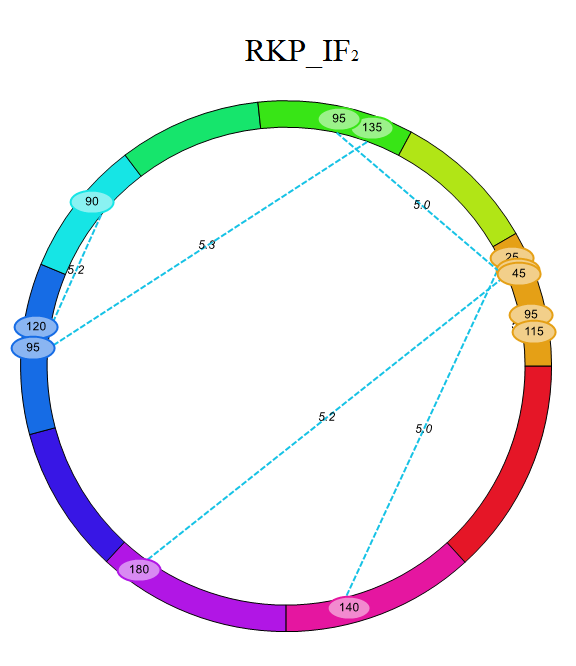

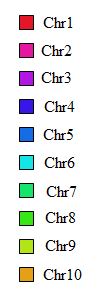


Fig. S5 Epistasis interaction of yield related traits in the IF_2_ population (LOD>=5.0). The dotted line connected to two chromosomes represents a pair of significant epistasis QTLs. The LOD scores of QTLs are shown in the dotted lines. The genetic positions of significant epistasis QTLs are shown on the chromosomes.
