# Supplementary material for: Genetic dissection of yield-related traits and mid-parent heterosis for those traits in maize (Zea mays L.)
Source: BMC Plant Biol. 2019 Sep 9;19:392. doi: 10.1186/s12870-019-2009-2 (PMC6734583; doi:10.1186/s12870-019-2009-2)
Supplement: Supplementary file 6 — Table S4. Main feature of QTLs for yield-related traits and for mid-parental heterosis for those traits from the RILs and IF2 population via single environment analysis. a The nomination of QTL is made as follows: a “q” standing for the abbreviation of QTL, the abbreviation of the trait, an “S” standing for single environment analyses, a number standing for chromosome, and another for physical order. EWPE, ear weight per ear; CWPE, cob weight per ear; ED, ear diameter; CD, cob diameter; EL, ear length; RN, row number; KNPR, kernel number per row; KWPE, kernel weight per row; RKP, rate of kernel production. b RIL, recombinant inbred lines; IF2, the immortalized F2; MPH, mid-parental heterosis derived from the RIL and IF2 populations. c Env, environment; E1, 2014Jinghong; E2, 2015Jinghong; E3, 2016Chongzhou; E4, 2016Jinghong. d Chr, chromosome. e A, additive effect; Negative additive values indicate that the allele for increasing trait value is contributed by the parent 08–641; Positive additive values indicate that the allele for increasing trait value is contributed by another parent Ye478. f D, dominance effect. g PVE, phenotypic variance explained by QTL. (DOCX 70 kb) [file 12870_2019_2009_MOESM6_ESM.docx]

Table S4 Main feature of QTLs for yield-related traits and for mid-parental heterosis for those traits from the RILs and IF_2_ population via single environment analysis

| QTL^a^ | Data^b^ | Env^c^ | Chr^d^ | Position (cM) | Range(cM) | Left Marker | Right Marker | LOD | A^e^ | D^f^ |  | PVE(%)^g^ |
| --- | --- | --- | --- | --- | --- | --- | --- | --- | --- | --- | --- | --- |
| *qEWPES1-1* | IF_2_ | E2 | 1 | 47 | 45.5-48.5 | *PZE-101043670* | *PZE-101043682* | 2.59 | -4.1 | 1.39 |  | 12.59 |
| *qEWPES1-2* | IF_2_ | E3 | 1 | 63 | 61.5-64.5 | *SYN13385* | *SYN37775* | 20.14 | -14.41 | -0.23 |  | 15.16 |
| *qEWPES1-3* | IF_2_ | E3 | 1 | 70 | 68.5-71.5 | *PZE-101065758* | *PZE-101071898* | 10.4 | 10.2 | -1.31 |  | 7.06 |
| *qEWPES1-4* | IF_2_ | E4 | 1 | 97 | 94.5-98.5 | *PZE-101084850* | *PZE-101087156* | 2.97 | -0.09 | 4.9 |  | 0.49 |
| *qEWPES2-1* | IF_2_ | E3 | 2 | 50 | 48.5-51.5 | *PZE-102056669* | *SYN28948* | 4.47 | -2.35 | 8.8 |  | 2.89 |
| *qEWPES2-2* | MPH | E3 | 2 | 62 | 61.5-62.5 | *PZA02450.1* | *SYN21924* | 4.68 |  | 11.11 |  | 1.84 |
| *qEWPES2-3* | RIL | E3 | 2 | 95 | 93.5-95.5 | *PZE-102111018* | *PZE-102110333* | 3.73 | -4.77 |  |  | 6.31 |
| *qEWPES2-4* | MPH | E3 | 2 | 112 | 109.5-114.5 | *PZE-102125779* | *PZA03661.3* | 2.9 |  | 6.05 |  | 1 |
|  | MPH | E4 | 2 | 112 | 107.5-112.5 | *PZE-102125779* | *PZA03661.3* | 3.89 |  | 6.34 |  | 1.53 |
| *qEWPES2-5* | IF_2_ | E4 | 2 | 142 | 140.5-143.5 | *PZE-102149656* | *PZE-102153048* | 3.89 | -3.76 | -1.43 |  | 0.54 |
| *qEWPES3-1* | IF_2_ | E3 | 3 | 72 | 71.5-72.5 | *PZE-103041998* | *SYN18260* | 2.77 | 0.2 | 7.04 |  | 1.79 |
|  | MPH | E2 | 3 | 72 | 70.5-74.5 | *PZE-103041998* | *SYN18260* | 2.56 |  | 2.95 |  | 5.03 |
| *qEWPES3-2* | MPH | E2 | 3 | 166 | 161.5-168.5 | *SYN28063* | *PZE-103157755* | 4.75 |  | 0.92 |  | 10.39 |
| *qEWPES3-3* | RIL | E2 | 3 | 177 | 175.5-181.5 | *PZE-103161091* | *PZE-103163529* | 3.88 | 2.4 |  |  | 5.57 |
| *qEWPES3-4* | MPH | E2 | 3 | 196 | 189.5-199.5 | *PZE-103171163* | *SYN6986* | 2.51 |  | 7.74 |  | 6.95 |
| *qEWPES4-1* | IF_2_ | E4 | 4 | 2 | 0-4.5 | *PZE-104010113* | *PZE-104012412* | 2.68 | -2.39 | 2.97 |  | 0.39 |
| *qEWPES4-2* | IF_2_ | E4 | 4 | 76 | 75.5-76.5 | *PZE-104070126* | *PZE-104071269* | 20.05 | -1.78 | -12.98 |  | 3.43 |
|  | IF_2_ | E4 | 4 | 78 | 77.5-78.5 | *PZE-104071269* | *PZE-104072142* | 26.54 | -1.13 | 14.9 |  | 4.59 |
| *qEWPES4-3* | MPH | E3 | 4 | 106 | 104.5-106.5 | *PZE-104090796* | *PZE-104093153* | 4.52 |  | 1.58 |  | 1.61 |
| *qEWPES5-1* | MPH | E3 | 5 | 71 | 70.5-72.5 | *PZE-105045328* | *PZE-105045828* | 4.92 |  | 0.94 |  | 1.73 |
| *qEWPES5-2* | IF_2_ | E3 | 5 | 101 | 100.5-101.5 | *PZE-105102176* | *PZE-105101867* | 3.25 | 0.67 | 7.56 |  | 2.1 |
| *qEWPES6-1* | MPH | E3 | 6 | 104 | 103.5-104.5 | *PZE-106083588* | *PZE-106080884* | 4.04 |  | 10.29 |  | 1.49 |
| *qEWPES6-2* | IF_2_ | E3 | 6 | 110 | 104.5-115.5 | *PZE-106083873* | *PZE-106115356* | 4.79 | -0.29 | 12.02 |  | 5.25 |
| *qEWPES7-1* | RIL | E4 | 7 | 46 | 39.5-51.5 | *PZE-107012245* | *SYN24186* | 3.31 | -3.04 |  |  | 5.77 |
|  | IF_2_ | E3 | 7 | 48 | 43.5-51.5 | *PZE-107012245* | *SYN24186* | 2.76 | -1.27 | 8.7 |  | 2.64 |
| *qEWPES7-2* | IF_2_ | E4 | 7 | 56 | 52.5-58.5 | *PZE-107019133* | *PZE-107033682* | 4.58 | -4.26 | 3 |  | 0.72 |
| *qEWPES7-3* | MPH | E3 | 7 | 70 | 66.5-76.5 | *PZE-107057229* | *PZE-107081317* | 4.15 |  | 12.04 |  | 2.06 |
| *qEWPES7-4* | IF_2_ | E3 | 7 | 94 | 92.5-94.5 | *PZE-107088270* | *PZE-107088998* | 2.7 | -1.81 | 7.03 |  | 1.77 |
| *qEWPES8* | MPH | E3 | 8 | 133 | 130.5-138.5 | *SYN13209* | *SYN30185* | 2.74 |  | -5.88 |  | 0.96 |
| *qEWPES9-1* | IF_2_ | E3 | 9 | 53 | 52.5-53.5 | *PZE-109023988* | *PZE-109026030* | 4.99 | -3.78 | 7.29 |  | 3.1 |
|  | MPH | E4 | 9 | 55 | 53.5-58.5 | *PZE-109026030* | *PZE-109027216* | 3.64 |  | 1.52 |  | 1.43 |
| *qEWPES9-2* | MPH | E3 | 9 | 84 | 83.5-85.5 | *PZE-109037929* | *PZA03595.2* | 53.23 |  | 4.21 |  | 29.84 |
| *qEWPES9-3* | MPH | E2 | 9 | 101 | 97.5-102.5 | *PZE-109061922* | *PZE-109062229* | 2.6 |  | 4.65 |  | 5.06 |
| *qEWPES10-1* | MPH | E4 | 10 | 50 | 49.5-50.5 | *PZE-110015504* | *PZE-110018194* | 29.73 |  | -20.15 |  | 15.04 |
|  | MPH | E4 | 10 | 52 | 51.5-52.5 | *PZE-110018194* | *SYN17647* | 2.97 |  | 6.01 |  | 1.35 |
| *qEWPES10-2* | IF_2_ | E4 | 10 | 60 | 59.5-60.5 | *PZE-110038658* | *SYN18227* | 92.5 | 26.35 | 1.43 |  | 27.59 |
| *qEWPES10-3* | RIL | E2 | 10 | 72 | 70.5-72.5 | *PZE-110049371* | *PZE-110051403* | 3.32 | 2.28 |  |  | 4.99 |
|  | RIL | E4 | 10 | 72 | 71.5-74.5 | *PZE-110049371* | *PZE-110051403* | 4.56 | 3.22 |  |  | 6.6 |
| *qEWPES10-4* | IF_2_ | E4 | 10 | 83 | 82.5-83.5 | *SYN17753* | *PZE-110074914* | 3.41 | 0.15 | 4.87 |  | 0.48 |
| *qEWPES10-5* | MPH | E3 | 10 | 125 | 120.5-127.5 | *PZE-110095199* | *PZE-110103156* | 4.37 |  | -1.15 |  | 1.59 |
| *qCWPES1-1* | MPH | E3 | 1 | 52 | 50.5-52.5 | *PZE-101049608* | *SYN450* | 5.39 |  | -0.39 |  | 4.4 |
| *qCWPES1-2* | IF_2_ | E2 | 1 | 56 | 55.5-56.5 | *SYN25114* | *PZE-101055771* | 27.44 | -2.49 | -0.15 |  | 17.15 |
|  | IF_2_ | E3 | 1 | 56 | 54.5-58.5 | *SYN25114* | *PZE-101055771* | 7.88 | -1.55 | -0.9 |  | 0.91 |
| *qCWPES1-3* | IF_2_ | E2 | 1 | 59 | 58.5-60.5 | *PZE-101058322* | *SYN36080* | 14.25 | 1.65 | -0.19 |  | 7.78 |
|  | RIL | E3 | 1 | 61 | 60.5-64.5 | *SYN36080* | *SYN13385* | 3.62 | -0.79 |  |  | 2.35 |
| *qCWPES1-4* | IF_2_ | E4 | 1 | 208 | 201.5-215.5 | *PZE-101213558* | *SYN22772* | 3.11 | -0.31 | 0.99 |  | 2.86 |
| *qCWPES2-1* | MPH | E2 | 2 | 11 | 0-20.5 | *PZE-102017443* | *SYN7604* | 3.07 |  | 0.68 |  | 5.72 |
| *qCWPES2-2* | RIL | E3 | 2 | 82 | 80.5-82.5 | *PZE-102086305* | *SYN36229* | 11.2 | -1.42 |  |  | 7.5 |
| *qCWPES2-3* | IF_2_ | E4 | 2 | 94 | 93.5-95.5 | *SYN35922* | *PZE-102111018* | 3.87 | -0.72 | -0.29 |  | 2.2 |
| *qCWPES2-4* | IF_2_ | E3 | 2 | 135 | 133.5-135.5 | *PZE-102146058* | *PZE-102147840* | 3.25 | -1.14 | 0.28 |  | 0.37 |
| *qCWPES2-5* | MPH | E4 | 2 | 142 | 140.5-143.5 | *PZE-102149656* | *PZE-102153048* | 2.66 |  | -0.7 |  | 2.99 |
|  | RIL | E3 | 2 | 145 | 143.5-146.5 | *PZE-102153564* | *PZE-102155296* | 7.49 | -1.15 |  |  | 4.92 |
| *qCWPES2-6* | RIL | E3 | 2 | 170 | 168.5-171.5 | *PZE-102173306* | *PZE-102175026* | 10.93 | 1.42 |  |  | 7.49 |
| *qCWPES2-7* | RIL | E3 | 2 | 205 | 204.5-205.5 | *SYN14631* | *PZE-102189664* | 9.5 | -1.33 |  |  | 6.55 |
|  | RIL | E4 | 2 | 206 | 203.5-209.5 | *PZE-102189664* | *PZE-102193611* | 2.97 | -0.53 |  |  | 4.67 |
| *qCWPES3-1* | MPH | E4 | 3 | 33 | 29.5-40.5 | *SYN7905* | *PZE-103018221* | 3.62 |  | 0.29 |  | 5.58 |
| *qCWPES3-2* | MPH | E3 | 3 | 217 | 216.5-217 | *PZE-103183391* | *PZE-103183701* | 5.57 |  | -0.43 |  | 4.47 |
| *qCWPES4-1* | IF_2_ | E3 | 4 | 49 | 48.5-49.5 | *PZE-104033489* | *PZE-104041535* | 4.33 | 1.24 | 0.29 |  | 0.5 |
| *qCWPES4-2* | RIL | E2 | 4 | 53 | 51.5-53.5 | *SYN25530* | *PZE-104050148* | 3.22 | 0.64 |  |  | 4.35 |
| *qCWPES4-3* | IF_2_ | E2 | 4 | 69 | 68.5-69.5 | *PZE-104053258* | *PZE-104056726* | 3.21 | -0.01 | 1.08 |  | 1.64 |
| *qCWPES4-4* | IF_2_ | E2 | 4 | 78 | 76.5-78.5 | *PZE-104071269* | *PZE-104072142* | 4.42 | 0.86 | 0.23 |  | 2.23 |
| *qCWPES5-1* | MPH | E4 | 5 | 69 | 68.5-70.5 | *PZE-105044182* | *SYN11795* | 4.64 |  | 0.26 |  | 5.57 |
| *qCWPES5-2* | MPH | E3 | 5 | 91 | 90.5-91.5 | *PZE-105093615* | *SYN32229* | 2.76 |  | -0.36 |  | 2.21 |
| *qCWPES5-3* | IF_2_ | E4 | 5 | 100 | 97.5-100.5 | *PZE-105096701* | *PZE-105101729* | 11.6 | 1.16 | 0.6 |  | 6.89 |
| *qCWPES5-4* | IF_2_ | E4 | 5 | 111 | 110.5-111.5 | *PZE-105110168* | *PZE-105111323* | 19.6 | -1.73 | 0.38 |  | 12.68 |
| *qCWPES5-5* | IF_2_ | E3 | 5 | 117 | 115.5-119.5 | *PZE-105113962* | *PZE-105115759* | 4.08 | -1.2 | -0.3 |  | 0.49 |
| *qCWPES5-6* | RIL | E3 | 5 | 121 | 120.5-122.5 | *PZE-105116229* | *SYN11021* | 6.84 | -1.11 |  |  | 4.55 |
| *qCWPES6-1* | RIL | E3 | 6 | 9 | 8.5-9.5 | *PZE-106008406* | *PZE-106020499* | 4.72 | -0.9 |  |  | 3.01 |
| *qCWPES6-2* | IF_2_ | E3 | 6 | 18 | 17.5-18.5 | *PUT-163a-18163247-1246* | *SYN30854* | 113 | -11.14 | 0.69 |  | 35.02 |
| *qCWPES6-3* | MPH | E3 | 6 | 76 | 75.5-76.5 | *PZE-106062631* | *SYN29805* | 6.03 |  | -1.9 |  | 4.9 |
| *qCWPES6-4* | MPH | E3 | 6 | 82 | 80.5-83.5 | *SYN7240* | *PZE-106067678* | 7.66 |  | 2.6 |  | 8.95 |
| *qCWPES6-5* | IF_2_ | E3 | 6 | 98 | 89.5-100.5 | *PZE-106076357* | *PZE-106083557* | 2.87 | 1.03 | 0.32 |  | 0.36 |
|  | MPH | E2 | 6 | 101 | 96.5-103.5 | *PZE-106083557* | *PZE-106083588* | 4.09 |  | 0.26 |  | 6.01 |
| *qCWPES6-6* | IF_2_ | E4 | 6 | 121 | 112.5-129.5 | *PZE-106083873* | *PZE-106115356* | 4.75 | 0.94 | 0.14 |  | 3.95 |
|  | RIL | E3 | 6 | 123 | 111.5-133.5 | *PZE-106083873* | *PZE-106115356* | 3.17 | 0.84 |  |  | 2.62 |
| *qCWPES7-1* | IF_2_ | E4 | 7 | 0 | 0-1.5 | *PZA01426.1* | *SYN10723* | 3.67 | -0.66 | -0.23 |  | 2.06 |
| *qCWPES7-2* | RIL | E2 | 7 | 84 | 83.5-84.5 | *PZE-107081442* | *PZE-107081254* | 5.29 | 0.84 |  |  | 7.27 |
| *qCWPES8* | RIL | E3 | 8 | 68 | 67.5-69.5 | *PZE-108020972* | *PZE-108021854* | 8.02 | 1.19 |  |  | 5.27 |
| *qCWPES9* | MPH | E4 | 9 | 56 | 53.5-58.5 | *PZE-109027216* | *PZE-109026940* | 4.76 |  | 0.25 |  | 5.68 |
| *qCWPES10-1* | RIL | E4 | 10 | 72 | 71.5-74.5 | *PZE-110049371* | *PZE-110051403* | 5.8 | 0.76 |  |  | 9.53 |
|  | MPH | E3 | 10 | 73 | 71.5-74.5 | *PZE-110051403* | *PZE-110054264* | 3.09 |  | 1.34 |  | 2.53 |
| *qCWPES10-2* | IF_2_ | E2 | 10 | 82 | 78.5-83.5 | *PZE-110068110* | *SYN17753* | 3.3 | 0.36 | 0.99 |  | 1.67 |
|  | MPH | E4 | 10 | 82 | 78.5-82.5 | *PZE-110068110* | *SYN17753* | 3.09 |  | 1.07 |  | 3.88 |
| *qCWPES10-3* | IF_2_ | E2 | 10 | 126 | 119.5-128.5 | *PZE-110103156* | *PZE-110104601* | 4.34 | -0.92 | 0.03 |  | 2.18 |
| *qELS1-1* | RIL | E3 | 1 | 31 | 29.5-33.5 | *PZE-101032249* | *PZE-101033801* | 3.08 | -0.35 |  |  | 3.63 |
| *qELS1-2* | IF_2_ | E3 | 1 | 50 | 48.5-52.5 | *PZE-101049608* | *SYN450* | 5.14 | -0.44 | -0.08 |  | 3.35 |
| *qELS1-3* | IF_2_ | E2 | 1 | 56 | 54.5-58.5 | *SYN25114* | *PZE-101055771* | 4.03 | -0.37 | -0.03 |  | 5.88 |
|  | RIL | E1 | 1 | 57 | 54.5-58.5 | *PZE-101055771* | *PZE-101058322* | 5.02 | -0.32 |  |  | 5.98 |
| *qELS1-4* | MPH | E2 | 1 | 115 | 110.5-137.5 | *PZE-101106605* | *SYN376* | 2.59 |  | 0.39 |  | 4.94 |
| *qELS1-5* | IF_2_ | E2 | 1 | 153 | 152.5-156.5 | *PZE-101173330* | *SYN2411* | 3.21 | 0.01 | 0.47 |  | 4.72 |
| *qELS1-6* | IF_2_ | E3 | 1 | 181 | 177.5-181.5 | *PZE-101196838* | *PZE-101194927* | 5.86 | -0.43 | 0.22 |  | 3.72 |
| *qELS1-7* | IF_2_ | E2 | 1 | 196 | 185.5-198.5 | *SYN275* | *PZE-101213558* | 2.95 | -0.27 | 0.17 |  | 4.18 |
| *qELS2-1* | IF_2_ | E3 | 2 | 0 | 0-0.5 | *PZE-102017304* | *PZE-102017443* | 5.98 | -0.47 | 0.24 |  | 3.8 |
|  | RIL | E1 | 2 | 1 | 0-6.5 | *PZE-102017443* | *SYN7604* | 3.59 | -0.27 |  |  | 4.19 |
|  | RIL | E2 | 2 | 1 | 0-8.5 | *PZE-102017443* | *SYN7604* | 3.26 | -0.3 |  |  | 4.02 |
| *qELS2-2* | RIL | E4 | 2 | 34 | 28.5-38.5 | *PZE-102037260* | *SYN35504* | 4.32 | -0.34 |  |  | 4.84 |
|  | IF_2_ | E4 | 2 | 34 | 28.5-38.5 | *PZE-102037260* | *SYN35504* | 3.05 | -0.24 | 0.01 |  | 2.84 |
| *qELS2-3* | RIL | E1 | 2 | 42 | 39.5-46.5 | *PZE-102049280* | *SYN314* | 2.84 | -0.23 |  |  | 3.19 |
|  | RIL | E3 | 2 | 44 | 41.5-46.5 | *PZE-102049280* | *SYN314* | 8.94 | -0.63 |  |  | 11.9 |
| *qELS2-4* | IF_2_ | E4 | 2 | 52 | 51.5-52.5 | *SYN28948* | *PZE-102059924* | 5.3 | 0.02 | 0.46 |  | 5.15 |
| *qELS2-5* | IF_2_ | E3 | 2 | 61 | 59.5-61.5 | *PZE-102065424* | *PZA02450.1* | 4.41 | -0.21 | 0.54 |  | 2.84 |
| *qELS2-6* | MPH | E4 | 2 | 83 | 82.5-83.5 | *PZE-102101817* | *PZE-102094429* | 14.86 |  | -0.97 |  | 9.87 |
| *qELS2-7* | IF_2_ | E2 | 2 | 94 | 92.5-94.5 | *SYN35922* | *PZE-102111018* | 2.67 | -0.31 | 0 |  | 3.87 |
| *qELS2-8* | RIL | E2 | 2 | 102 | 100.5-102.5 | *PZE-102113766* | *SYN27293* | 3.9 | -0.32 |  |  | 4.68 |
| *qELS2-9* | MPH | E3 | 2 | 106 | 105.5-106.5 | *SYN6639* | *PZE-102117120* | 4.35 |  | 0.61 |  | 2.73 |
| *qELS2-10* | MPH | E2 | 2 | 148 | 145.5-154.5 | *PZE-102154251* | *SYN7501* | 2.76 |  | 0.42 |  | 4.53 |
| *qELS3-1* | IF_2_ | E4 | 3 | 57 | 55.5-59.5 | *PZE-103029035* | *PZE-103032109* | 4.21 | 0.27 | 0.13 |  | 4 |
| *qELS3-2* | IF_2_ | E3 | 3 | 64 | 60.5-64.5 | *PZE-103033919* | *PZE-103036266* | 4.15 | 0.39 | 0.16 |  | 2.67 |
| *qELS3-3* | IF_2_ | E3 | 3 | 126 | 124.5-126.5 | *PZE-103115618* | *PZE-103118170* | 16.11 | 0.13 | -1.27 |  | 13.64 |
| *qELS3-4* | MPH | E2 | 3 | 157 | 156.5-157.5 | *SYN15014* | *SYN32691* | 3.14 |  | 0.47 |  | 4.62 |
|  | IF_2_ | E4 | 3 | 158 | 156.5-159.5 | *SYN32691* | *PZE-103150482* | 3.09 | -0.09 | 0.33 |  | 3.06 |
|  | IF_2_ | E2 | 3 | 159 | 156.5-164.5 | *SYN32691* | *PZE-103150482* | 2.75 | -0.18 | 0.32 |  | 3.73 |
| *qELS3-5* | RIL | E1 | 3 | 167 | 165.5-170.5 | *SYN28063* | *PZE-103157755* | 2.9 | -0.24 |  |  | 3.46 |
| *qELS5-1* | RIL | E2 | 5 | 92 | 90.5-92.5 | *SYN32229* | *PZE-105093385* | 2.89 | 0.28 |  |  | 3.43 |
| *qELS5-2* | RIL | E1 | 5 | 97 | 95.5-99.5 | *PZE-105096701* | *PZE-105101729* | 2.94 | 0.24 |  |  | 3.38 |
|  | IF_2_ | E4 | 5 | 97 | 95.5-99.5 | *PZE-105096701* | *PZE-105101729* | 5.11 | 0.29 | 0.16 |  | 4.92 |
| *qELS5-3* | MPH | E2 | 5 | 111 | 109.5-111.5 | *PZE-105110168* | *PZE-105111323* | 4.77 |  | 0.07 |  | 7.17 |
| *qELS5-4* | MPH | E2 | 5 | 121 | 120.5-121.5 | *PZE-105116229* | *SYN11021* | 2.82 |  | 0.46 |  | 4.37 |
| *qELS5-5* | IF_2_ | E2 | 5 | 161 | 152.5-167.5 | *PZE-105156713* | *PZE-105165053* | 4.06 | 0.39 | 0.01 |  | 6.56 |
| *qELS6* | MPH | E4 | 6 | 111 | 102.5-121.5 | *PZE-106083873* | *PZE-106115356* | 2.56 |  | 0.23 |  | 2.02 |
|  | IF_2_ | E4 | 6 | 112 | 104.5-119.5 | *PZE-106083873* | *PZE-106115356* | 3.21 | 0.14 | 0.44 |  | 5.34 |
| *qELS7-1* | RIL | E1 | 7 | 84 | 83.5-84.5 | *PZE-107081442* | *PZE-107081254* | 7.98 | 0.4 |  |  | 9.25 |
|  | RIL | E2 | 7 | 84 | 83.5-84.5 | *PZE-107081442* | *PZE-107081254* | 7.92 | 0.47 |  |  | 9.67 |
| *qELS7-2* | IF_2_ | E4 | 7 | 150 | 140.5-152.5 | *PZE-107121485* | *PZE-107130438* | 3.76 | 0.28 | -0.03 |  | 3.61 |
| *qELS7-3* | RIL | E4 | 7 | 159 | 153.5-159 | *PZE-107130514* | *PZE-107132828* | 3.82 | 0.32 |  |  | 4.4 |
| *qELS8-1* | IF_2_ | E2 | 8 | 25 | 24.5-26.5 | *PZE-108005561* | *PZA00058.6* | 2.78 | 0.06 | 0.45 |  | 4.47 |
| *qELS8-2* | MPH | E3 | 8 | 93 | 91.5-94.5 | *SYN9237* | *PZE-108056460* | 12.31 |  | 1.19 |  | 9.47 |
| *qELS8-3* | RIL | E2 | 8 | 164 | 162.5-168.5 | *SYN23659* | *SYN15047* | 4.28 | 0.34 |  |  | 5.1 |
| *qELS10-1* | IF_2_ | E4 | 10 | 33 | 28.5-37.5 | *PZE-110007326* | *PZE-110008811* | 5.59 | 0.34 | 0.06 |  | 5.62 |
| *qELS10-2* | RIL | E3 | 10 | 48 | 47.5-49.5 | *PZE-110016607* | *PZE-110016197* | 2.99 | 0.34 |  |  | 3.45 |
| *qELS10-3* | IF_2_ | E4 | 10 | 58 | 57.5-58.5 | *PZE-110028531* | *PZE-110038731* | 3.22 | 0.06 | 0.35 |  | 3 |
| *qELS10-4* | RIL | E4 | 10 | 70 | 68.5-71.5 | *PZE-110047164* | *PZE-110049371* | 9.45 | 0.51 |  |  | 11.29 |
| *qELS10-5* | RIL | E2 | 10 | 79 | 76.5-82.5 | *PZE-110068110* | *SYN17753* | 5.18 | 0.38 |  |  | 6.45 |
| *qEDS1-1* | RIL | E3 | 1 | 18 | 17.5-20.5 | *SYN5056* | *PZE-101026314* | 3.43 | -0.5 |  |  | 3.35 |
|  | IF_2_ | E3 | 1 | 19 | 17.5-20.5 | *PZE-101026314* | *PZE-101027182* | 5.17 | -0.51 | 0.11 |  | 5.56 |
| *qEDS1-2* | IF_2_ | E2 | 1 | 56 | 55.5-56.5 | *SYN25114* | *PZE-101055771* | 27.26 | -1.7 | -0.06 |  | 15.56 |
| *qEDS1-3* | IF_2_ | E2 | 1 | 59 | 58.5-60.5 | *PZE-101058322* | *SYN36080* | 14.11 | 1.13 | -0.09 |  | 7.06 |
| *qEDS1-4* | MPH | E4 | 1 | 111 | 107.5-119.5 | *PZE-101106605* | *SYN376* | 5.94 |  | 0.12 |  | 4.47 |
| *qEDS1-5* | IF_2_ | E4 | 1 | 221 | 217.5-223.5 | *SYN22772* | *SYN11155* | 7.45 | -0.08 | 0.8 |  | 8.61 |
| *qEDS2-1* | RIL | E3 | 2 | 0 | 0-0.5 | *PZE-102017304* | *PZE-102017443* | 3.59 | 0.5 |  |  | 3.34 |
|  | IF_2_ | E3 | 2 | 2 | 0-8.5 | *PZE-102017443* | *SYN7604* | 4.2 | 0.5 | -0.01 |  | 5.13 |
|  | IF2 | E4 | 2 | 2 | 0-9.5 | *PZE-102017443* | *SYN7604* | 3.53 | 0.38 | -0.06 |  | 3.58 |
| *qEDS2-2* | RIL | E2 | 2 | 96 | 95.5-97.5 | *SYN11831* | *PZE-102112161* | 3.26 | -0.43 |  |  | 4.39 |
|  | RIL | E3 | 2 | 97 | 95.5-97.5 | *SYN11831* | *PZE-102112161* | 7.89 | -0.76 |  |  | 7.64 |
|  | MPH | E3 | 2 | 97 | 95.5-97.5 | *SYN11831* | *PZE-102112161* | 5.07 |  | 0.02 |  | 2.72 |
| *qEDS2-4* | IF_2_ | E4 | 2 | 124 | 120.5-127.5 | *PZE-102131962* | *SYN19995* | 6.28 | -0.53 | -0.04 |  | 6.32 |
| *qEDS2-5* | MPH | E4 | 2 | 155 | 151.5-158.5 | *PZE-102154251* | *SYN7501* | 3.12 |  | 0.06 |  | 2.41 |
| *qEDS3-1* | RIL | E1 | 3 | 148 | 143.5-152.5 | *PZE-103136534* | *SYN15014* | 4.74 | 0.84 |  |  | 5.94 |
| *qEDS3-2* | MPH | E2 | 3 | 169 | 165.5-170.5 | *PZE-103157755* | *SYN20833* | 3.33 |  | 0.29 |  | 6.5 |
| *qEDS3-3* | RIL | E2 | 3 | 178 | 175.5-181.5 | *PZE-103161091* | *PZE-103163529* | 3.87 | 0.46 |  |  | 5.06 |
| *qEDS4-1* | IF_2_ | E2 | 4 | 44 | 42.5-45.5 | *PZE-104031302* | *PZE-104035115* | 3.34 | 0.51 | 0.31 |  | 1.6 |
| *qEDS4-2* | IF_2_ | E4 | 4 | 64 | 63.5-64.5 | *PZE-104063573* | *PZE-104065998* | 3.56 | -0.03 | 0.52 |  | 3.46 |
| *qEDS4-3* | RIL | E2 | 4 | 68 | 67.5-68.5 | *PZE-104111457* | *PZE-104053258* | 3.53 | 0.45 |  |  | 4.79 |
| *qEDS4-4* | RIL | E3 | 4 | 146 | 138.5-151.5 | *PZE-104129635* | *PZE-104150421* | 4.1 | 0.64 |  |  | 5.41 |
| *qEDS5* | IF_2_ | E3 | 5 | 20 | 19.5-21.5 | *SYN1050* | *SYN25466* | 2.65 | -0.14 | 0.48 |  | 2.96 |
| *qEDS6-1* | RIL | E1 | 6 | 0 | 0-6.5 | *PUT-163a-94473612-4863* | *PZE-106008406* | 3.14 | -0.6 |  |  | 3.06 |
|  | RIL | E2 | 6 | 0 | 0-5.5 | *PUT-163a-94473612-4863* | *PZE-106008406* | 3.2 | -0.42 |  |  | 4.16 |
|  | RIL | E4 | 6 | 3 | 0-8.5 | *PUT-163a-94473612-4863* | *PZE-106008406* | 3.65 | -0.31 |  |  | 5.63 |
| *qEDS6-2* | RIL | E3 | 6 | 23 | 22.5-23.5 | *PZE-106025164* | *PZE-106029942* | 3.58 | -0.51 |  |  | 3.41 |
| *qEDS6-3* | RIL | E1 | 6 | 135 | 127.5-142.5 | *PZE-106115356* | *SYN11192* | 3.41 | -0.76 |  |  | 4.84 |
| *qEDS7-1* | MPH | E4 | 7 | 37 | 36.5-37.5 | *SYN19841* | *PZE-107011664* | 9.4 |  | 1.02 |  | 8.39 |
| *qEDS7-2* | MPH | E4 | 7 | 40 | 39.5-41.5 | *PZE-107012088* | *PZE-107012245* | 5.04 |  | -0.71 |  | 4.1 |
| *qEDS7-3* | MPH | E3 | 7 | 73 | 66.5-79.5 | *PZE-107057229* | *PZE-107081317* | 4.81 |  | 1.02 |  | 4.02 |
| *qEDS7-4* | IF_2_ | E2 | 7 | 111 | 110.5-111.5 | *PZE-107097026* | *PZE-107098219* | 3.07 | 0.5 | 0.15 |  | 1.4 |
|  | RIL | E2 | 7 | 112 | 111.5-116.5 | *PZE-107098286* | *PZE-107107154* | 2.5 | 0.37 |  |  | 3.24 |
| *qEDS8-1* | MPH | E3 | 8 | 90 | 88.5-90.5 | *PZE-108050016* | *SYN9237* | 14.44 |  | 1.73 |  | 9.69 |
| *qEDS8-2* | RIL | E3 | 8 | 107 | 106.5-110.5 | *PZE-108077809* | *PZE-108074750* | 4.39 | 0.57 |  |  | 4.27 |
|  | IF_2_ | E4 | 8 | 115 | 110.5-118.5 | *PZE-108092173* | *PZE-108096683* | 3.03 | 0.33 | 0.15 |  | 2.68 |
| *qEDS9-1* | RIL | E4 | 9 | 2 | 0-3.5 | *PZE-109000394* | *PZE-109001604* | 3.06 | 0.26 |  |  | 4.19 |
|  | RIL | E3 | 9 | 5 | 3.5-6.5 | *PZE-109003441* | *SYN22281* | 3.81 | 0.52 |  |  | 3.59 |
| *qEDS9-2* | RIL | E1 | 9 | 22 | 14.5-28.5 | *PZE-109008839* | *PZE-109015923* | 3 | 0.66 |  |  | 3.6 |
| *qEDS10-1* | IF_2_ | E4 | 10 | 60 | 59.5-60.5 | *PZE-110038658* | *SYN18227* | 5 | 0.4 | 0.2 |  | 4.48 |
| *qEDS10-2* | RIL | E4 | 10 | 65 | 62.5-66.5 | *PZE-110025994* | *PZE-110043216* | 2.6 | 0.24 |  |  | 3.39 |
| *qEDS10-3* | MPH | E4 | 10 | 79 | 78.5-82.5 | *PZE-110068110* | *SYN17753* | 2.91 |  | 0.54 |  | 2.32 |
|  | IF_2_ | E4 | 10 | 81 | 76.5-86.5 | *PZE-110068110* | *SYN17753* | 2.51 | 0.1 | 0.42 |  | 2.43 |
| *qCDS1-1* | IF_2_ | E2 | 1 | 56 | 54.5-57.5 | *SYN25114* | *PZE-101055771* | 7.9 | -0.33 | -0.03 |  | 12.73 |
|  | IF_2_ | E3 | 1 | 56 | 54.5-58.5 | *SYN25114* | *PZE-101055771* | 4.34 | -0.32 | 0.08 |  | 5.31 |
| *qCDS1-2* | RIL | E1 | 1 | 60 | 58.5-61.5 | *PZE-101058322* | *SYN36080* | 4.22 | -0.4 |  |  | 6.07 |
|  | IF_2_ | E4 | 1 | 62 | 60.5-65.5 | *SYN13385* | *SYN37775* | 3.19 | -0.2 | -0.07 |  | 3.43 |
|  | RIL | E3 | 1 | 63 | 60.5-65.5 | *SYN13385* | *SYN37775* | 4.07 | -0.42 |  |  | 4.38 |
| *qCDS1-3* | MPH | E4 | 1 | 111 | 108.5-118.5 | *PZE-101106605* | *SYN376* | 4.68 |  | -0.07 |  | 6.22 |
| *qCDS1-4* | IF_2_ | E3 | 1 | 173 | 171.5-176.5 | *PZE-101187496* | *PZE-101196838* | 2.97 | 0.28 | 0 |  | 3.69 |
| *qCDS1-5* | IF_2_ | E4 | 1 | 217 | 210.5-220.5 | *SYN22772* | *SYN11155* | 2.6 | -0.07 | 0.22 |  | 2.67 |
| *qCDS2-1* | RIL | E3 | 2 | 0 | 0-0.5 | *PZE-102017304* | *PZE-102017443* | 4.05 | 0.4 |  |  | 3.86 |
| *qCDS2-2* | RIL | E3 | 2 | 94 | 93.5-95.5 | *SYN35922* | *PZE-102111018* | 5.26 | -0.46 |  |  | 5.24 |
| *qCDS3-1* | IF_2_ | E4 | 3 | 10 | 2.5-17.5 | *PZE-103001968* | *SYN25628* | 2.73 | -0.13 | -0.23 |  | 2.97 |
| *qCDS3-2* | MPH | E2 | 3 | 26 | 21.5-29.5 | *SYN10329* | *SYN7905* | 2.55 |  | -0.19 |  | 4.75 |
| *qCDS4-1* | IF_2_ | E2 | 4 | 47 | 46.5-47.5 | *PZE-104033683* | *PZE-104033489* | 4.97 | 0.25 | 0.13 |  | 7.78 |
| *qCDS4-2* | RIL | E1 | 4 | 53 | 51.5-53.5 | *SYN25530* | *PZE-104050148* | 2.62 | 0.31 |  |  | 3.7 |
| *qCDS4-3* | IF_2_ | E3 | 4 | 60 | 59.5-60.5 | *PZE-104048874* | *PZE-104049163* | 5.65 | 0.37 | -0.06 |  | 6.94 |
| *qCDS4-4* | RIL | E2 | 4 | 68 | 67.5-68.5 | *PZE-104111457* | *PZE-104053258* | 3.7 | 0.4 |  |  | 4.65 |
| *qCDS4-5* | MPH | E3 | 4 | 143 | 134.5-152.5 | *PZE-104129635* | *PZE-104150421* | 2.91 |  | 0.15 |  | 4.8 |
| *qCDS4-6* | RIL | E3 | 4 | 158 | 156.5-163.5 | *SYN7941* | *PZE-104154610* | 3.57 | 0.4 |  |  | 3.94 |
| *qCDS5* | IF_2_ | E4 | 5 | 118 | 117.5-119.5 | *PZE-105115759* | *PZA00987.1* | 3.11 | -0.02 | -0.29 |  | 3.34 |
| *qCDS6-1* | RIL | E4 | 6 | 3 | 0-8.5 | *PUT-163a-94473612-4863* | *PZE-106008406* | 2.75 | -0.17 |  |  | 3.99 |
|  | RIL | E2 | 6 | 9 | 8.5-9.5 | *PZE-106008406* | *PZE-106020499* | 7.93 | -0.58 |  |  | 9.96 |
| *qCDS6-2* | IF_2_ | E3 | 6 | 13 | 10.5-14.5 | *SYN36832* | *PZE-106012837* | 7.54 | -0.44 | 0.1 |  | 9.58 |
| *qCDS6-3* | RIL | E3 | 6 | 23 | 22.5-23.5 | *PZE-106025164* | *PZE-106029942* | 7.02 | -0.54 |  |  | 7.01 |
| *qCDS6-4* | RIL | E2 | 6 | 64 | 58.5-65.5 | *PZE-106043310* | *PZE-106055082* | 3.07 | 0.36 |  |  | 3.76 |
| *qCDS6-5* | IF_2_ | E4 | 6 | 114 | 104.5-122.5 | *PZE-106083873* | *PZE-106115356* | 2.53 | 0.15 | 0.29 |  | 4.72 |
| *qCDS7-1* | MPH | E4 | 7 | 37 | 35.5-37.5 | *SYN19841* | *PZE-107011664* | 3.36 |  | 0.35 |  | 5.06 |
| *qCDS7-2* | RIL | E2 | 7 | 84 | 83.5-84.5 | *PZE-107081442* | *PZE-107081254* | 3.53 | 0.38 |  |  | 4.26 |
| *qCDS7-3* | IF_2_ | E2 | 7 | 102 | 101.5-103.5 | *PZE-107094078* | *PZE-107094398* | 4.08 | 0.23 | 0.08 |  | 6.44 |
| *qCDS7-4* | IF_2_ | E3 | 7 | 110 | 106.5-111.5 | *PZE-107095878* | *PZE-107097026* | 2.78 | 0.26 | 0.11 |  | 3.41 |
| *qCDS8-1* | RIL | E3 | 8 | 46 | 42.5-51.5 | *PZE-108009621* | *PZE-108012113* | 5.72 | 0.48 |  |  | 5.6 |
| *qCDS8-2* | RIL | E4 | 8 | 102 | 98.5-106.5 | *SYN21795* | *PZE-108077809* | 2.99 | 0.17 |  |  | 3.93 |
|  | IF_2_ | E3 | 8 | 106 | 103.5-107.5 | *SYN21795* | *PZE-108077809* | 3.3 | 0.23 | 0.3 |  | 4.02 |
| *qCDS9-1* | RIL | E3 | 9 | 4 | 3.5-6.5 | *PZE-109001604* | *PZE-109003441* | 5.77 | 0.49 |  |  | 5.86 |
| *qCDS9-2* | RIL | E1 | 9 | 29 | 26.5-31.5 | *SYN6084* | *PZE-109016787* | 3.65 | 0.38 |  |  | 5.41 |
| *qCDS10-1* | MPH | E4 | 10 | 71 | 69.5-71.5 | *PZE-110047164* | *PZE-110049371* | 3.1 |  | 0.29 |  | 4.11 |
| *qCDS10-2* | IF_2_ | E4 | 10 | 82 | 78.5-83.5 | *PZE-110068110* | *SYN17753* | 2.96 | 0.15 | 0.2 |  | 3.15 |
|  | RIL | E2 | 10 | 84 | 82.5-87.5 | *PZE-110074914* | *PZE-110079903* | 2.79 | 0.34 |  |  | 3.4 |
|  | RIL | E4 | 10 | 85 | 82.5-88.5 | *PZE-110074914* | *PZE-110079903* | 4.23 | 0.21 |  |  | 5.91 |
| *qCDS10-3* | IF_2_ | E4 | 10 | 94 | 89.5-96.5 | *PZE-110082048* | *PZA02663.1* | 2.98 | 0.16 | 0.22 |  | 3.46 |
| *qRNS1-1* | RIL | E2 | 1 | 62 | 60.5-64.5 | *SYN13385* | *SYN37775* | 3.87 | -0.19 |  |  | 3.75 |
|  | RIL | E4 | 1 | 62 | 60.5-65.5 | *SYN13385* | *SYN37775* | 3 | -0.2 |  |  | 3.57 |
|  | RIL | E1 | 1 | 64 | 61.5-67.5 | *SYN13385* | *SYN37775* | 4.63 | -0.2 |  |  | 4.34 |
| *qRNS1-2* | IF_2_ | E4 | 1 | 80 | 78.5-83.5 | *PZE-101071898* | *SYN3987* | 3.61 | 0.1 | 0.34 |  | 3.3 |
| *qRNS1-3* | IF_2_ | E4 | 1 | 150 | 145.5-154.5 | *SYN376* | *PZE-101173330* | 2.96 | -0.23 | -0.18 |  | 3.09 |
| *qRNS1-4* | IF_2_ | E2 | 1 | 217 | 211.5-222.5 | *SYN22772* | *SYN11155* | 4.07 | -0.32 | 0.09 |  | 6.42 |
|  | MPH | E2 | 1 | 219 | 210.5-224.5 | *SYN22772* | *SYN11155* | 3.63 |  | -0.08 |  | 6.95 |
| *qRNS1-5* | RIL | E1 | 1 | 225 | 221.5-228.5 | *SYN11155* | *SYN12789* | 4.49 | -0.2 |  |  | 4.02 |
|  | MPH | E4 | 1 | 228 | 224.5-234.5 | *SYN11155* | *SYN12789* | 2.66 |  | -0.11 |  | 4.08 |
| *qRNS2-1* | RIL | E1 | 2 | 102 | 100.5-102.5 | *PZE-102113766* | *SYN27293* | 4.86 | -0.2 |  |  | 4.22 |
| *qRNS2-2* | IF_2_ | E4 | 2 | 198 | 190.5-203.5 | *PZE-102186160* | *SYN14631* | 3.15 | -0.22 | 0.08 |  | 2.88 |
| *qRNS2-3* | IF_2_ | E3 | 2 | 212 | 206.5-216 | *PZE-102189664* | *PZE-102193611* | 2.55 | -0.2 | 0.22 |  | 4.19 |
| *qRNS3-1* | RIL | E1 | 3 | 57 | 55.5-59.5 | *PZE-103029035* | *PZE-103032109* | 6.38 | -0.23 |  |  | 5.68 |
|  | IF_2_ | E4 | 3 | 58 | 56.5-60.5 | *PZE-103029035* | *PZE-103032109* | 5.89 | -0.32 | 0.18 |  | 5.16 |
|  | IF_2_ | E2 | 3 | 59 | 56.5-60.5 | *PZE-103029035* | *PZE-103032109* | 3.97 | -0.35 | -0.02 |  | 6.5 |
|  | IF_2_ | E3 | 3 | 59 | 56.5-62.5 | *PZE-103029035* | *PZE-103032109* | 2.79 | -0.23 | -0.02 |  | 3.33 |
|  | RIL | E3 | 3 | 62 | 59.5-64.5 | *PZE-103032109* | *PZE-103033919* | 3.43 | -0.25 |  |  | 4.36 |
|  | RIL | E2 | 3 | 64 | 62.5-64.5 | *PZE-103033919* | *PZE-103036266* | 8.06 | -0.28 |  |  | 7.83 |
| *qRNS3-2* | RIL | E4 | 3 | 67 | 64.5-68.5 | *PZE-103037508* | *PZA02255.2* | 3.14 | -0.21 |  |  | 3.72 |
| *qRNS3-3* | IF_2_ | E3 | 3 | 77 | 76.5-77.5 | *PZE-103049396* | *PZE-103050010* | 2.84 | 0.08 | 0.29 |  | 3.36 |
| *qRNS3-4* | RIL | E3 | 3 | 171 | 168.5-173.5 | *SYN20833* | *PZE-103160158* | 4.07 | 0.28 |  |  | 5.21 |
|  | IF_2_ | E4 | 3 | 172 | 170.5-175.5 | *SYN20833* | *PZE-103160158* | 6.46 | 0.31 | 0.07 |  | 5.79 |
| *qRNS3-5* | RIL | E1 | 3 | 176 | 175.5-177.5 | *PZE-103161091* | *PZE-103163529* | 5.99 | 0.22 |  |  | 5.2 |
|  | RIL | E2 | 3 | 176 | 174.5-176.5 | *PZE-103161091* | *PZE-103163529* | 8.31 | 0.28 |  |  | 7.9 |
|  | RIL | E4 | 3 | 176 | 174.5-177.5 | *PZE-103161091* | *PZE-103163529* | 3.99 | 0.23 |  |  | 4.59 |
|  | MPH | E3 | 3 | 176 | 174.5-177.5 | *PZE-103161091* | *PZE-103163529* | 3.05 |  | -0.26 |  | 5.28 |
| *qRNS3-6* | IF_2_ | E4 | 3 | 207 | 202.5-212.5 | *ZM012337-0431* | *PZE-103182712* | 4.53 | -0.26 | -0.04 |  | 3.93 |
| *qRNS4* | IF_2_ | E3 | 4 | 8 | 5.5-12.5 | *PZE-104012412* | *SYN8509* | 2.72 | 0.01 | -0.34 |  | 4.09 |
| *qRNS5* | RIL | E3 | 5 | 140 | 139.5-141.5 | *PZE-105132778* | *PZE-105132845* | 3.57 | 0.26 |  |  | 4.49 |
|  | IF_2_ | E2 | 5 | 140 | 139.5-141.5 | *PZE-105132778* | *PZE-105132845* | 3.06 | 0.29 | -0.09 |  | 4.92 |
|  | IF_2_ | E3 | 5 | 140 | 139.5-141.5 | *PZE-105132778* | *PZE-105132845* | 5.27 | 0.3 | -0.1 |  | 6.29 |
| *qRNS6* | IF_2_ | E2 | 6 | 153 | 149.5-153 | *SYN38610* | *PZE-106129664* | 2.54 | -0.19 | 0.26 |  | 3.75 |
| *qRNS7-1* | IF_2_ | E4 | 7 | 3 | 0-4.5 | *PZA01426.1* | *SYN10723* | 2.73 | 0.1 | 0.28 |  | 2.79 |
|  | MPH | E2 | 7 | 4 | 0-17.5 | *PZA01426.1* | *SYN10723* | 2.63 |  | 0.21 |  | 4.24 |
| *qRNS7-2* | RIL | E4 | 7 | 59 | 58.5-59.5 | *PZE-107033682* | *PZE-107030398* | 2.75 | -0.19 |  |  | 3.16 |
|  | RIL | E2 | 7 | 61 | 59.5-65.5 | *PZE-107049384* | *PZE-107057229* | 3.31 | -0.18 |  |  | 3.1 |
| *qRNS7-3* | IF_2_ | E4 | 7 | 153 | 150.5-153.5 | *PZE-107130789* | *PZE-107130514* | 3.45 | -0.24 | -0.02 |  | 2.94 |
|  | RIL | E3 | 7 | 158 | 153.5-159 | *PZE-107130514* | *PZE-107132828* | 3.37 | -0.26 |  |  | 4.57 |
|  | RIL | E1 | 7 | 159 | 155.5-159 | *PZE-107130514* | *PZE-107132828* | 5.56 | -0.22 |  |  | 5.02 |
|  | RIL | E2 | 7 | 159 | 155.5-159 | *PZE-107130514* | *PZE-107132828* | 3.45 | -0.18 |  |  | 3.29 |
|  | RIL | E4 | 7 | 159 | 154.5-159 | *PZE-107130514* | *PZE-107132828* | 2.98 | -0.2 |  |  | 3.52 |
|  | IF_2_ | E2 | 7 | 159 | 155.5-159 | *PZE-107130514* | *PZE-107132828* | 3 | -0.28 | 0.01 |  | 4.74 |
| *qRNS8* | IF_2_ | E3 | 8 | 106 | 103.5-107.5 | *SYN21795* | *PZE-108077809* | 3.13 | 0.19 | 0.24 |  | 3.72 |
| *qRNS9-1* | RIL | E1 | 9 | 27 | 26.5-31.5 | *PZE-109015923* | *SYN6084* | 5.55 | 0.22 |  |  | 5.01 |
|  | RIL | E4 | 9 | 27 | 26.5-30.5 | *PZE-109015923* | *SYN6084* | 4.4 | 0.24 |  |  | 5.19 |
|  | RIL | E2 | 9 | 28 | 26.5-31.5 | *PZE-109015923* | *SYN6084* | 2.88 | 0.16 |  |  | 2.67 |
| *qRNS9-2* | MPH | E4 | 9 | 45 | 40.5-50.5 | *PZE-109019784* | *PZE-109023988* | 4.39 |  | 0.16 |  | 6.7 |
| *qRNS9-3* | IF_2_ | E4 | 9 | 69 | 68.5-70.5 | *PZB00235.1* | *PZE-109035290* | 7.31 | -0.33 | -0.02 |  | 6.25 |
|  | IF_2_ | E2 | 9 | 70 | 68.5-70.5 | *PZE-109035290* | *PZE-109035200* | 3.07 | -0.28 | -0.03 |  | 4.77 |
| *qRNS9-4* | IF_2_ | E3 | 9 | 88 | 87.5-89.5 | *PZE-109055211* | *PZE-109055660* | 6.84 | -0.35 | 0.04 |  | 8.35 |
| *qRNS9-5* | RIL | E2 | 9 | 93 | 91.5-95.5 | *PZE-109056180* | *PZE-109056596* | 9.92 | -0.31 |  |  | 9.98 |
|  | RIL | E1 | 9 | 94 | 91.5-95.5 | *PZE-109056596* | *PZE-109057210* | 8.01 | -0.26 |  |  | 7.2 |
|  | RIL | E3 | 9 | 97 | 95.5-99.5 | *PZE-109057210* | *PZB01899.2* | 4.79 | -0.3 |  |  | 5.97 |
|  | RIL | E4 | 9 | 97 | 95.5-98.5 | *PZE-109057210* | *PZB01899.2* | 6.67 | -0.3 |  |  | 7.86 |
| *qRNS10-1* | MPH | E4 | 10 | 55 | 54.5-55.5 | *PZE-110020162* | *PZE-110020077* | 3.71 |  | 0.02 |  | 4.84 |
| *qRNS10-2* | RIL | E3 | 10 | 59 | 58.5-59.5 | *PZE-110038731* | *PZE-110038481* | 3.54 | 0.26 |  |  | 4.46 |
| *qRNS10-3* | IF_2_ | E3 | 10 | 60 | 59.5-60.5 | *PZE-110038658* | *SYN18227* | 6.54 | 0.34 | -0.03 |  | 7.77 |
| *qRNS10-4* | RIL | E2 | 10 | 62 | 60.5-65.5 | *PZE-110025994* | *PZE-110043216* | 3.07 | 0.17 |  |  | 2.9 |
| *qRNS10-5* | RIL | E1 | 10 | 75 | 74.5-75.5 | *PZE-110054411* | *PZE-110053918* | 3.29 | 0.16 |  |  | 2.82 |
|  | IF_2_ | E2 | 10 | 75 | 74.5-75.5 | *PZE-110054411* | *PZE-110053918* | 3.04 | 0.27 | -0.1 |  | 4.66 |
|  | MPH | E2 | 10 | 75 | 74.5-78.5 | *PZE-110054411* | *PZE-110053918* | 2.69 |  | -0.1 |  | 4.47 |
| *qKNPRS1-1* | RIL | E3 | 1 | 31 | 29.5-34.5 | *PZE-101032249* | *PZE-101033801* | 2.65 | -1.02 |  |  | 4.19 |
|  | IF_2_ | E3 | 1 | 33 | 32.5-34.5 | *PZE-101033801* | *PZE-101035008* | 3.59 | -0.85 | 0.38 |  | 4.68 |
| *qKNPRS1-2* | IF_2_ | E2 | 1 | 67 | 62.5-68.5 | *SYN37775* | *SYN29479* | 2.99 | -0.83 | 0.71 |  | 4.81 |
| *qKNPRS1-3* | IF_2_ | E3 | 1 | 156 | 152.5-159.5 | *PZE-101173330* | *SYN2411* | 2.93 | -0.53 | 0.76 |  | 3.6 |
| *qKNPRS1-4* | IF_2_ | E3 | 1 | 176 | 172.5-181.5 | *PZE-101187496* | *PZE-101196838* | 5.61 | -1.07 | 0.6 |  | 8.02 |
| *qKNPRS2-1* | IF_2_ | E3 | 2 | 59 | 57.5-59.5 | *PZE-102063039* | *PZE-102065424* | 4.29 | -0.17 | 1.42 |  | 5.62 |
| *qKNPRS2-2* | MPH | E2 | 2 | 70 | 69.5-71.5 | *PZE-102077128* | *PZE-102077234* | 4.2 |  | 1.96 |  | 2.1 |
| *qKNPRS2-3* | IF_2_ | E4 | 2 | 75 | 74.5-75.5 | *PZE-102080336* | *SYN27612* | 3.91 | -0.15 | 1.42 |  | 5.22 |
| *qKNPRS2-4* | MPH | E2 | 2 | 95 | 94.5-95.5 | *PZE-102111018* | *PZE-102110333* | 24.9 |  | -5.31 |  | 15.66 |
| *qKNPRS2-5* | MPH | E2 | 2 | 98 | 97.5-99.5 | *SYN34894* | *SYN13599* | 8.32 |  | 2.74 |  | 4.16 |
| *qKNPRS2-6* | MPH | E2 | 2 | 116 | 114.5-116.5 | *PZE-102127480* | *PZE-102129070* | 5.1 |  | 2.21 |  | 2.73 |
| *qKNPRS3* | RIL | E4 | 3 | 177 | 175.5-181.5 | *PZE-103161091* | *PZE-103163529* | 4.4 | 1.03 |  |  | 5.75 |
|  | RIL | E2 | 3 | 178 | 176.5-179.5 | *PZE-103161091* | *PZE-103163529* | 8.34 | 1.24 |  |  | 10.24 |
| *qKNPRS4-1* | RIL | E2 | 4 | 11 | 5.5-16.5 | *PZE-104012412* | *SYN8509* | 3.3 | -0.82 |  |  | 4.54 |
| *qKNPRS4-2* | MPH | E4 | 4 | 39 | 37.5-39.5 | *PZE-104029222* | *PZE-104029384* | 3.18 |  | 1.7 |  | 4.47 |
| *qKNPRS4-3* | IF_2_ | E4 | 4 | 51 | 48.5-52.5 | *PZE-104042305* | *SYN25530* | 2.59 | -0.52 | 0.82 |  | 3.17 |
| *qKNPRS4-4* | IF_2_ | E2 | 4 | 137 | 129.5-143.5 | *PZE-104103734* | *PZE-104129635* | 3.5 | -1.1 | 0.23 |  | 5.93 |
| *qKNPRS5-1* | MPH | E3 | 5 | 70 | 68.5-70.5 | *PZE-105044182* | *SYN11795* | 6.33 |  | 0.21 |  | 9.69 |
| *qKNPRS5-2* | RIL | E4 | 5 | 96 | 93.5-96.5 | *PZE-105098019* | *PZE-105096701* | 4.04 | 0.98 |  |  | 5.14 |
| *qKNPRS5-3* | RIL | E2 | 5 | 103 | 102.5-103.5 | *PZE-105102631* | *SYN38374* | 3.67 | 0.81 |  |  | 4.43 |
| *qKNPRS6* | RIL | E4 | 6 | 104 | 100.5-104.5 | *PZE-106083588* | *PZE-106080884* | 3.18 | -0.86 |  |  | 4 |
|  | IF_2_ | E4 | 6 | 104 | 103.5-104.5 | *PZE-106083588* | *PZE-106080884* | 3.69 | 0.04 | 1.41 |  | 4.92 |
|  | MPH | E3 | 6 | 104 | 103.5-104.5 | *PZE-106083588* | *PZE-106080884* | 4.34 |  | 1.66 |  | 6.36 |
|  | IF_2_ | E3 | 6 | 105 | 104.5-105.5 | *PZE-106080884* | *PZE-106083873* | 3.06 | -0.01 | 1.19 |  | 3.92 |
|  | MPH | E4 | 6 | 112 | 104.5-119.5 | *PZE-106083873* | *PZE-106115356* | 4.67 |  | 1.25 |  | 9.18 |
| *qKNPRS7-1* | RIL | E4 | 7 | 48 | 41.5-53.5 | *PZE-107012245* | *SYN24186* | 3.58 | -1 |  |  | 5.34 |
| *qKNPRS7-2* | MPH | E3 | 7 | 86 | 84.5-86.5 | *PZE-107081254* | *PZE-107084740* | 2.85 |  | 1.25 |  | 4.28 |
|  | IF_2_ | E3 | 7 | 87 | 84.5-88.5 | *PZE-107084740* | *PZE-107086184* | 3.22 | 0.12 | 1.17 |  | 4.1 |
| *qKNPRS7-3* | IF_2_ | E4 | 7 | 103 | 102.5-104.5 | *PZE-107094398* | *PZE-107094385* | 3.75 | -1 | 0.26 |  | 4.82 |
| *qKNPRS8* | IF_2_ | E2 | 8 | 9 | 4.5-10.5 | *PZE-108002130* | *PZE-108002532* | 2.91 | 1 | 0.24 |  | 5.14 |
| *qKNPRS9* | IF_2_ | E3 | 9 | 70 | 68.5-71.5 | *PZE-109035290* | *PZE-109035200* | 2.55 | -0.52 | 0.71 |  | 3.08 |
| *qKNPRS10-1* | IF_2_ | E2 | 10 | 29 | 20.5-31.5 | *PZE-110007326* | *PZE-110008811* | 2.81 | 0.51 | 1.15 |  | 4.36 |
| *qKNPRS10-2* | IF_2_ | E4 | 10 | 65 | 61.5-66.5 | *PZE-110025994* | *PZE-110043216* | 5.26 | 1.14 | 0.39 |  | 6.81 |
| *qKNPRS10-3* | IF_2_ | E4 | 10 | 125 | 118.5-128.5 | *PZE-110095199* | *PZE-110103156* | 3.11 | -0.91 | 0.4 |  | 4.26 |
| *qKWPES1* | RIL | E1 | 1 | 178 | 174.5-181.5 | *PZE-101187496* | *PZE-101196838* | 4.67 | -3.01 |  |  | 5.8 |
| *qKWPES2-1* | MPH | E3 | 2 | 16 | 7.5-21.5 | *PZE-102017443* | *SYN7604* | 2.56 |  | 4.92 |  | 3.45 |
| *qKWPES2-2* | IF_2_ | E3 | 2 | 50 | 48.5-50.5 | *PZE-102056669* | *SYN28948* | 7.14 | -1.31 | 10.27 |  | 6.28 |
| *qKWPES2-3* | MPH | E3 | 2 | 62 | 61.5-62.5 | *PZA02450.1* | *SYN21924* | 4.85 |  | 11.1 |  | 7.91 |
| *qKWPES2-4* | RIL | E3 | 2 | 95 | 93.5-95.5 | *PZE-102111018* | *PZE-102110333* | 2.71 | -3.53 |  |  | 4.66 |
| *qKWPES2-5* | IF_2_ | E4 | 2 | 111 | 109.5-112.5 | *PZE-102119200* | *PZE-102125779* | 3.09 | -0.14 | 4.01 |  | 1.87 |
| *qKWPES2-6* | IF_2_ | E2 | 2 | 114 | 113.5-114.5 | *PZA03661.3* | *PZE-102127480* | 3.78 | -0.73 | 5.39 |  | 3.39 |
| *qKWPES2-7* | IF_2_ | E4 | 2 | 126 | 122.5-127.5 | *SYN19995* | *SYN5428* | 6 | -4.11 | -1.19 |  | 3.5 |
| *qKWPES3-1* | MPH | E2 | 3 | 166 | 165.5-168.5 | *SYN28063* | *PZE-103157755* | 4.77 |  | -1.49 |  | 11.97 |
| *qKWPES3-2* | RIL | E2 | 3 | 177 | 174.5-178.5 | *PZE-103161091* | *PZE-103163529* | 4.53 | 2.34 |  |  | 8.01 |
|  | RIL | E1 | 3 | 178 | 176.5-180.5 | *PZE-103161091* | *PZE-103163529* | 5.05 | 2.96 |  |  | 5.63 |
| *qKWPES3-3* | MPH | E3 | 3 | 217 | 216.5-217 | *PZE-103183391* | *PZE-103183701* | 2.86 |  | 1.76 |  | 4.03 |
| *qKWPES4-1* | MPH | E4 | 4 | 39 | 37.5-39.5 | *PZE-104029222* | *PZE-104029384* | 2.81 |  | 6.22 |  | 10.04 |
| *qKWPES4-2* | IF_2_ | E4 | 4 | 61 | 60.5-62.5 | *PZE-104050647* | *SYN11159* | 16.92 | 6.76 | 2.53 |  | 10.53 |
| *qKWPES4-3* | IF_2_ | E4 | 4 | 70 | 69.5-70.5 | *PZE-104056726* | *PZE-104112105* | 27.37 | -8.94 | 0.76 |  | 18.72 |
| *qKWPES4-4* | RIL | E1 | 4 | 110 | 109.5-114.5 | *SYN606* | *PZE-104097453* | 3.66 | -2.57 |  |  | 4.21 |
| *qKWPES5* | RIL | E1 | 5 | 82 | 79.5-84.5 | *PZE-105067537* | *PZE-105078059* | 4.39 | 2.81 |  |  | 5.07 |
| *qKWPES6-1* | RIL | E1 | 6 | 104 | 103.5-105.5 | *PZE-106083588* | *PZE-106080884* | 5.08 | -3 |  |  | 5.79 |
|  | IF_2_ | E4 | 6 | 104 | 103.5-104.5 | *PZE-106083588* | *PZE-106080884* | 3.23 | 0.09 | 4.13 |  | 1.95 |
|  | MPH | E3 | 6 | 104 | 103.5-104.5 | *PZE-106083588* | *PZE-106080884* | 3.45 |  | 8.95 |  | 5.27 |
| *qKWPES6-2* | IF_2_ | E3 | 6 | 111 | 104.5-116.5 | *PZE-106083873* | *PZE-106115356* | 4.6 | -0.44 | 10.63 |  | 6.95 |
| *qKWPES7-1* | IF_2_ | E3 | 7 | 49 | 43.5-51.5 | *PZE-107012245* | *SYN24186* | 2.84 | -0.44 | 7.31 |  | 3.17 |
| *qKWPES7-2* | IF_2_ | E2 | 7 | 56 | 53.5-58.5 | *PZE-107019133* | *PZE-107033682* | 2.73 | -0.81 | 4.55 |  | 2.18 |
|  | IF_2_ | E4 | 7 | 57 | 54.5-59.5 | *PZE-107019133* | *PZE-107033682* | 4.76 | -3.77 | 2.42 |  | 3.13 |
| *qKWPES7-3* | MPH | E3 | 7 | 71 | 64.5-76.5 | *PZE-107057229* | *PZE-107081317* | 4.63 |  | 12.76 |  | 10.19 |
| *qKWPES7-4* | IF_2_ | E3 | 7 | 94 | 92.5-94.5 | *PZE-107088270* | *PZE-107088998* | 2.61 | -1.09 | 6.22 |  | 2.28 |
| *qKWPES8-1* | MPH | E4 | 8 | 101 | 99.5-105.5 | *PZE-108062173* | *SYN21795* | 2.63 |  | 3.91 |  | 7.33 |
| *qKWPES8-2* | IF_2_ | E4 | 8 | 115 | 111.5-117.5 | *PZE-108092173* | *PZE-108096683* | 5.43 | 3.77 | 0.68 |  | 3.12 |
| *qKWPES8-3* | MPH | E3 | 8 | 134 | 132.5-138.5 | *SYN13209* | *SYN30185* | 5.6 |  | -5.4 |  | 8.69 |
|  | IF_2_ | E3 | 8 | 140 | 137.5-140.5 | *SYN30185* | *PZE-108115325* | 2.59 | -4.26 | -1.77 |  | 2.29 |
| *qKWPES9-1* | IF_2_ | E3 | 9 | 52 | 47.5-53.5 | *PZE-109019784* | *PZE-109023988* | 5.82 | -5.63 | 3.6 |  | 4.89 |
|  | RIL | E1 | 9 | 53 | 52.5-55.5 | *PZE-109023988* | *PZE-109026030* | 2.57 | 2.1 |  |  | 2.84 |
| *qKWPES9-2* | IF_2_ | E2 | 9 | 73 | 72.5-73.5 | *PZE-109038841* | *PZE-109047418* | 13.93 | 6.87 | 0.66 |  | 10.18 |
| *qKWPES9-3* | IF_2_ | E4 | 9 | 80 | 79.5-80.5 | *PZE-109049656* | *SYN5187* | 3.24 | -0.56 | 4.25 |  | 2.26 |
| *qKWPES9-4* | IF_2_ | E2 | 9 | 85 | 83.5-85.5 | *PZA03595.2* | *PZA03596.1* | 24.61 | -9.45 | 0.36 |  | 20.13 |
| *qKWPES9-5* | IF_2_ | E2 | 9 | 126 | 123.5-129.5 | *PZE-109089936* | *PZE-109092637* | 2.74 | 1.47 | 3.77 |  | 1.74 |
| *qKWPES10-1* | IF_2_ | E4 | 10 | 60 | 59.5-60.5 | *PZE-110038658* | *SYN18227* | 6.52 | 3.94 | 1.4 |  | 3.76 |
| *qKWPES10-2* | MPH | E4 | 10 | 65 | 62.5-66.5 | *PZE-110025994* | *PZE-110043216* | 3.17 |  | 5.92 |  | 9.53 |
| *qKWPES10-3* | IF_2_ | E4 | 10 | 80 | 78.5-82.5 | *PZE-110068110* | *SYN17753* | 3.85 | 0.01 | 4.66 |  | 2.48 |
| *qRKPS1-1* | MPH | E3 | 1 | 44 | 41.5-45.5 | *SYN23119* | *PZE-101043670* | 4.32 |  | -0.0046 |  | 5.53 |
| *qRKPS1-2* | IF2 | E3 | 1 | 168 | 163.5-171.5 | *SYN31271* | *PZE-101184757* | 4.99 | -0.01 | 0.0046 |  | 5.22 |
|  | RIL | E1 | 1 | 171 | 168.5-172.5 | *PZE-101184757* | *SYN25826* | 4.12 | -0.03 |  |  | 4.01 |
| *qRKPS2-1* | MPH | E2 | 2 | 94 | 93.5-94.5 | *SYN35922* | *PZE-102111018* | 3.26 |  | -0.02 |  | 4.08 |
| *qRKPS2-2* | RIL | E3 | 2 | 133 | 132.5-135.5 | *PZE-102145703* | *PZE-102146058* | 4.06 | 0.01 |  |  | 5.09 |
| *qRKPS2-3* | IF2 | E4 | 2 | 165 | 162.5-168.5 | *PZE-102173306* | *PZE-102175026* | 4.24 | -0.0021 | -0.01 |  | 4.91 |
| *qRKPS2-4* | MPH | E2 | 2 | 192 | 189.5-195.5 | *SYN8348* | *PZE-102186160* | 2.97 |  | 0.02 |  | 4.4 |
| *qRKPS3-1* | MPH | E2 | 3 | 43 | 34.5-45.5 | *SYN7905* | *PZE-103018221* | 3.06 |  | 0.02 |  | 3.88 |
| *qRKPS3-2* | MPH | E2 | 3 | 143 | 140.5-144.5 | *PZE-103132112* | *PZE-103136534* | 4.99 |  | -0.01 |  | 6.28 |
|  | RIL | E1 | 3 | 147 | 143.5-150.5 | *PZE-103136534* | *SYN15014* | 9.76 | 0.04 |  |  | 11.32 |
|  | MPH | E4 | 3 | 152 | 145.5-156.5 | *PZE-103136534* | *SYN15014* | 2.72 |  | -0.01 |  | 5.36 |
| *qRKPS3-3* | MPH | E2 | 3 | 171 | 168.5-173.5 | *SYN20833* | *PZE-103160158* | 5.82 |  | -0.0014 |  | 7.4 |
|  | MPH | E3 | 3 | 172 | 168.5-174.5 | *SYN20833* | *PZE-103160158* | 4.91 |  | -0.0025 |  | 6.76 |
|  | RIL | E2 | 3 | 174 | 170.5-175.5 | *SYN20833* | *PZE-103160158* | 9.23 | 0.03 |  |  | 12.04 |
|  | RIL | E4 | 3 | 174 | 170.5-175.5 | *SYN20833* | *PZE-103160158* | 4.25 | 0.02 |  |  | 6.09 |
|  | MPH | E4 | 3 | 174 | 170.5-177.5 | *SYN20833* | *PZE-103160158* | 2.54 |  | -0.01 |  | 3.89 |
| *qRKPS4-1* | IF2 | E2 | 4 | 49 | 48.5-49.5 | *PZE-104033489* | *PZE-104041535* | 2.53 | -0.01 | 0.0021 |  | 5.8 |
|  | IF2 | E4 | 4 | 50 | 49.5-50.5 | *PZE-104041818* | *PZE-104042305* | 6.05 | -0.01 | 0.0030 |  | 7.06 |
| *qRKPS4-2* | RIL | E4 | 4 | 53 | 51.5-53.5 | *SYN25530* | *PZE-104050148* | 3.67 | -0.02 |  |  | 5.14 |
|  | IF2 | E3 | 4 | 53 | 51.5-53.5 | *SYN25530* | *PZE-104050148* | 8.15 | -0.01 | 0.0004 |  | 8.75 |
| *qRKPS4-3* | RIL | E2 | 4 | 55 | 53.5-56.5 | *PZE-104051877* | *PZE-104050391* | 6.24 | -0.02 |  |  | 7.75 |
|  | MPH | E2 | 4 | 55 | 53.5-56.5 | *PZE-104051877* | *PZE-104050391* | 8.67 |  | 0.0023 |  | 11.19 |
|  | MPH | E4 | 4 | 55 | 53.5-56.5 | *PZE-104051877* | *PZE-104050391* | 3.43 |  | 0.01 |  | 5.2 |
| *qRKPS4-4* | RIL | E3 | 4 | 57 | 56.5-57.5 | *PZE-104048257* | *SYN1338* | 3.05 | -0.01 |  |  | 3.7 |
| *qRKPS4-5* | RIL | E1 | 4 | 60 | 58.5-60.5 | *PZE-104048874* | *PZE-104049163* | 3.46 | -0.02 |  |  | 3.26 |
| *qRKPS4-6* | MPH | E3 | 4 | 104 | 101.5-106.5 | *PZE-104090796* | *PZE-104093153* | 3.58 |  | 0.01 |  | 4.44 |
| *qRKPS5-1* | IF2 | E4 | 5 | 56 | 55.5-56.5 | *PZE-105032498* | *PZE-105032165* | 4.18 | 0.0040 | -0.01 |  | 4.97 |
| *qRKPS5-2* | IF2 | E3 | 5 | 82 | 79.5-83.5 | *PZE-105067537* | *PZE-105078059* | 6.55 | 0.01 | -0.0003 |  | 7.32 |
| *qRKPS5-3* | RIL | E3 | 5 | 117 | 115.5-119.5 | *PZE-105113962* | *PZE-105115759* | 6.03 | 0.01 |  |  | 7.64 |
| *qRKPS5-4* | RIL | E4 | 5 | 122 | 120.5-124.5 | *SYN11021* | *SYN7361* | 2.8 | 0.01 |  |  | 4.05 |
| *qRKPS5-5* | MPH | E3 | 5 | 125 | 124.5-130.5 | *PZE-105119998* | *SYN5396* | 2.58 |  | 0.01 |  | 3.09 |
| *qRKPS6-1* | RIL | E3 | 6 | 8 | 3.5-9.5 | *PUT-163a-94473612-4863* | *PZE-106008406* | 3.65 | 0.01 |  |  | 4.79 |
| *qRKPS6-2* | IF2 | E4 | 6 | 19 | 18.5-20.5 | *PZE-106016936* | *PZE-106019045* | 5.25 | 0.01 | 0.0026 |  | 6.16 |
| *qRKPS6-3* | IF2 | E3 | 6 | 36 | 35.5-37.5 | *PZE-106038001* | *PZE-106038725* | 8.3 | 0.01 | -0.0023 |  | 9.01 |
| *qRKPS6-4* | RIL | E4 | 6 | 103 | 100.5-104.5 | *PZE-106083557* | *PZE-106083588* | 3.3 | -0.02 |  |  | 4.62 |
| *qRKPS6-5* | MPH | E4 | 6 | 106 | 104.5-115.5 | *PZE-106083873* | *PZE-106115356* | 2.53 |  | 0.01 |  | 3.79 |
| *qRKPS7-1* | RIL | E1 | 7 | 56 | 53.5-57.5 | *PZE-107019133* | *PZE-107033682* | 3.85 | -0.03 |  |  | 3.92 |
| *qRKPS7-2* | RIL | E2 | 7 | 59 | 58.5-59.5 | *PZE-107033682* | *PZE-107030398* | 2.83 | -0.02 |  |  | 3.48 |
| *qRKPS7-3* | MPH | E3 | 7 | 73 | 65.5-80.5 | *PZE-107057229* | *PZE-107081317* | 3.19 |  | 0.01 |  | 6.07 |
| *qRKPS7-4* | IF2 | E2 | 7 | 85 | 83.5-88.5 | *PZE-107081254* | *PZE-107084740* | 2.83 | -0.01 | 0.01 |  | 6.28 |
|  | RIL | E4 | 7 | 88 | 86.5-91.5 | *PZE-107084740* | *PZE-107086184* | 5.35 | -0.02 |  |  | 7.76 |
| *qRKPS7-5* | RIL | E1 | 7 | 93 | 92.5-94.5 | *PZE-107088270* | *PZE-107088998* | 3.65 | -0.02 |  |  | 3.47 |
| *qRKPS7-6* | RIL | E3 | 7 | 103 | 102.5-104.5 | *PZE-107094398* | *PZE-107094385* | 5.31 | -0.01 |  |  | 6.54 |
|  | IF2 | E4 | 7 | 103 | 102.5-104.5 | *PZE-107094398* | *PZE-107094385* | 3.75 | -0.01 | 0.0011 |  | 4.37 |
|  | RIL | E2 | 7 | 104 | 102.5-104.5 | *PZE-107094385* | *PZE-107093186* | 2.55 | -0.01 |  |  | 3.1 |
| *qRKPS8-1* | RIL | E1 | 8 | 13 | 10.5-17.5 | *PZE-108002532* | *PZE-108003557* | 4.33 | 0.03 |  |  | 4.3 |
| *qRKPS8-2* | IF2 | E4 | 8 | 32 | 30.5-34.5 | *SYN22840* | *SYN14938* | 3.02 | 0.0017 | 0.01 |  | 4.01 |
| *qRKPS10-1* | MPH | E2 | 10 | 39 | 37.5-39.5 | *PZE-110009748* | *PZE-110010390* | 6.81 |  | 0.01 |  | 8.3 |
| *qRKPS10-2* | RIL | E2 | 10 | 45 | 43.5-46.5 | *PZE-110013181* | *PZE-110014546* | 3.37 | 0.02 |  |  | 4.18 |
| *qRKPS10-3* | RIL | E1 | 10 | 83 | 82.5-86.5 | *SYN17753* | *PZE-110074914* | 4.16 | 0.03 |  |  | 3.93 |

^a^ The nomination of QTL is made as follows: a “q” standing for the abbreviation of QTL, the abbreviation of the trait, an “S” standing for single environment analyses, one number standing for chromosome, and another for physical order. EWPE, ear weight per ear; CWPE, cob weight per ear; ED, ear diameter; CD, cob diameter; EL, ear length; RN, row number; KNPR, kernel number per row; KWPE, kernel weight per row; RKP, rate of kernel production.

^b^ RIL, recombinant inbred lines; IF_2_, the immortalized F_2_; MPH, mid-parental heterosis derived from the RIL and IF_2_ populations.

^c^ Env, environment; E1, 2014Jinghong; E2, 2015Jinghong; E3, 2016Chongzhou; E4, 2016Jinghong.

^d^ Chr, chromosome.

^e^ A, additive effect; Negative additive values indicate that the allele for increasing trait value is contributed by the parent 08-641; Positive additive values indicate that the allele for increasing trait value is contributed by another parent YE478.

^f^ D, dominance effect.

^g^ PVE, phenotypic variance explained by QTL.
