# Supplementary material for: Genetic dissection of yield-related traits and mid-parent heterosis for those traits in maize (Zea mays L.)
Source: BMC Plant Biol. 2019 Sep 9;19:392. doi: 10.1186/s12870-019-2009-2 (PMC6734583; doi:10.1186/s12870-019-2009-2)
Supplement: Supplementary file 7 — Table S5. Pearson’s correlation coefficients between phenotypic performance per se in IF2 population and mid-parent heterosis for those traits. *** Significant at p < 0.0001. (DOCX 15 kb) [file 12870_2019_2009_MOESM7_ESM.docx]

Table S5 Pearson’s correlation coefficients between phenotypic performance *per se* in IF_2_ population and mid-parent heterosis for those traits.

| Trait | Mid-parent heterosis |
| --- | --- |
| EWPE | 0.86^***^ |
| CWPE | 0.76^***^ |
| EL | 0.59^***^ |
| ED | 0.69^***^ |
| CD | 0.64^***^ |
| RN | 0.58^***^ |
| KNPR | 0.64^***^ |
| KWPE | 0.79^***^ |
| RKP | -0.04 |

^***^ Significant at *p* < 0.0001; EWPE, ear weight per ear; CWPE, cob weight per ear; EL, ear length; ED, ear diameter; CD, cob diameter; RN, row number; KNPR, kernel number per row; KWPE, kernel weight per ear; RKP, rate of kernel production.
