# Supplementary material for: Genetic dissection of yield-related traits and mid-parent heterosis for those traits in maize (Zea mays L.)
Source: BMC Plant Biol. 2019 Sep 9;19:392. doi: 10.1186/s12870-019-2009-2 (PMC6734583; doi:10.1186/s12870-019-2009-2)
Supplement: Supplementary file 8 — Table S6. Pearson’s correlation coefficients between phenotypic variation and genome marker heterozygosity. a Correlation between performance per se in IF2 population and percentage of heterozygous loci. b Correlation between mid-parent heterosis and the percentage of heterozygous loci. ***, significant at p < 0.0001 level; **, significant at p < 0.01 level; *, significant at p < 0.05 level; NS, not significant. EWPE, ear weight per ear; CWPE, cob weight per ear; ED, ear diameter; CD, cob diameter; EL, ear length; RN, row number; KNPR, kernel number per row; KWPE, kernel weight per row; RKP, rate of kernel production. (DOCX 16 kb) [file 12870_2019_2009_MOESM8_ESM.docx]

Table S6 Pearson’s correlation coefficients between phenotypic variation and genome marker heterozygosity.

| Trait | Performance *per se* from IF_2_^a^ | Mid-parent heterosis^b^ |
| --- | --- | --- |
| EWPE | 0.23^***^ | 0.25^***^ |
| CWPE | 0.15^**^ | 0.14^*^ |
| EL | 0.23^***^ | 0.21^**^ |
| ED | 0.13^*^ | 0.14^*^ |
| CD | 0.12^*^ | 0.06^NS^ |
| RN | 0.07^NS^ | 0.04^NS^ |
| KNPR | 0.29^***^ | 0.27^***^ |
| KWPE | 0.24^***^ | 0.26^***^ |
| RKP | 0.13^*^ | 0.06^NS^ |

^a^ Correlation between performance per se in IF_2_ population and percentage of heterozygous loci.

^b^ Correlation between mid-parent heterosis and the percentage of heterozygous loci.

^***^, significant at P<0.0001 level; ^**^, significant at P<0.01 level; ^*^, significant at P<0.05 level; NS, not significant. EWPE, ear weight per ear; CWPE, cob weight per ear; ED, ear diameter; CD, cob diameter; EL, ear length; RN, row number; KNPR, kernel number per row; KWPE, kernel weight per row; RKP, the ratio of kernel production.
