# Supplementary material for: Genetic dissection of yield-related traits and mid-parent heterosis for those traits in maize (Zea mays L.)
Source: BMC Plant Biol. 2019 Sep 9;19:392. doi: 10.1186/s12870-019-2009-2 (PMC6734583; doi:10.1186/s12870-019-2009-2)
Supplement: Supplementary file 9 — Table S7. Epistatic interactions for yield-related traits and mid-parent heterosis detected in the RILs and IF2 population across all environments. a EWPE, ear weight per ear; CWPE, cob weight per ear; CD, cob diameter; EL, ear length; RN, row number; KNPR, kernel number per row; KWPE, kernel weight per row; RKP, rate of kernel production. b Interval of the first QTL i. c Chromosome ID at the first scanning position. d A_i Additive effect of the QTL i. e D_i Dominance effect of the QTL i. f Interval of the second QTL j. g Chromosome ID at the second scanning position. h A_j Additive effect of the QTL j. i D_j Dominance effect of the QTL j. j AA: Estimated additive by the additive effect of QTLs at the two scanning positions. k AD: Estimated additive by dominance effect of QTLs at the two scanning positions. m DA: Estimated dominance by the additive effect of QTLs at the two scanning positions. n DD: Estimated dominance by dominance effect of QTLs at the two scanning positions. l h2(%): Heritability for epistatic QTL effects. The interval in bold was co-located in QTLs associated with the same trait via combined analysis across all environments. *, **, *** indicate significance at p < 0.05, p < 0.01, and p < 0.0001, respectively. (DOCX 26 kb) [file 12870_2019_2009_MOESM9_ESM.docx]

Table S7 Epistatic interactions for yield-related traits and mid-parent heterosis detected in the RILs and IF_2_ population across all environments

| Trait^a^ | interval_i^b^ | Chr^c^ | range_i | A_i^d^ | D_i^e^ | interval_j^f^ | Chr^g^ | range_j | A_i^h^ | D_i^i^ | AA^j^ | AD^k^ | DA^m^ | DD^n^ | *h*^2l^ |
| --- | --- | --- | --- | --- | --- | --- | --- | --- | --- | --- | --- | --- | --- | --- | --- |
|  |  |  | (cM) |  |  |  |  | (cM) |  |  |  |  |  |  | (%) |
| The RILs | | | | | | | | | | | | | | | |
| EL | ***PZE-102017443/SYN7604*** | 2 | 0.3-7.3 | -0.48^***^ |  | *PZE-107121485/PZE-107130438* | 7 | 142.9-150.4 | 0.40^***^ |  | -0.18^***^ |  |  |  | 0.63 |
| EL | *SYN8348/PZE-102186160* | 2 | 190.0-195.3 | 0.18^***^ |  | *PZE-105098019/PZE-105096701* | 5 | 95.2-96.5 | 0.35^***^ |  | 0.17^**^ |  |  |  | 0.61 |
| RN | *SYN34894/SYN13599* | 2 | 98.0-99.1 | -0.15^***^ |  | *PZE-103150482/SYN28063* | 3 | 159.1-163.1 | 0.24^***^ |  | -0.15^***^ |  |  |  | 1 |
| RN | ***PZE-102186160/SYN14631*** | 2 | 200.3-203.3 | -0.10^**^ |  | ***PZE-109008839/PZE-109015923*** | 9 | 17.9-26.2 | 0.35^***^ |  | 0.12^***^ |  |  |  | 0.57 |
| RN | *PZE-107019133/PZE-107033682* | 7 | 53.6-58.4 | -0.20^***^ |  | ***PZE-109057210/PZB01899.2*** | 9 | 95.2-97.1 | -0.38^***^ |  | 0.13^***^ |  |  |  | 1.03 |
| RKP | ***PZE-101184757/SYN25826*** | 1 | 168.3-171.8 | -0.01^***^ |  | ***SYN23245/PZE-103132112*** | 3 | 138.2-142.4 | 0.02^***^ |  | -0.01^***^ |  |  |  | 0.05 |
| RKP | ***PZE-101184757/SYN25826*** | 1 | 168.3-171.8 | -0.01^***^ |  | *PZE-103150482/SYN28063* | 3 | 159.1-165.0 | 0.01^***^ |  | 0.02^***^ |  |  |  | 1.1 |
| RKP | ***PZE-101184757/SYN25826*** | 1 | 168.3-171.8 | -0.01^***^ |  | ***PZE-107094385/PZE-107093186*** | 7 | 103.2-104.3 | -0.02^***^ |  | -0.01^**^ |  |  |  | 0.41 |
| RKP | *PZE-103150482/SYN28063* | 3 | 159.1-165.0 | 0.01^***^ |  | *PZE-104050647/SYN11159* | 4 | 60.8-62.6 | -0.02^***^ |  | 0.02^***^ |  |  |  | 1.52 |
| RKP | *PZE-103150482/SYN28063* | 3 | 159.1-165.0 | 0.01^***^ |  | ***PZE-106083873/PZE-106115356*** | 6 | 105.2-124.2 | -0.02^***^ |  | 0.01^***^ |  |  |  | 0.75 |
| RKP | ***PZE-108002532/PZE-108003557*** | 8 | 10.6-13.6 | 0.01^***^ |  | ***PZE-110007326/PZE-110008811*** | 10 | 29.0-34.3 | 0.02^***^ |  | -0.01^***^ |  |  |  | 0.57 |
| EWPE | *SYN14143/PZE-101019726* | 1 | 3.3-9.3 | -1.64^**^ |  | *PZE-107012245/SYN24186* | 7 | 41.2-48.2 | -2.56^***^ |  | -2.55^***^ |  |  |  | 1.47 |
| KNPR | *PZE-106083873-PZE-106115356* | 6 | 105.2-120.2 | -1.05^***^ |  | *PZE-107089819/PZE-107094078* | 7 | 96.5-101.5 | -0.80^***^ |  | 0.65^**^ |  |  |  | 0.73 |
| IF_2_ population | | | | | | | | | | | | | | | |
| CWPE | ***SYN450/SYN25114*** | 1 | 51.8-54.1 | -1.01^***^ | -0.75^**^ | ***PZE-110051403/PZE-110054264*** | 10 | 71.3-74.3 | 0.16 | 0.99^***^ | 0.94^***^ |  |  |  | 1.65 |
| CWPE | *PZE-105101867/PZE-105102442* | 5 | 100.4-102.4 | 0.88^***^ | 0.43 | *PZE-106083873/PZE-106115356* | 6 | 111.2-128.5 | 1.23^***^ | 1.11^*^ |  |  | 0.89^*^ |  | 0.76 |
| EL | ***PZE-101213558/SYN22772*** | 1 | 201.9-211.9 | -0.45^***^ | 0.31^*^ | *PZE-102080894/PZE-102079520* | 2 | 75.3-80.7 | -0.44^***^ | 0.39^***^ | 0.46^***^ |  |  |  | 0.22 |
| EL | ***PZE-101213558/SYN22772*** | 1 | 201.9-211.9 | -0.45^***^ | 0.31^*^ | *PZE-108098384/PZE-108110152* | 8 | 118.6-122.5 | 0.21^***^ | 0.49^***^ |  |  | 0.83^***^ | 0.59^*^ | 1.01 |
| EL | ***PZE-101213558/SYN22772*** | 1 | 201.9-211.9 | -0.45^***^ | 0.31^*^ | ***PZE-110007326/PZE-110008811*** | 10 | 31.0-37.3 | 0.57^***^ | 0.12 | -0.52^***^ |  | -0.43^*^ | -1.01^**^ | 0.62 |
| EL | *PZE-102080894/PZE-102079520* | 2 | 75.3-80.7 | -0.44^***^ | 0.39^***^ | *PZE-104010113/PZE-104012412* | 4 | 1.0-8.9 | -0.52^***^ | 0.46^***^ | 0.21^*^ |  | 0.54^***^ | 0.40^*^ | 1.46 |
| EL | *PZE-102080894/PZE-102079520* | 2 | 75.3-80.7 | -0.44^***^ | 0.39^***^ | ***PZE-110007326/PZE-110008811*** | 10 | 31.0-37.3 | 0.57^***^ | 0.12 | -0.26^**^ |  |  | -0.65^***^ | 1.33 |
| CD | ***SYN25114/PZE-101055771*** | 1 | 51.8-58.2 | -0.60^***^ | -0.11 | ***PZE-107088218/PZE-107088270*** | 7 | 89.9-92.8 | 0.37 | 0.49^***^ |  | -0.97^***^ |  |  | 0.09 |
| RN | ***SYN16390/SYN8399*** | 2 | 102.8-104.9 | -0.36^***^ | 0.10 | *PZE-104012412/SYN8509* | 4 | 1.0-10.9 | -0.03 | -0.31^***^ | 0.33^***^ | 0.27^*^ |  |  | 0.29 |
| KWPE | ***PZE-102065424/PZA02450.1*** | 2 | 57.2-63.0 | -1.78^*^ | 7.53^***^ | ***PZE-107019133/PZE-107033682*** | 7 | 52.6-58.4 | -3.85^***^ | 9.45^***^ |  |  | -6.34^**^ | 7.83^**^ | 1.13 |
| The MPH dataset | | | | | | | | | | | | | | | |
| EL | ***SYN275/PZE-101213558*** | 1 | 186.0-204.9 | -0.34^***^ | -0.04 | *SYN7604/PZE-102037260* | 2 | 16.1-30.6 | 0.23^*^ | 0.38^*^ |  |  | -0.96^***^ |  | 0.87 |
| EL | ***SYN275/PZE-101213558*** | 1 | 186.0-204.9 | -0.34^***^ | -0.04 | *PZE-105081098/PZE-105082252* | 5 | 85.2-90.5 | 18.12^***^ | -18.66^***^ | 0.44^***^ |  |  |  | 0.58 |
| EL | *PZE-105081098/PZE-105082252* | 5 | 85.2-90.5 | 18.12^***^ | -18.66^***^ | *PZE-105109096/PZE-105110168* | 5 | 104.9-109.7 | -18.40^***^ | -18.22^***^ | -37.09^***^ | -37.21^***^ | 37.15^***^ | 36.26^***^ | 0.93 |
| KWPE | *PZE-102017443/SYN7604* | 2 | 3.4-13.4 | 5.10^*^ | 10.10^**^ | ***SYN6986/ZM012337-0431*** | 3 | 200.9-211.3 | -5.41^**^ | 6.20^*^ | -10.00^**^ |  |  |  | 0.16 |
| RKP | ***PZE-103136534/SYN15014*** | 3 | 146.9-159.0 | -0.02^***^ | -0.004 | ***PZE-104049163/PZE-104050646*** | 4 | 58.3-62.6 | 0.02^***^ | 0.006 | -0.02^***^ |  |  |  | 0.11 |

^a^ EWPE, ear weight per ear; CWPE, cob weight per ear; CD, cob diameter; EL, ear length; RN, row number; KNPR, kernel number per row; KWPE, kernel weight per row; RKP, rate of kernel production.

^b^ Interval of the first QTL i.

^c^ Chromosome ID at the first scanning position.

^d^ A_i Additive effect of the QTL i.

^e^ D_i Dominance effect of the QTL i.

^f^ Interval of the second QTL j.

^g^ Chromosome ID at the second scanning position.

^h^ A_j Additive effect of the QTL j.

^i^ D_j Dominance effect of the QTL j.

^j^ AA: Estimated additive by the additive effect of QTLs at the two scanning positions.
^k^ AD: Estimated additive by dominance effect of QTLs at the two scanning positions.
^m^ DA: Estimated dominance by the additive effect of QTLs at the two scanning positions.
^n^ DD: Estimated dominance by dominance effect of QTLs at the two scanning positions.

^l^ *h*^2^(%): Heritability for epistatic QTL effects.

The interval in bold was co-located in QTLs associated with the same trait via combined analysis across all environments.

^*^, ^**^, ^***^ indicate significance at *p* < 0.05, *p* < 0.01, and *p* < 0.0001, repsectively.
