# Supplementary material for: Genetic dissection of yield-related traits and mid-parent heterosis for those traits in maize (Zea mays L.)
Source: BMC Plant Biol. 2019 Sep 9;19:392. doi: 10.1186/s12870-019-2009-2 (PMC6734583; doi:10.1186/s12870-019-2009-2)
Supplement: Supplementary file 10 — Table S8. QTL × environment interactions for yield-related traits detected in the RILs under four environments and the IF2 population under three environments. a RIL, recombinant inbred lines; IF2, the immortalized F2. b AE indicates the additive by designed environment interaction effect. E1, E2, E3, and E4, represent 2014JH, 2015JH, 2016CZ, and 2016JH, respectively. c h2(AE, %) is the contribution rate of additive by environment interaction effect. d DE indicates the dominance by designed environment interaction effect. E1, E2, E3, and E4, represent 2014JH, 2015JH, 2016CZ, and 2016JH, respectively. e h2(DE, %) is the contribution rate of dominance by environment interaction effect. *, **, *** indicate significance at p < 0.05, p < 0.01, and p < 0.0001, respectively. The interval in bold was co-located in QTLs associated with the same trait via combined analysis across all environments. (DOCX 21 kb) [file 12870_2019_2009_MOESM10_ESM.docx]

Table S8 QTL × environment interactions for yield-related traits detected in the RILs under four environments and the IF_2_ population under three environments.

| Data^a^ | Trait | Chr | Site  (cM) | Interval | AE1^b^ | AE2 ^b^ | AE3 ^b^ | AE4 ^b^ | *h*^2^(AE, %) ^c^ | DE1^d^ | DE2^d^ | DE3^d^ | DE4^d^ | *h*^2^(DE, %) ^e^ |
| --- | --- | --- | --- | --- | --- | --- | --- | --- | --- | --- | --- | --- | --- | --- |
| IF_2_ | EWPE | 1 | 56.1 | *SYN25114/PZE-101055771* |  |  | 3.33^**^ |  | 1.0 |  |  |  |  |  |
| RIL | EWPE | 6 | 114.2 | *PZE-106083873/PZE-106115356* | -2.76^**^ |  |  |  | 1.2 |  |  |  |  |  |
| RIL | EWPE | 7 | 43.2 | *PZE-107012245/SYN24186* |  | 2.48^*^ |  |  | 1.2 |  |  |  |  |  |
| RIL | CWPE | 5 | 120.3 | *PZA00987.1/PZE-105116229* |  |  | -0.51^*^ |  | 1.2 |  |  |  |  |  |
| IF_2_ | CWPE | 6 | 20.8 | *PZE-106018356/PZE-106017695* |  | -0.65^*^ |  |  | 1.0 |  |  |  |  |  |
| RIL | CD | 2 | 96.6 | *SYN11831/PZE-102112161* |  |  |  | 0.25* | 1.0 |  |  |  |  |  |
| IF_2_ | KWPE | 2 | 60.4 | ***PZE-102065424/PZA02450.1*** |  |  |  |  |  |  | 3.87^*^ |  |  | 0.84 |
| RIL | RKP | 3 | 141.2 | ***SYN23245/PZE-103132112*** | 0.02^***^ |  |  |  | 1.1 |  |  |  |  |  |
| RIL | RKP | 7 | 57.6 | ***PZE-107019133/PZE-107033682*** | -0.01^*^ |  |  |  | 0.8 |  |  |  |  |  |
| RIL | RKP | 8 | 11.6 | ***PZE-108002532/PZE-108003557*** | 0.02^**^ |  |  |  | 1.0 |  |  |  |  |  |

^a^ RIL, recombinant inbred line; IF_2_, the immortalized F_2_.

^b^ AE indicates the additive by designed environment interaction effect. E1, E2, E3, and E4, represent 2014JH, 2015JH, 2016CZ, and 2016JH, respectively.

^c^ *h*^2^(AE, %) is the contribution rate of additive by environment interaction effect.

^d^ DE indicates the dominance by designed environment interaction effect. E1, E2, E3, and E4, represent 2014JH, 2015JH, 2016CZ, and 2016JH, respectively.

^e^ *h*^2^(DE, %) is the contribution rate of dominance by environment interaction effect.

^*^, ^**^, ^***^ indicate significance at *p* < 0.05, *p* < 0.01, and *p* < 0.0001, respectively.

The interval in bold was co-located in QTLs associated with the same trait via combined analysis across all environments.

EWPE, ear weight per ear; CWPE, cob weight per ear; CD, cob diameter; KWPE, kernel weight per row; RKP, rate of kernel production.
